# Vitamin D regulates microbiome-dependent cancer immunity

**DOI:** 10.1126/science.adh7954

**Published:** 2024-04-25

**Authors:** Evangelos Giampazolias, Mariana Pereira da Costa, Khiem C. Lam, Kok Haw Jonathan Lim, Ana Cardoso, Cécile Piot, Probir Chakravarty, Sonja Blasche, Swara Patel, Adi Biram, Tomas Castro-Dopico, Michael D. Buck, Richard R. Rodrigues, Gry Juul Poulsen, Susana A. Palma-Duran, Neil C. Rogers, Maria A. Koufaki, Carlos M. Minutti, Pengbo Wang, Alexander Vdovin, Bruno Frederico, Eleanor Childs, Sonia Lee, Ben Simpson, Andrea Iseppon, Sara Omenetti, Gavin Kelly, Robert Goldstone, Emma Nye, Alejandro Suárez-Bonnet, Simon L. Priestnall, James I. MacRae, Santiago Zelenay, Kiran Raosaheb Patil, Kevin Litchfield, James C. Lee, Tine Jess, Romina S. Goldszmid, Caetano Reis e Sousa

**Affiliations:** 1 Immunobiology Laboratory, The Francis Crick Institute, 1 Midland Road, London NW1 1AT, UK; 2 Cancer Immunosurveillance Group, Cancer Research UK Manchester Institute, The University of Manchester, Wilmslow Road, Manchester M20 4BX, UK; 3 Inflammatory Cell Dynamics Section, Laboratory of Integrative Cancer Immunology (LICI), Center for Cancer Research (CCR), National Cancer Institute (NCI), 37 Convent Drive, Bethesda, MD 20892-0001, USA; 4 Department of Immunology and Inflammation, Imperial College, London, UK; 5 Bioinformatics and Biostatistics STP, The Francis Crick Institute, 1 Midland Road, London NW1 1AT, UK; 6 MRC Toxicology Unit, University of Cambridge, Gleeson Building, Tennis Court Road, Cambridge, CB2 1QR, UK; 7 Basic Science Program, Frederick National Laboratory for Cancer Research, Frederick, MD, USA; 8 Microbiome and Genetics Core, LICI, CCR, NCI, 37 Convent Drive, Bethesda, MD 20892-0001, USA; 9 National Center of Excellence for Molecular Prediction of Inflammatory Bowel Disease, PREDICT, Faculty of Medicine, Aalborg University, Department of Gastroenterology and Hepatology, Aalborg University Hospital, A.C. Meyers Vænge 15, A DK-2450 Copenhagen, Denmark; 10 Metabolomics STP, The Francis Crick Institute, 1 Midland Road, London NW1 1AT, UK; 11 Cancer Inflammation and Immunity Group, Cancer Research UK Manchester Institute, The University of Manchester, Wilmslow Road, Manchester M20 4BX, UK; 12 Tumor ImmunoGenomics and Immunosurveillance (TIGI) Lab, UCL Cancer Institute, 72 Huntley St, London WC1E 6DD, UK; 13 AhRimmunity Laboratory, The Francis Crick Institute, 1 Midland Road, London NW1 1AT, UK; 14 Experimental Histopathology, The Francis Crick Institute, 1 Midland Road, London NW1 1AT, UK; 15 Department of Pathobiology and Population Sciences, The Royal Veterinary College, Hawkshead Lane, North Mymms, Hatfield, Hertfordshire AL9 7TA, UK; 16 Genetic Mechanisms of Disease Laboratory, The Francis Crick Institute, 1 Midland Road, London NW1 1AT, UK; 17 Institute of Liver and Digestive Health, Division of Medicine, Royal Free Hospital, University College London, London, NW3 2QG, UK

## Abstract

A role for vitamin D in immune modulation and in cancer has been suggested. Here, we report that mice with increased availability of vitamin D display greater immune-dependent resistance to transplantable cancers and augmented responses to checkpoint blockade immunotherapies. Similarly, in humans, vitamin D-induced genes correlate with improved responses to immune checkpoint inhibitor treatment, as well as with immunity to cancer and increased overall survival. In mice, resistance is attributable to the activity of vitamin D on intestinal epithelial cells, which alters microbiome composition, favoring *Bacteroides fragilis* that positively regulate cancer immunity. Our findings indicate a previously unappreciated connection between vitamin D, microbial commensal communities and immune responses to cancer. Collectively, they highlight vitamin D levels as a potential determinant of cancer immunity and immunotherapy success.

The micronutrient vitamin D has an important role in immune modulation and in shaping commensal microbial communities (1–6). Vitamin D has also been studied for its potential role in cancer, with studies showing it can decrease cancer cell proliferation, promote apoptosis, reduce angiogenesis (7–9), and dampen the pro-tumorigenic activity of cancer-associated fibroblasts (10, 11). In some but not all studies, higher blood levels or increased dietary intake of vitamin D have been correlated with a lower incidence of colorectal, breast, prostate and pancreatic tumors and/or decreased cancer mortality (12–21). However, to what extent the activity of vitamin D impacts cancer development, and whether this involves the immune system and/or the microbiome, remains unclear.

Vitamin D (calciferol) is a term that includes both vitamin D_3_ (cholecalciferol) and vitamin D_2_ (ergocalciferol) forms of the vitamin. Vitamin D_3_ is derived from animal-sourced foods or is produced by skin in response to ultraviolet radiation whereas vitamin D_2_ is derived from plants and fungi (22). Irrespective of source, both vitamin D_2_ and D_3_ are converted in the liver and other tissues to 25-hydroxyvitamin D [25-OHD], the main circulating form of vitamin D (22). 25-OHD is then converted primarily in kidney to 1,25-dihydroxy-vitamin D [1,25-(OH)_2_D], which can bind to vitamin D receptor (VDR) to regulate expression of vitamin D-responsive genes (22). Notably, vitamin D and its 25-OHD and 1,25-(OH)_2_D metabolites (collectively called VitD henceforth) are bound by the blood carrier protein “group-specific component” (Gc) globulin, also known as vitamin D binding protein. Gc possesses a domain at its N-terminus with high affinity for 25-OHD and lower affinity for its precursor calciferol and for 1,25-(OH)_2_D (23, 24). Gc binding sequesters VitD, principally 25-OHD, away from tissues, acting as a blood reservoir (24, 25). Despite the prominent role of VitD in calcium homeostasis, *Gc*^*-/-*^ mice (and a rare human patient displaying bi-allelic *GC* loss) do not display bone abnormalities (e.g., rickets or osteomalacia) associated with VitD deficiency (24, 26). Rather, animals lacking Gc globulin display low levels of VitD in blood, which results in more rapid and profound tissue responses to VitD at the expense of low buffering capacity (24).

Cross-presentation of tumor antigens by type 1 conventional dendritic cells (cDC1) is critical for generating anti-cancer CD8^+^ T cells (27, 28). In mice and humans, cDC1 express DNGR-1 (a.k.a. CLEC9A), a receptor that binds to F-actin exposed by dying cells and promotes cross-presentation of antigens within the corpses (29, 30). Previously, we showed that secreted gelsolin (sGSN), an extracellular protein that circulates in plasma and is secreted by tumor cells, severs F-actin and blocks DNGR-1 ligand binding, dampening anti-cancer immunity and the efficacy of immunogenic anti-cancer therapies (31, 32). Interestingly, Gc globulin possesses a C-terminal actin-binding domain and functions as an actin scavenging protein in partnership with sGSN, a role that is independent of VitD buffering (33). We therefore set out to test whether, like sGSN, Gc acts as a barrier to anti-cancer CD8^+^ T cell responses. Here, we show that this is indeed the case but that it is not attributable to actin scavenging but to Gc regulation of VitD availability. We uncover a complex interplay whereby increased VitD levels promote responses from intestinal epithelial cells that modulate the gut microbiome, which in turn acts to potentiate anti-cancer immunity. Remarkably, the effect of increased VitD availability on immune-mediated resistance to cancer can be transferred in dominant fashion to microbiota-replete mice by transplantation of fecal matter or oral inoculation with the bacterium *Bacteroides fragilis* provided dietary vitamin D intake is maintained. In humans, we show that vitamin D levels correlate with lower cancer incidence and that hallmarks of VDR activity are associated with better disease outcomes in cancer patients and improved responses to checkpoint blockade immunotherapy. Overall, our data suggest that VitD can regulate the microbiome and anti-cancer immunity, with possible clinical and public health applications.

## Gc-deficient mice display immune-dependent transmissible tumor resistance

**Fig. 1:**
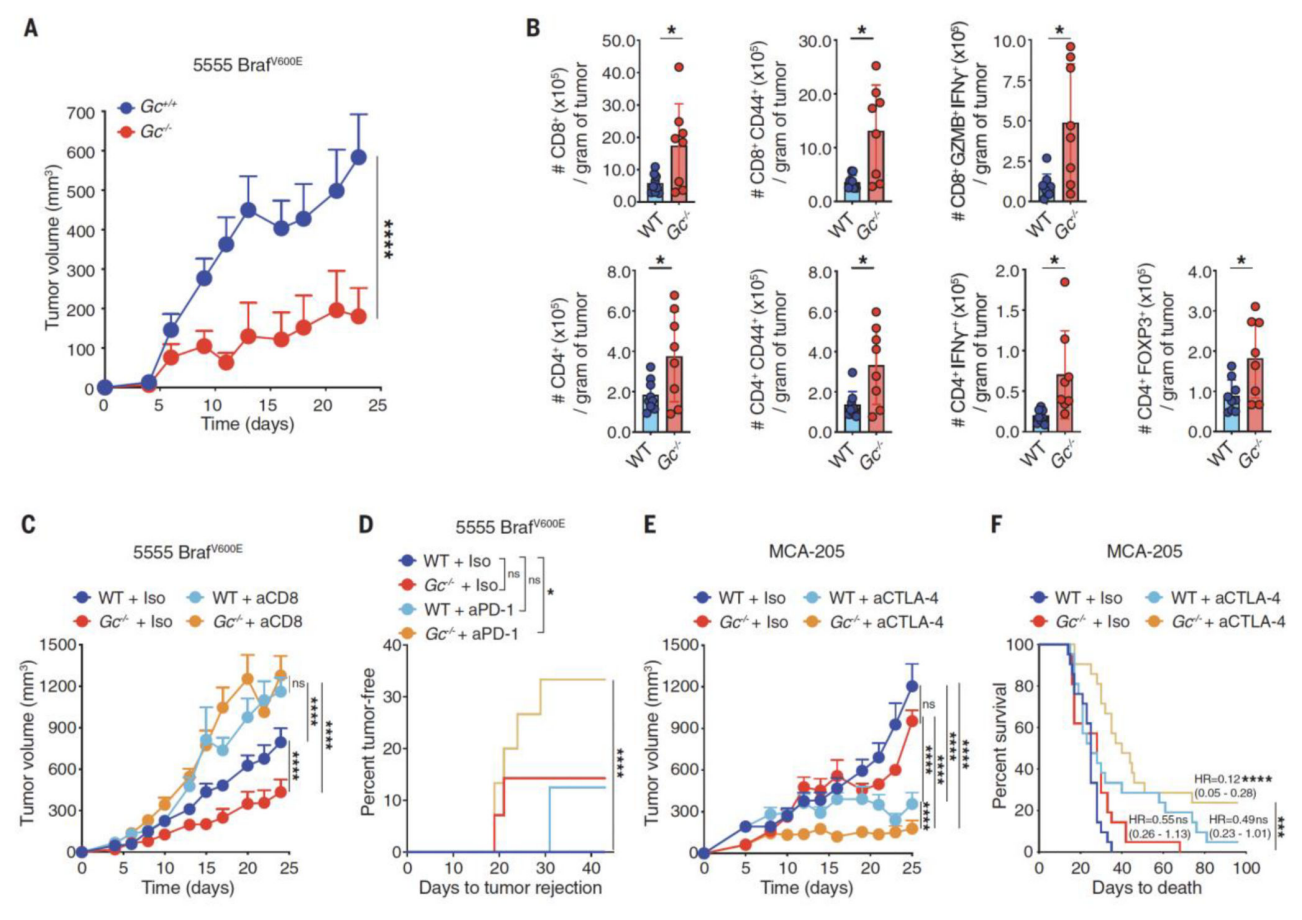
Loss of Gc increases CD8^+^ T cell dependent tumor control and augments response to immunotherapy. (**A**) Growth profile of 0.2 x 10_6_ 5555 Braf^V600E^ cancer cells implanted in separately housed groups of *Gc*^*-/-*^ mice (n=8) and *Gc*^*+/+*^ littermate control mice (n=11). (**B**) Quantification of the indicated intratumoral immune cell populations in separately housed groups of WT C57BL/6J (n=9) or *Gc*^*-/-*^ (n=8) mice at day 15 post-inoculation with 5555 Braf^V600E^ cancer cells. Data are presented as number of cells per gram of tumor from two independent experiments. (**C**) As in (A) but mice received anti-CD8 antibody or isotype-matched control (300μg intraperitoneally (i.p.) on days -3, 1, 4, 7, 10, 13, 16, 19, 22). WT C57BL/6J + isotype (n=12), WT C57BL/6J + anti-CD8 (n=12), *Gc*^*-/-*^ + isotype (n=14) and *Gc*^*-/-*^ + anti-CD8 (n=13). (**D**) Percent of 5555 Braf^V600E^ tumor rejection from two independent experiments in separately housed WT C57BL/6J or *Gc*^*-/-*^ groups of mice that received anti-PD-1 monoclonal antibody or isotype-matched control (200μg i.p. every 3 days from day 3 to day 18). WT + isotype (n=15), WT + anti-PD-1 (n=16), *Gc*^*-/-*^ + isotype (n=14), *Gc*^*-/-*^ + anti-PD-1 (n=15). (**E-F**) Separately housed WT C57BL/6J or *Gc*^*-/-*^ groups of mice implanted with 0.5 x 10^6^ MCA-205 and given isotype-matched control or anti-CTLA-4 (50μg injected i.p. on days 6, 9 and 12). (**E**) Growth profile (n=10 mice per group). (**F**) Survival (Kaplan-Meier) curves from two independent experiments (n=21 mice per group). Data in (A, C and E) are presented as tumor volume (mm^3^) ± SEM and are representative of two independent experiments. Tumor growth profiles (A, C and E) were compared using Bonferroni-corrected two-way ANOVA. Groups in (B) were compared using two-tailed unpaired t test with Welch’s correction. Incidence of tumor rejection and survival (Kaplan-Meier) curves in (D and F) were compared using Log-rank (Mantel-Cox) test for comparison of each group with WT C57BL/6J + isotype and Log-rank for trend for comparison of all groups. In (F) hazard ratios (HR) with 95% confidence interval are shown in brackets, calculated as a ratio of each group / WT + isotype. *p<0.05, ***p< 0.001, ****p< 0.0001; ns, not significant.

We set out to test whether Gc, like sGSN, acts as a barrier to anti-cancer immunity. We used the transplantable 5555 Braf^V600E^ melanoma cell line, the growth of which is greatly attenuated in *sGsn*^*-/-*^ mice (31) and examined its ability to grow in *Gc*^*-/-*^ mice (24) vs. *Gc* ^*+/+*^ littermate controls that were separated at weaning and housed in different cages. Gc-deficient mice (fully backcrossed to the C57BL/6J background) controlled the 5555 Braf^V600E^ melanoma cell line significantly better than Gc-sufficient littermate controls (Fig. 1A) and displayed greater intra-tumoral accumulation of total and activated CD4^+^ and CD8^+^ T cells (Fig. 1B). The relative resistance of *Gc*^*-/-*^ mice to 5555 Braf^V600E^ melanoma was abrogated by antibody-mediated CD8^+^ T cell depletion (Fig. 1C). Additionally, *Gc*^*-/-*^ mice bearing 5555 Braf^V600E^ melanoma or MCA-205 fibrosarcoma tumors displayed greater responses to anti-PD1 and anti-CTLA-4 checkpoint blockade immunotherapies than C57BL6/J wild type (WT) mice (Fig. 1D-F). Thus, like *sGsn*^*-/-*^, *Gc*^-/-^ mice exhibit enhanced CD8^+^ T cell-dependent resistance to transplantable tumors and superior responsiveness to checkpoint blockade immunotherapies.

**Fig. 2:**
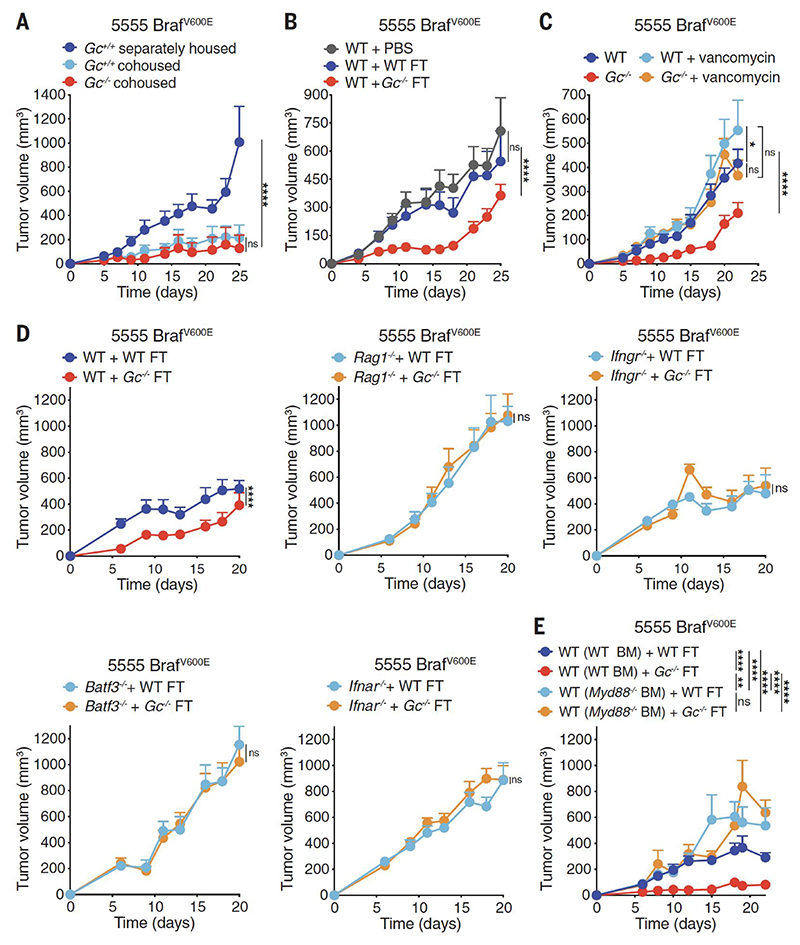
Fecal transplants from *Gc*^*-/-*^ mice increase anti-cancer immunity. (**A-E**) Growth profile of 0.2 x 10^6^ 5555 Braf^V600E^ cancer cells implanted into: (**A**) Separately housed *Gc*^*+/+*^ (n=12) and co-housed *Gc*^*+/+*^ (n=7) and *Gc*^*-/-*^ (n=6) groups of mice. (**B**) Separately housed groups of WT C57BL/6J mice (n=10 per group) that received orally PBS or fecal transplant (FT) from WT or *Gc*^*-/-*^ donors twice (days -14 and - 12) before tumor inoculation (day 0). (**C**) Separately housed groups of WT C57BL/6J or *Gc*^*-/-*^ mice that received or not vancomycin (0.5 g/L) in the drinking water starting from 2 weeks prior to tumor inoculation. WT (n=11), WT + vancomycin (n=10), *Gc*^*-/-*^ (n=11), *Gc*^*-/-*^ + vancomycin (n=10). (**D-E**) the indicated separately housed groups of mice that received oral FT from WT C57BL/6J or *Gc*^*-/-*^ donors twice (days -14 and -12) prior to tumor inoculation (day 0). (**D**) WT (n=11 per group), *Rag1*^*-/-*^ (n=9 per group), *Ifngr1*^*-/-*^ (n=10 per group), *Batf3*^*-/-*^ (n=10) and *Ifnar*^*-/-*^ (n=10 per group) mice, (**E**) Irradiated CD45.1 WT mice reconstituted using bone marrow (BM) from CD45.2 WT or *Myd88*^*-/-*^ donors. WT (WT BM) + WT FT (n=11), WT (WT BM) + *Gc*^*-/-*^ FT (n=12), WT (*Myd88*^*-/-*^ BM) + WT FT (n=10), WT (*Myd88*^*-/-*^ BM) *Gc*^*-/-*^ FT (n=10). Data in (A-E) are presented as tumor volume (mm^3^) ± SEM and are representative of two independent experiments. Tumor growth profiles (A-E) were compared using Bonferroni-corrected two-way ANOVA. *p<0.05, **p<0.01, ***p< 0.001, ****p< 0.0001; ns, not significant.

To control for possible differences in microbiota between *Gc*^*-/-*^ mice and *Gc* ^*+/+*^ controls separated at weaning, we repeated the experiments in *Gc*^*-/-*^ and *Gc* ^*+/+*^ littermates kept in the same cages. Intriguingly, co-housed *Gc* ^*+/+*^ mice acquired the tumor resistance phenotype of their Gc-deficient littermates (Fig. 2A). Similarly, C57BL6/J WT mice (bred as an independent line) became more resistant to tumor challenge when co-housed with *Gc*^*-/-*^ mice (Supplementary Fig. 1A). This transmissible tumor resistance was reversible as *Gc* ^*+/+*^ littermate controls co-housed since birth with *Gc*^*-/-*^ mice were less able to control tumors when separated for at least a month before tumor challenge (Fig. 1A and 2A). These data suggest that: a) *Gc*^*-/-*^ and *Gc* ^*+/+*^ mice exhibit genotype-driven divergence in microbiota composition, which dictates their differential ability to control tumors; b) the *Gc*^*-/-*^-associated component of the microbiota that mediates tumor resistance can be transmitted in a dominant fashion to co-housed mice by coprophagy. Consistent with the latter, fecal transplant (FT) from *Gc*^*-/-*^ donors into microbiota-replete C57BL/6 WT mice led to enhanced tumor control (Fig. 2B). Further, single administration of certain antibiotics (vancomycin, metronidazole or neomycin) inhibited or decreased the ability of *Gc*^*-/-*^ mice to control transplantable tumors (Fig. 2C and Supplementary Fig. 1B).

The anti-tumor effect of the intestinal microbiome of *Gc*^*-/-*^ mice was not accompanied by obvious signs of gut inflammation or histological changes to the intestinal barrier (Supplementary Fig. 1C). Extent of gut-associated lymphoid tissue, gut permeability, total leukocyte numbers, and immune cell composition of intestinal lamina propria were all grossly similar between WT and *Gc*^*-/-*^, except for a decrease in the frequency of IL-17-producing CD4^+^ T cells in the small intestine and of total CD4^+^ T cells and Tregs in the colon of Gc-deficient hosts (Supplementary Fig. 1D-I). Moreover, FT of *Gc*^*-/-*^ fecal matter into WT mice did not increase the severity of dextran sodium sulphate (DSS)-induced colitis (Supplementary Fig. 2A-D). Collectively, these data suggest that the commensal organisms present in the intestine of Gc-deficient mice do not markedly alter barrier function or mucosal immunity, either at steady state or after induction of intestinal inflammation.

To confirm that the transmissible resistance to transplantable tumors was immune-dependent and to dissect the pathways involved, we tested different immune-deficient strains (Fig. 2D and Supplementary Fig. 3A). FT from *Gc*^*-/-*^ donors into mice deficient in T and B cells (*Rag1*^*-/-*^) or IFN-γ receptor (*Ifngr*^*-/-*^) did not confer enhanced protection to subsequent tumor challenge (Fig. 2D). Similarly, mice deficient in CD8^+^ T cells and MHC class I presentation (*Tap1*^*-/-*^) or cDC1 (*Batf3*^*-/-*^) did not display enhanced control of transplantable tumors when given *Gc*^*-/-*^ fecal matter (Fig. 2D and Supplementary Fig. 3A). Global deletion of type I IFN receptor (IFNAR) or MyD88 (an adaptor molecule that operates downstream of IL-1 receptor and Toll-like receptors) also diminished tumor resistance conferred by *Gc*^*-/-*^ FT (Fig. 2D and Supplementary Fig. 3B). Using bone marrow radiation chimeras, MyD88 expression in the hematopoietic compartment was found to be necessary and sufficient for enhanced tumor control (Fig. 2E and Supplementary Fig. 3C). In contrast, the DNA sensor cGAS and the TLR adaptor molecule TRIF were dispensable for increased tumor resistance following *Gc*^*-/-*^ FT administration (Supplementary Fig. 3B). Collectively, these data indicate a key role for innate and adaptive immunity in the enhanced tumor resistance conferred by *Gc*^*-/-*^ microbiota.

## Vitamin D availability determines transmissible tumor resistance in mice

**Fig. 3:**
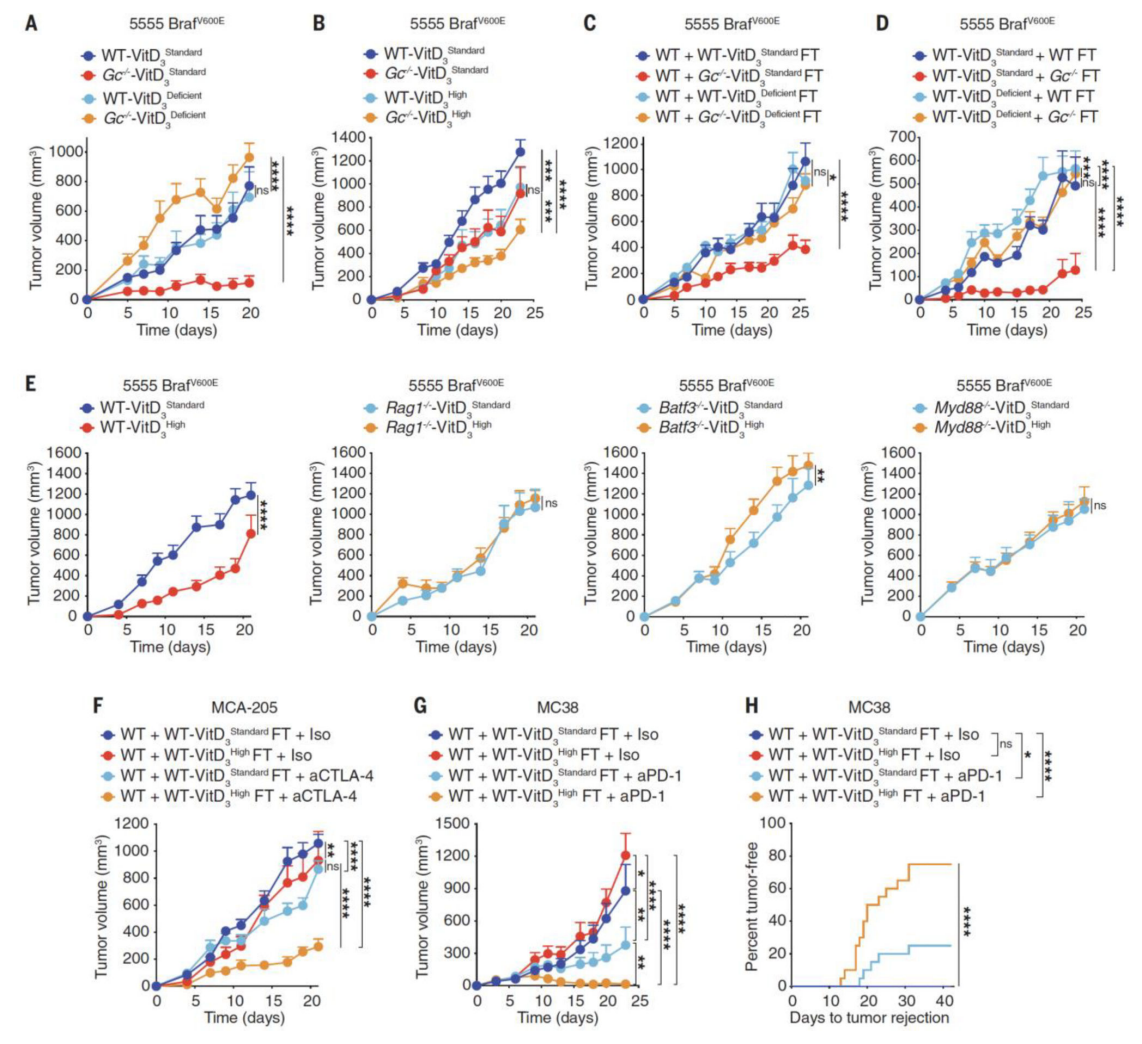
Loss of Gc increases VitD-dependent anti-cancer immunity by altering the gut microbiome. (**A-E**) Growth profile of 0.2 x 10^6^ 5555 Braf^V600E^ cancer cells implanted into: (**A-B**) WT C57BL/6J or *Gc*^*-/-*^ mice that were fed a VitD_3_ standard (2 IU/g), deficient (0 IU/g) or high (10 IU/g) diet starting from 3.5 weeks before tumor inoculation. (**A**) WT + VitD_3_^Standard^ (n=8), WT + VitD_3_^Deficient^ (n=9), *Gc*^*-/-*^ + VitD_3_^Standard^ (n=8), *Gc*^*-/-*^ + VitD_3_^Deficient^ (n=9). (**B**) WT + VitD_3_^Standard^ (n=12), WT + VitD_3_^High^ (n=13), *Gc*^*-/-*^ + VitD_3_^Standard^ (n=12), *Gc*^*-/-*^ + VitD_3_^High^ (n=13). (**C**) WT C57BL/6J (n=10 per group) that received (on days -14 and -12 prior to tumor inoculation) FT from WT C57BL/6J or *Gc*^*-/-*^ donors that had been fed with VitD_3_ standard or deficient diet. (**D**) WT C57BL/6J mice that were fed a VitD_3_ standard or deficient diet starting 3.5 weeks before FT (on days -14 and -12 prior to tumor inoculation) with fecal matter from WT C57BL/6J or *Gc*^*-/-*^ donors. WT-VitD_3_^Standard^ + WT FT (n=7), WT-VitD_3_^Standard^ + WT *Gc*^*-/-*^ (n=10), WT-VitD_3_^Deficient^ + WT FT (n=10), WT-VitD_3_^Deficient^ + WT *Gc*^*-/-*^ FT (n=10). (**E**) WT C57BL/6J, *Rag1*^*-/-*^, *Batf3*^*-/-*^ or *Myd88*^*-/-*^ mice (n=10 per group) that were fed with VitD_3_ standard or high diet starting from 3.5 weeks before tumor inoculation. (**F**) Growth profile of 0.5 x 10^6^ MCA-205 cancer cells implanted into WT C57BL/6J mice (n=10 per group) that received (on days -14 and -12 prior tumor inoculation) FT from WT C57BL/6J donors that were fed with VitD_3_ standard or high diet. Mice were treated i.p. with 50μg of isotype-matched control or anti-CTLA-4 antibody on days 6, 9 and 12. (**G-H**) Separately housed groups of WT C57BL/6J mice implanted with 0.5 x 10^6^ MC38 that received (on days -14 and -12 prior to tumor inoculation) FT from WT C57BL/6J donors that were fed with VitD_3_ standard or high diet. Mice were treated i.p. with 200μg of isotype-matched control or anti-PD-1 monoclonal antibody every 3 days from day 3 to day 12. (**G**) Growth profile (n=10 mice per group). (**H**) Percent tumor rejection from two independent experiments (n=20 mice per group). Data in (A-G) are presented as tumor volume (mm^3^) ± SEM and are representative of two independent experiments. Tumor growth profiles (A-G) were compared using Bonferroni-corrected two-way ANOVA. Incidence of tumor rejection in (H) were compared using Log-rank (Mantel-Cox) test for comparison of each group with WT C57BL/6J + isotype and Log-rank for trend for comparison of all groups. *p<0.05, **p<0.01, ***p< 0.001, ****p< 0.0001; ns, not significant.

Because mice deficient in sGSN do not transfer tumor resistance to co-housed WT mice (31), we hypothesized that a deficiency in actin scavenging was not responsible for the enhanced tumor resistance in *Gc*^*-/-*^ mice. As expected (24), *Gc*^*-/-*^ mice displayed lower levels of vitamin D_3_ and 25-OHD_3_ in plasma, indicative of VitD redistribution to tissues (Supplementary Fig. 4A). The main vitamin D in mouse chow is cholecalciferol (VitD_3_). To test whether Gc deficiency enhances tumor resistance in a VitD-dependent manner, WT and *Gc*^*-/-*^ mice were put on a VitD_3_-deficient diet for approximately 4 weeks to deplete their VitD reservoirs (Supplementary Fig. 4A). Remarkably, this completely abrogated the enhanced ability of *Gc*^*-/-*^ mice to resist tumors (Fig. 3A). In the converse experiment, increased dietary VitD_3_ supplementation led to elevated total VitD serum levels (Supplementary Fig. 4A) and decreased tumor growth in WT mice to the point that they became comparable to Gc-deficient animals fed with standard VitD_3_ chow (Fig. 3B). The latter strain displayed even greater tumor resistance when placed on a VitD_3_ high diet (Fig. 3B). Collectively, these data suggest that enhanced VitD availability, induced by loss of Gc and/or by dietary VitD_3_ supplementation, promotes increased resistance to transplantable tumors in mice.

We next assessed if, as for Gc deficiency, dietary VitD_3_ supplementation increases tumor resistance via the microbiota. Consistent with that notion, a VitD_3_ high diet did not increase the ability of germ-free mice to resist tumors (Supplementary Fig. 4B). Further, the capacity to transmit increased tumor resistance to WT mice was abrogated when fecal material was derived from *Gc*^*-/-*^ mice that had been placed on a VitD_3_-deficient diet (Fig. 3C). Conversely, increasing dietary VitD_3_ in WT mice conferred their fecal matter the ability to transmit tumor control, which was prevented by treatment with vancomycin (Supplementary Fig. 4C and D). Importantly, FT from WT mice that were fed with VitD_3_ high diet transferred tumor resistance to C576BL/6 mice from three different sources that were imported and housed in geographically-distinct animal units (Supplementary Fig. 4E). Finally, we established that VitD availability in the recipient mice, was also necessary for the beneficial anti-tumor effects of FT from *Gc*^*-/-*^ donors. Indeed, enhanced resistance to tumors was prevented if the recipients were placed on a VitD_3_ deficient diet (Fig. 3D).

In parallel, we tested whether manipulation of dietary VitD_3_ impacted tumor growth by modulating cancer immunity. Like Gc deficient hosts (Fig. 1C) or WT mice gavaged with *Gc*^*-/-*^ fecal matter (Fig. 2D-E and Supplementary Fig. 3A and B), mice fed with a VitD_3_ high diet did not exhibit increased tumor resistance if rendered deficient in T and B cells, cDC1 or MyD88 (Fig. 3E). Further resembling *Gc*^*-/-*^ mice, fecal transplants from WT mice that were fed with VitD_3_ high diet increased the therapeutic efficacy of anti-CTLA-4 and anti-PD-1 immune checkpoint blockade inhibitors in transplantable cancer models other than 5555 Braf^V600E^ melanoma such as MCA-205 and MC38 (Fig. 3F, G, H). Collectively, these results establish that 1) high VitD levels favor a mouse microbiome that augments anti-cancer immunity; and 2) the favorable effect can be transferred by FT as long as VitD remains available to the recipient mice.

## Increased vitamin D levels in mice favor a microbiome that potentiates cancer immunity

**Fig. 4:**
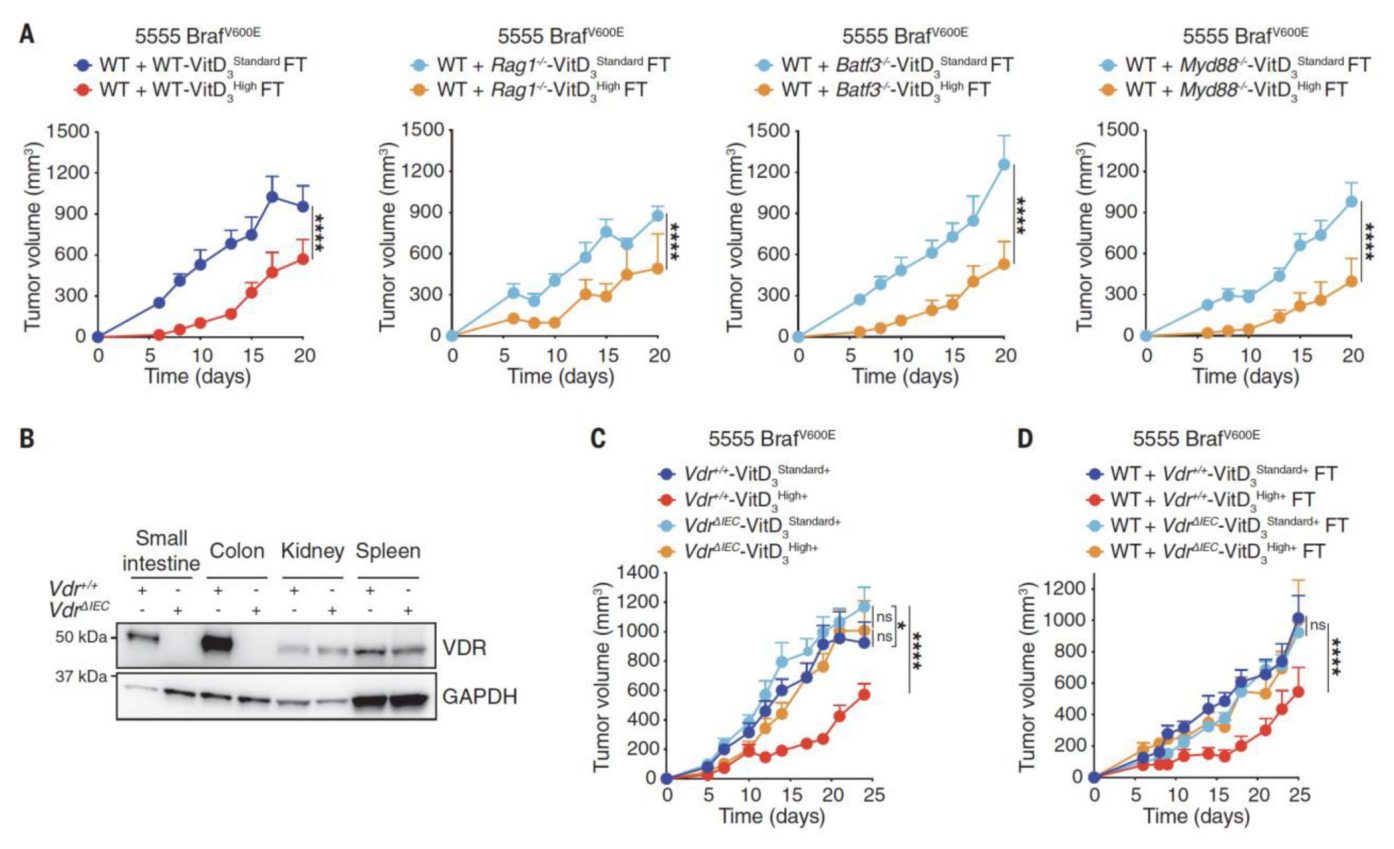
VitD acts via VDR in the gut epithelium to alter gut microbiome and permit tumor control. (**A, C-D**) Growth profile of 0.2 x 10^6^ 5555 Braf^V600E^ cancer cells implanted into: (**A**) WT C57BL/6J mice that received (on days -14 and -12 prior to tumor inoculation) FT from WT C57BL/6J, *Rag1*^*-/-*^, *Batf3*^*-/-*^ or *Myd88*^*-/-*^ donors that had been fed for 3.5 weeks on a VitD_3_ standard or VitD_3_ high diet. WT + WT-VitD_3_^Standard^ FT (n=10), WT + WT-VitD_3_^High^ FT (n=10), WT *+ Rag1*^*-/-*^*-* VitD_3_^Standard^ FT (n=10), WT + *Rag1*^*-/-*^-VitD_3_^High^ FT (n=9), WT + *Batf3*^*-/-*^-VitD_3_^Standard^ FT (n=11), WT + *Batf3*^*-/-*^-VitD_3_^High^ FT (n=11), WT + *Myd88*^*-/-*^-VitD_3_^Standard^ FT (n=9), WT + *Myd88*^*-/-*^-VitD_3_^High^ FT (n=9). (**B**) Lysates from the indicated mouse tissues of *Vdr*^*+/+*^ and *Vdr*^*Δ IEC*^ mice immunoblotted for VDR and GAPDH. (**C**) *Vdr*^*+/+*^ or *Vdr*^*ΔIEC*^ mice kept on a VitD_3_ standard^+^ (2 IU/g) diet complemented with 2% calcium, 1.25% phosphorus and 20% lactose were then maintained on the same diet or switched to a VitD_3_ high^+^ (10 IU/g) diet (similarly complemented with 2% calcium, 1.25% phosphorus and 20% lactose) from 3.5 weeks before tumor inoculation. *Vdr*^*+/+*^-VitD_3_^Standard+^ (n=12), *Vdr*^*+/+*^-VitD_3_^High+^ (n=11), *VdrΔIEC* -VitD_3_^Standard+^ (n=15), *VdrΔIEC* - VitD_3_^High+^ (n=15) (**D**) WT C57BL/6J mice (n=10 per group) received (on days -14 and -12 prior to tumor inoculation) FT from the groups in (C), i.e., *Vdr^+/+^* or *Vdr^ΔIEC^* donors that were fed with VitD_3_ standard^+^ or VitD_3_ high^+^ diet. Data in (A, C and D) are presented as tumor volume (mm^3^) ± SEM and are representative of two independent experiments. Tumor growth profiles (A, C and D) were compared using Bonferroni-corrected two-way ANOVA. *p<0.05, ****p< 0.0001; ns, not significant.

**Fig. 5:**
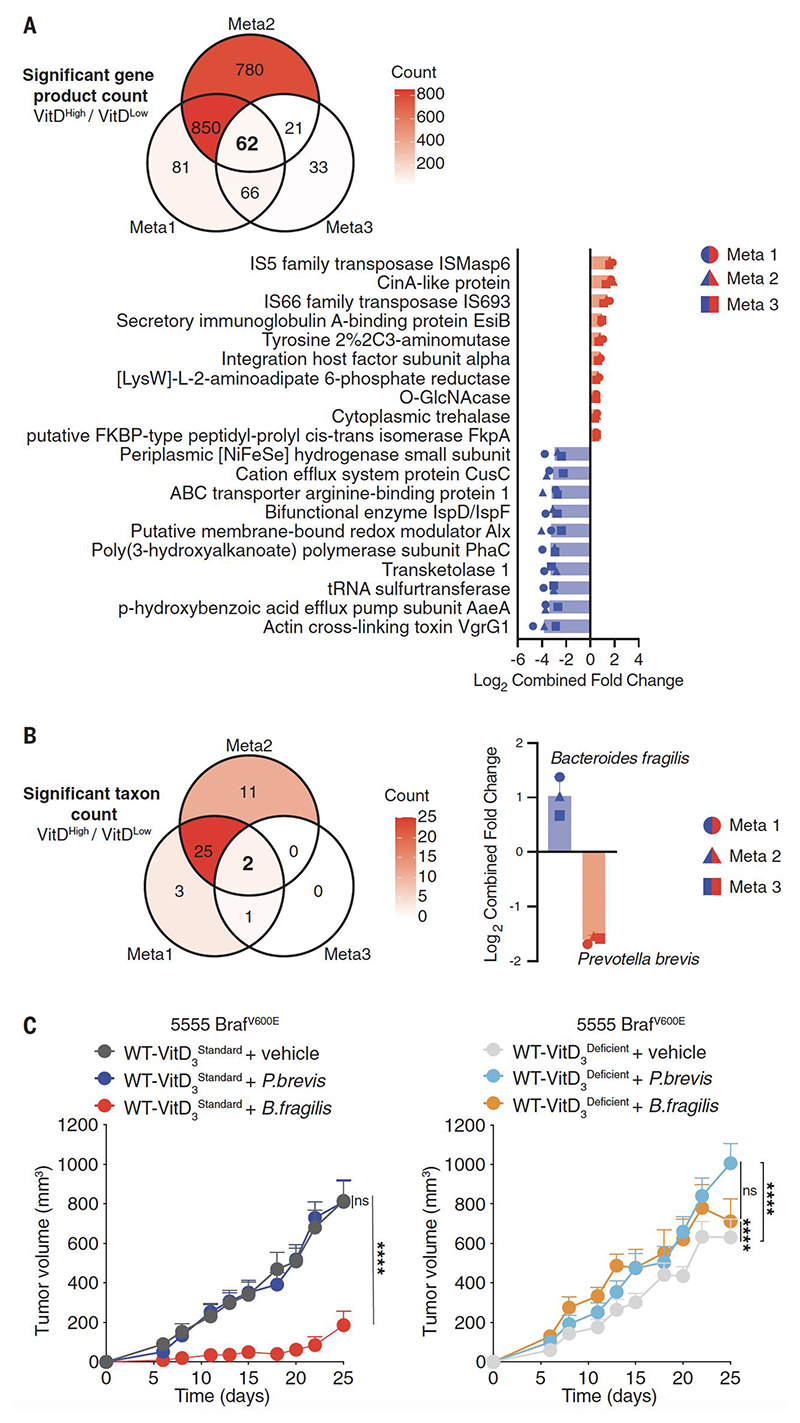
*B.fragilis* promotes tumor resistance in a VitD-dependent manner. (**A-B**) Meta-analysis of metagenomic data to determine (**A**) common features in microbial gene products (top 20/62 features in each direction shown, 20/62) and (**B**) last known taxon associated with differences in VitD availability. Fecal samples were sequenced from WT or *Gc*^*-/-*^ mice that had been fed with VitD_3_ standard (2 IU/g), deficient (0 IU/g) or high (10 IU/g) diet for 3.5 weeks. Comparison is of mice with high VitD availability [WT + VitD_3_^High^ (n=13), *Gc*^*-/-*^ + VitD_3_^Standard^ (n=20), *Gc*^*-/-*^ + VitD_3_^High^ (n=13) vs. mice with normal or low VitD availability [WT + VitD_3_^Standard^ (n=22), WT + VitD_3_^Deficient^ (n=10), *Gc*^*-/-*^ + VitD_3_^Deficient^ (n=10)]. In (A, B), count of significant features indicated in the Venn diagram and shown by color scale (top) and ranked bar plots (bottom) show common features across 3 meta-analyses as indicated. (**C**) Growth profile of 0.2 x 10^6^ 5555 Braf^V600E^ cancer cells implanted into separately housed WT C57BL/6 groups of mice (n=10 per group) fed with VitD_3_ standard (left graph) or deficient diet (right graph), starting 3.5 weeks before receiving *B. fragilis, P. brevis* or vehicle. Mice received 10^9^ *B. fragilis* or *P. brevis* by oral gavage on days -14, -12 and -10 prior to tumor inoculation. Data in (A, B) are presented as average log_2_ median fold change from three meta-analyses of data from two independent experiments. Data in (C) are presented as tumor volume (mm^3^) ± SEM and are representative of two independent experiments for *P. brevis* and 3 independent experiments for *B. fragilis*. In (A, B), p values were calculated using the Mann–Whitney–Wilcoxon U test on parts per million (PPM) relative abundances for that feature in samples within each group for pairwise comparisons. The combined p value (cp) for meta-analysis of within-group comparisons was calculated using Fishers P value. For each feature type, the cut-offs for the meta-analysis were: p< 0.2, cp< 0.1, false discovery rate (FDR)<0.15. Tumor growth profiles (A, C and D) were compared using Bonferroni-corrected two-way ANOVA. ****p< 0.0001; ns, not significant.

The fact that *Rag1*^*-/-*^, *Batf3*^*-/-*^ or *Myd88*^*-/-*^ mice did not display VitD-driven increased immune resistance to cancer (Fig. 3E) was not because immune defects in those mice compromised the ability of VitD_3_ high diet to promote the favorable alterations in microbiota. Indeed, fecal matter from all the immunodeficient mice given VitD_3_ high diets was able to induce greater tumor resistance upon FT into WT mice (Fig. 4A). These data suggest that the ability of high VitD availability to alter the microbiome is largely independent of the immune system. To look for a non-immune component, we turned our attention to the possible effects of VitD on intestinal epithelial cells (IECs). Although it did not alter gut permeability (Supplementary Fig. 1E), a VitD_3_ high diet induced profound changes in gene expression in colonic tissue of WT mice (Supplementary Fig. 5A). Gene expression analysis did not reveal marked compositional differences in specific immune cell populations, as predicted, but alterations in cellular signaling, cell junction organization, as well as in defense from microbes (Supplementary Fig. 5B-D). This is consistent with the ability of VitD, acting via VDR, to directly regulate the expression of multiple genes that impact host physiology (22, 34). To directly assess the importance of VDR in intestinal epithelial cells (IECs), we bred *Vdr*^*fl/fl*^ mice to *Villin*^*Cre*^ mice to generate a *Vdr*^*ΔIEC*^ strain that lacks VDR expression in IECs (Fig. 4B). Upon weaning, VDR^ΔIEC^ mice were maintained on diets complemented with calcium, phosphorus and lactose to mitigate the osteomalacia-like effects of abrogating VitD responsiveness in gut epithelium (35). This altered diet did not prevent the ability of VitD_3_ supplementation to increase tumor resistance in control WT mice [Fig. 4C, VitD_3_ high^+^ diet (where the + symbol denotes calcium/phosphorus/lactose complementation)]. However, VitD_3_ high^+^ diet failed to increase tumor resistance in littermate VDR^ΔIEC^ mice (Fig. 4C). Furthermore, the fecal matter of VDR^ΔIEC^ mice on VitD_3_ high^+^ diet was no longer able to transmit tumor resistance, unlike that of control WT littermates (Fig. 4D). These data indicate that VitD acts via IECs to favor a gut microbiome that increases immune-mediated cancer control.To look for VitD-associated alterations in the microbiome, we carried out shotgun metagenomic analyses of fecal samples from mice in which we altered VitD levels by manipulating diet and/or genotype. We found that bacterial species alpha diversity was largely similar across all samples while beta diversity and taxonomic profiles showed major differences across genotype but not diet (Supplementary Fig. 6A-D, 7A-B, 8A-B). To gain further insight into To gain further insight into bacterial species modulated by VitD availability, we combined 3 meta-analyses of different comparisons across experiments: Meta1, differences driven by genotype (WT vs. *Gc*^*-/-*^) in a VitD_3_^Standard^ condition; Meta2, differences driven by genotype (WT vs. *Gc*^*-/-*^) in the presence of varying levels of dietary VitD (VitD_3_^Standard^ and VitD_3_^High^); Meta3, differences driven by genotype (WT vs. *Gc*^*-/-*^) consistent with those driven by increased dietary VitD in WT (VitD_3_^Standard^ vs. VitD_3_^High^). This approach allowed us to identify 62 gene products and 2 taxa that were consistently regulated by VitD availability across conditions (Fig. 5A-B, Supplementary Fig. 9A-C). Higher VitD availability increased the abundance of *Bacteroides fragilis* at the expense of *Prevotella brevis* (Fig. 5B. Supplementary Fig. 9A-C, 10A-B). Because the ability of *Gc*^*-/-*^ mice to transmit tumour resistance through microbiota depends on the presence of dietary VitD, we removed background differences driven by genotype by contrasting *Gc*^*-/-*^ and WT in mice fed VitD_3_^Deficient^ and VitD_3_^Standard^ diets and focused on taxonomic differences observed exclusively in the presence of VitD (VitD_3_^Standard^). This analysis further confirmed the VitD-dependent increase in *Bacteroides fragilis* and reduction of *P. brevis* (Supplementary Fig. 10C). Therefore, we assessed whether either bacterium could impact tumor resistance in a VitD-dependent manner. Remarkably, three rounds of oral gavage with *Bacteroides fragilis* was sufficient to induce increased resistance to subsequent tumor transplantation across WT C576BL/6 mice procured from different sources and housed in two different animal units (Fig. 5C, left panel, Supplementary Fig. 10D). However, and in line with our earlier data using FT (Fig. 3D), tumor resistance induced by *Bacteroides fragilis* was prevented if the recipient mice were placed on a diet deficient in VitD_3_ (Fig. 5C, right panel). Thus, VitD availability is necessary to maintain a niche in which *Bacteroides fragilis* can thrive. Consistent with that notion, gavage with the bacterium led to slightly lower levels of the organism in the intestine of mice placed on a VitD_3_-deficient diet compared to those on a VitD_3_-standard diet (Supplementary Fig. 10E). In contrast to *Bacteroides fragilis*, gavage with *Prevotella brevis* did not increase tumor resistance (Fig. 5C) and, in fact, decreased it slightly in mice placed on a VitD_3_-deficient diet (Fig. 5C, right panel and Supplementary Fig. 10F).

## Vitamin D levels in humans correlate with cancer resistance

**Fig. 6:**
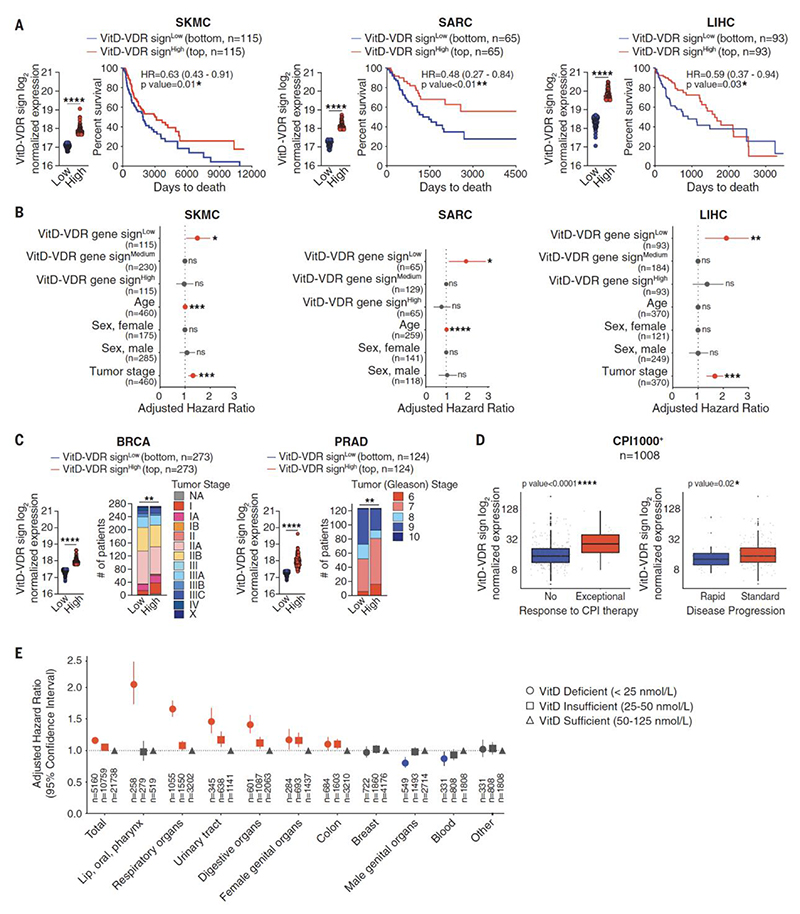
VitD correlates with lower risk of cancer and increased patient survival. (**A**) Prognostic value of VitD-VDR gene signature levels for overall survival and hazard ratio comparing samples with the lowest (VitD-VDR sign^Low^) versus highest (VitD-VDR sign^High^) expression in the indicated TCGA datasets. Skin cutaneous melanoma (SKMC, n=460), sarcoma (SARC, n=259), liver hepatocellular carcinoma (LIHC, n=370), bottom and top 25% of patient cohort. (**B**) Hazard ratio, adjusted for age, sex and tumor stage, comparing samples with the lowest (VitD-VDR sign^Low^) or highest (VitD-VDR sign^High^) versus medium (VitD-VDR sign^Medium^) expression in the indicated TCGA datasets as in (A). (**C**) Prognostic value of VitD-VDR signature levels for tumor stage comparing samples with the lowest (VitD-VDR sign^Low^) versus highest (VitD-VDR = sign^High^) expression in the indicated TCGA datasets. Breast cancer (BRCA, n=1092), prostate adenocarcinoma (PRAD, n=497), bottom and top 25% of patient cohort. (**D**) VitD-VDR signature levels in samples with no response vs. exceptional response (left) and rapid vs. standard disease progression (right) of patients (n=1008) treated with checkpoint inhibitors (CPI1000^+^ cohort). (**E**) Estimated hazard ratio, adjusted for sex, age and Charlson’s comorbidity index, in the VitD deficient (<25 nmol/L) or insufficient (25-50 nmol/L) group versus the VitD sufficient (50-125 nmol/L) group of individuals (n=1,496,766) that were living in Denmark between 2008-2017. In (A) data are presented as mean of log2 normalized expression ± SEM. In (C) data are represented as number of patients that are subdivided based on the tumour stage. In (D) data are presented as log2 normalized expression box-and-whisker plot with median, 25^th^ and 75^th^ percentiles represented by the box and min/max by the whiskers. Survival (Kaplan-Meier) curves in (A) were compared using Log-rank (Mantel-Cox) test. In (A, B and E) hazard ratios (HR) with 95% confidence interval showed. In (A and C) gene signature levels between groups were compared using two-tailed unpaired t test with Welch’s correction. In (C) frequency of tumour stage was compared between groups using Chi-squared test. In (D) expression of gene signature between the groups was compared using Wilcoxon signed-rank test. *p<0.05, **p<0.01, ***p< 0.001, ****p< 0.0001; ns, not significant.

Polymorphisms in genes that encode proteins that participate in 1,25-(OH)_2_D biosynthesis (*CYP2R1, CYP27A1, CYP27B1*), that restrict VitD availability (*GC*) or mediate VitD biological functions (*VDR*) have been variously correlated with cancer risk, alterations in microbiota and/or changes in immune parameters in health and disease (36–40) (https://www.ebi.ac.uk/gwas/; Supplementary Fig. 11A and Supplemental Table 1). *VDR* is an ubiquitously expressed (Supplementary Fig. 11B) nuclear receptor that functions as a ligand-activated transcription factor. We therefore hypothesized that the expression of VDR target genes in any tissue, healthy or malignant, may act as a surrogate measurement of VitD availability in that tissue (24, 41). We assembled a gene signature (VitD-VDR sign) consisting of 237 VDR target genes from several human cell types identified using ChIP-sequencing datasets (Supplemental Table 2; 11, 42–46). We confined our analysis to ChIPseq data to increase resolution and ensure that we analyzed only primary VDR targets even if this might exclude other relevant VitD-inducible genes. We examined the expression of the VitD-VDR sign in different cancers using data from The Cancer Genome Atlas (TCGA) collection (Supplemental Table 2). Analysis of skin cancer (n=460), sarcoma (n=259), liver hepatocellular carcinoma (n=370), breast cancer (n=1092) and prostate adenocarcinoma (n=497) revealed that lower expression of the VitD-VDR signature correlated with poorer survival or more advanced disease (Fig. 6A-C). In the same cancers, the *VDR* transcript did not correlate with patient survival, highlighting a specific association of VDR target genes, but not necessarily *VDR* expression with cancer progression (Supplementary Fig. 11C and D). Comparison of human tumors with high versus low VitD-VDR sign revealed that VitD-VDR sign^high^ cancers displayed specific enrichment for genes and gene signatures of the same immune elements that we found to be required to restrict growth of mouse tumors following increased VitD availability (Supplementary Fig. 11E). This correlation between high VitD-VDR signature and gene signatures of anti-tumor immunity prompted us to further test the value of VitD-VDR sign in predicting responses to immunotherapy. We analyzed >1000 patients treated with immune checkpoint inhibitors (CPI1000^+^ cohort) across seven cancer types using bioinformatic pipelines and standardized clinical criteria, as reported (47). Low expression of VitD-VDR sign and, to a lesser extent, of *VDR*, was associated with resistance to immune checkpoint inhibitors and more rapid disease progression (Fig. 6D, Supplementary Fig. 11F). Overall, these data suggest that, in humans as in mice, lower VitD tissue availability is associated with lower overall immune-mediated control and worse cancer outcome.

Several human epidemiological studies have associated high total (bound and unbound to Gc) and free VitD serum levels with decreased cancer onset and extended patient survival (12–21). However, these studies are inconclusive and limited by relatively small sample sizes. Therefore, we analyzed combined data from the Danish Central Person Registry, the Cancer Registry and the Register of Laboratory Results for Research to include clinical information from a very large cohort of participants (1,496,766 individuals) that lived in Denmark and had at least one vitamin D (25-OHD) serum measurement registered between 2008-2017 (48, 49) (Fig. 6E). Time elapsed since one year following first 25-OHD serum measurement until first diagnosis of cancer was analyzed by a Cox regression model using age as the underlying time scale and adjusting for sex, sample collection time and Charlson’s comorbidity index calculated on the five years before the sample was taken, as described before (50). Skin pigmentation, which can impact VitD_3_ production in response to sun exposure, was not available as a variable but the analysis is unlikely to be affected by differences in ethnicity as the Danish population is highly homogeneous (86% of Danish descent). Further, the relatively northerly latitude of Denmark means that most of the year is “vitamin D winter”; i.e. the period during which cutaneous synthesis of vitamin D_3_ does not occur. Skin cancer was excluded from the study because sun exposure is a major confounder as it contributes to both VitD_3_ synthesis and skin carcinogenesis. (In the previous analysis of cancer outcomes (Fig. 6A, B and D), this confounder is not relevant as we correlated VitD-induced transcripts with outcome of patients that already developed skin cancer.) Notably, and consistent with our preclinical mouse models, we found that a low serum measurement of 25-OHD, indicative of vitamin D deficiency at the time the sample was taken, is associated with increased cancer risk in 6/10 individual cancer cohorts over the following decade. This analysis highlights that low vitamin D serum levels can be a prospective risk factor for cancer development in humans (Fig. 6E).

## Discussion

The interplay between diet, microbiome and the immune system is increasingly recognized as an important component of immunity, including to cancer (51–53). Studies in mice and humans have shown gut commensals to influence anti-cancer immune responses and impact the efficacy of immune checkpoint blockade therapy (54–60). The host factors that allow gut-resident microbes to modulate anti-cancer immune responses remain elusive. Here, we show that increased VitD availability upon genetic deletion of Gc or following vitamin D dietary supplementation alters the gut microbiome to enhance cancer immunity (graphical summary in Supplementary Fig. 12A, B). Specifically, VitD levels appear to regulate the abundance and/or metabolic properties of *Bacteroides fragilis*, an anaerobic Gram-negative bacterium that is part of the normal microbiome of humans and mice. Remarkably, gavage of WT mice with fecal matter from *Gc*^*-/-*^ mice or a non-enterotoxic clinical isolate of *Bacteroides fragilis* was sufficient to confer increased immune-mediated tumor resistance. This did not require antibiotic-mediated conditioning of the recipient mice but necessitated continued availability of dietary vitamin D, demonstrating the dependence of the *Bacteroides fragilis* “niche” on the micronutrient. Our data further indicate that this niche requires the activity of VitD on IECs but further work will be required to understand which VDR-dependent IEC-derived factors are involved and whether they allow for *Bacteroides fragilis* expansion or alter its immunomodulatory activity. With regards to the latter, we do not presently know how *Bacteroides fragilis* acts to boost cancer immunity although our findings suggest that MyD88-dependent receptor signaling and type I IFN production are necessary, as are cDC1-dependent T cell responses. Interestingly, *Bacteroides fragilis* has been previously associated with favorable anti-tumor immune responses following treatment of patients with anti-CTLA-4 whereas gut-resident *Prevotella* species had the opposite effect (55, 61). Further, vitamin D supplementation in healthy human volunteers is associated with a significant increase in intestinal *Bacteroides* species and in the *Bacteroides / Prevotella ratio* (62, 63) and abundance of *Bacteroides fragilis* in human infant fecal samples shows a positive correlation with maternal plasma 25-(OH)D levels (64). Thus, our data suggest a model in which VitD levels in humans, as in mice, modulate the ability of intestinal cells to produce mediators that select for an altered microbiome that includes organisms such as *Bacteroides fragilis*, which potentiate cancer immunity (graphical summary in Supplementary Fig. 12C). Whether this comes at the risk of adverse effects, especially given the ability of *Bacteroides fragilis* to become pathogenic (65), will require further assessment. However, in mice, we do not see evidence for *Bacteroides fragilis*-associated exacerbation of gut inflammation and the bacterium is also reported to protect gut integrity and reduce colorectal cancer induction (66, 67).

In some but not all studies, higher blood levels or increased dietary intake of vitamin D have been correlated with a lower risk of colorectal, breast, prostate and pancreatic tumors (12–21). Our data from nearly 1.5 million individuals, the largest ever such cohort, confirms that a low VitD measurement correlates with increased subsequent risk of cancer incidence. Notably, this may be an underestimate of the true effect of VitD in cancer protection as those individuals who were found to be VitD deficient may have subsequently redressed it with dietary supplements, a factor that is not considered in our analysis. Interestingly, VitD levels at diagnosis of melanoma have been reported to positively correlate with both thinner tumors and better survival (68). As it is exceedingly difficult to control for diet and sunlight exposure, and because a single measurement of VitD may not reflect actual vitamin D availability, we derived a VitD-VDR gene signature as a surrogate of tissue VitD activity. We show that this VitD-VDR gene signature correlates with cancer patient survival, consistent with studies showing that VitD can decrease cancer cell proliferation, promote apoptosis, reduce angiogenesis (7–9), and dampen the pro-tumorigenic activity of cancer-associated fibroblasts (10, 11). Importantly, we further show that the VitD-VDR gene signature correlates with signatures of anti-cancer immunity and with patient responses to immunotherapy. Similarly, VDR expression in melanoma correlates with immune score and increased patient survival, possibly because VDR signals help counteract immunosuppressive Wnt signaling (69). Notably, a recent study reports that greater VitD levels at baseline or after dietary correction correlate with higher responsiveness to immune checkpoint blockade therapy in a cohort of advanced melanoma patients (70). Thus, in humans as in mice, VitD activity appears to potentiate immune responses to cancer.

In sum, here we report that disrupted vitamin D signalling in IECs alters the intestinal microbiome, which in turns impacts immunity to cancer in mice. Further, we show that the vitamin D status of human patients and VitD-VDR signatures within tumors impacts cancer incidence, survival and/or the response to immunotherapy. Further work will be necessary to assess to what extent of overlap between these two findings. Longitudinal studies in humans will help to disentangle the interaction between VitD availability with the microbiome and immunity to cancer, as well as to better assess the effects of vitamin D dietary supplementation.

## Supplementary Material

Supplementary fig. 1

## Data Availability

All data are available in the manuscript or the supplementary materials. Materials and reagents described in this study are either commercially available or available on request from the corresponding author. Shotgun metagenomics data are available through the National Center for Biotechnology Information Sequence Read Archive (NCBI SRA) under BioProject ID PRJNA1077927. Colon RNA sequencing data have been deposited in the Gene Expression Omnibus (GEO) database under the accession number GSE219214.
