# Supplementary material for: Vitamin D regulates microbiome-dependent cancer immunity
Source: Science. Author manuscript; Available in PMC 2024 May 8. (PMC7615937; doi:10.1126/science.adh7954)
Supplement: Supplementary fig. 1 [file EMS195841-supplement-Supplementary_fig__1.pdf]

Science AAAS

Supplementary Materials for

Vitamin D regulates microbiome-dependent cancer immunity

Evangelos Giampazolias *et al*.

Corresponding authors: Evangelos Giampazolias, evangelos.giampazolias@cruk.manchester.ac.uk; Caetano Reis e Sousa, caetano@crick.ac.uk

Science **384**, 428 (2024)
DOI: 10.1126/science.adh7954

The PDF file includes:

Materials and Methods
Figs. S1 to S12
References

Other Supplementary Material for this manuscript includes the following:

Tables S1 to S3
MDAR Reproducibility Checklist

Supplementary Materials

Materials and Methods

Mice

Mice lacking Gc-globulin (*Gc*$^{-/-}$) carrying the Gc$^{tm1.1(KOMP)Vlcg}$ allele on a C57BL/6 background were purchased from KOMP repository. Mice with inserted loxP site upstream of the exon 3 of *Vdr* allele (Vdr^{tm2Ska}) on a C57BL/6 background were purchased from The Australian Phenome Bank at the Australian National University. *Villin*^{Cre} mice were crossed to mice carrying the *Vdr*-floxed allele at the Francis Crick Institute to generate *Vdr*$^{\Delta IEC}$ strain that lacks VDR expression in gut epithelium. *Cgas*$^{-/-}$ mice carrying the Cgas$^{tm1a(EUCOMM)Hmgu/IcsOrl}$ allele on a C57BL/6 background were imported from the MRC Human Immunology Unit, Weatherall Institute of Molecular Medicine, University of Oxford, John Radcliffe Hospital as CRUK import. The above mice, as well as *Rag1*$^{-/-}$, *Batf3*$^{-/-}$, *Tap1*$^{-/-}$, *Ifngr*$^{-/-}$, *Ifnar*$^{-/-}$, *Myd88*$^{-/-}$, *Trif*$^{-/-}$ and wildtype (WT) C57BL/6J were bred and maintained in specific-pathogen free (SPF) conditions in the Biological Research Facility (BRF) at The Francis Crick Institute. WT C57BL/6J (The Francis Crick Institute), C57BL/6J (Charles River) and C57BL/JolaHsd (Envigo) are maintained at the CRUK Manchester Institute. C57BL/6 germ free (GF) mice were bred and maintained at the at the Frederick National Laboratory Gnotobiotics Facility for the National Cancer Institute (NCI), NIH. Mouse genotypes were determined using quantitative PCR with specific probes designed for each gene (Transnetyx, Cordova, TN).

Mice were used at 6 - 21 weeks of age for experiments. For tumor challenge, males and females were used as we did not observe sexual dimorphism (not shown). However, in any one experiment, mice were sex-matched and randomly assigned to treatment or control groups. Mice of different genotypes were littermates and/or separately housed or co-housed in groups for a minimum of 3 weeks before experiments as stated in the figure legends. Animal experiments that were conducted in the UK were performed in accordance with national and institutional guidelines for animal care and were approved by the Francis Crick Institute and CRUK Manchester Institute Biological Resources Facility Strategic Oversight Committee (incorporating the Animal Welfare and Ethical Review Body) and by the Home Office, UK. Animal experiments that were conducted in US were approved by the Institutional Animal Care and Use Committee of National Cancer Institute and were conducted in accordance with the IACUC guidelines and the National Institutes of Health Guide for the Care and Use of Laboratory Animals.

Diets

Mice at the Francis Crick Institute were fed with the commonly used standard chow diet (Teklad Global 18% protein rodent diet, 2018S, Envigo) that contains standard VitD concentration (2 IU/g). Mice at the CRUK Manchester Institute were fed with the commonly used standard chow diet (Teklad Global 18% protein rodent diet, 2018, Inotiv) that contains standard VitD concentration (1.5 IU/g). Mice were fed for 3.5 weeks with modified diet to restrict or enhance VitD levels (*66*). For this, VitD^{Deficient} (VitD 0 IU/g, TD.89123, Teklad custom diet, Envigo), VitD^{High} (VitD 10 IU/g, TD.98234, Teklad custom diet, Envigo) or control VitD^{Standard} (VitD 2 IU/g, TD.110798, Teklad custom diet, Envigo; similar to Teklad Global diet at the Francis Crick Institute) were used. VDR$^{\Delta IEC}$ and littermate control mice were maintained on a VitD$^{Standard+}$ diet supplemented with 2% calcium, 1.25% phosphorus and 20% lactose (VitD 2 IU/g, TD.210810, Teklad custom diet, Envigo) to mitigate the osteomalacia-like effects of abrogating VitD responsiveness in gut epithelium (*35*). For experiments, some VDR$^{\Delta IEC}$ and littermate control mice groups were switched to VitD^{High+} diet (VitD 10 IU/g, TD.210811, Teklad custom diet, Envigo).

Administration of antibiotics

For depletion of microbiota, 0.5 g/L vancomycin (Fisher Scientific, AC296990050), 1 g/L metronidazole (Sigma, M3761) or 1 g/L neomycin (Sigma, N6386) was provided in autoclaved drinking water (changed twice per week) in the presence of sweeteners (Sweet'N Low, 93200) starting 2 weeks before tumor implantation and continuing throughout the study.

Fecal matter transplantations

Freshly or snap frozen (stored at -80°C) stools of similar size were collected from donor mouse strains. The stools were resuspended in PBS (1 fecal pellet/1mL PBS) and 200μl of the dissolved fraction was administered via oral gavage on day -14 and day -12 to mice prior to tumor inoculation on day 0. The recipient mice were deprived of food for 2 hours before and 30 minutes after each fecal transplantation.

Microbial culture and administration to mice

Bacteroides fragilis (NCTC 9343; ATCC 25285) and *Prevotella brevis* GA 33 (ATCC 19188) were procured from the American Type Culture Collection and cultured at 37°C under anaerobic

conditions in a vinyl anaerobic chamber (COY) inflated with a gas mix of approximately 12% carbon dioxide, 86% nitrogen and 2% hydrogen. Prior to the experiment, bacteria were pre-cultured twice, once in modified Gifu anaerobic medium broth (mGAM, HyServe) and then in brain heart infusion broth (BHI, Sigma-Aldrich) supplemented with 5µg/mL hemin and 0.5µg/mL Vitamin K (BBL™ Vitamin K1-Hemin Solution, BD Biosciences). For generating aliquots to be administered to mice, microbes were inoculated into a larger culture volume (500mL and 1 litre supplemented BHI) and grown overnight to an optical density of approximately 2. Bacterial cultures were harvested under anaerobic conditions by centrifuging at 4000 rpm for 20 min and the pellet was washed twice with PBS. Microbial pellets were then adjusted to a cell density of 10^9 bacteria/200µL in PBS containing 25% glycerol, aliquoted in 200 µl aliquots and stored at -80°C. Viability and purity was tested by plating on mGAM agar plates and observing under a microscope.

Microbial species or vehicle (25% glycerol in PBS) were administered via oral gavage on day -14, day -12 and day -10 to mice under the hood, two weeks prior tumor inoculation (day 0). The recipient mice were deprived of food for 2 hours before and 30 minutes after each gavage.

Quantification of bacteria by qPCR

Mouse fecal samples were collected 2 weeks after colonisation with *B. fragilis* or vehicle, snap frozen in liquid nitrogen and stored at -80°C. Genomic DNA was isolated from fecal samples using the QIAamp DNA Stool Mini Kit (Qiagen) following the manufacturer's instructions. Targeted qPCR was performed using the SYBR Green PCR Master Mix (ThermoFisher) on a QuantStudio 7 Flex Real-Time PCR System (ThermoFisher). The following primers (oligo sequence *5'-3'*) and probes were used. *B.fragilis* 16rRNA gene forward primer: TGATTCCGCATGGTTTCATT and reverse primer: CGACCCATAGAGCCTTCATC (*71*). Universal *16S rRNA* forward primer: ACTCCTACGGGAGGCAGCAGT and reverse primer: ATTACCGCGGCTGCTGGC (*72*). *B. fragilis* abundance was calculated relative to universal 16S using $2^{-\Delta\Delta Ct}$ method.

Tumor cell injections

Tumor cell lines (5555 $Braf^{V600E}$ (Caetano Reis e Sousa, The Francis Crick Institute (*73*)), MCA-205 (George Kassiotis, The Francis Crick Institute) and MC-38 (ENH204-FP, The Francis Crick Institute)) were grown in RPMI 1640 (Thermo Scientific-gibco, 31870025) containing 10% FCS (Sigma, F0926-500ML), 2 mM glutamine / 100 units/mL penicillin / 100mg/mL streptomycin

(Thermo Scientific-gibco, 10378016) 50mM 2-mercaptoethanol (Thermo Scientific-gibco, 31350010). All media and media supplements were from Life Technologies except for FCS (Source Bioscience). Tumor cells were dissociated with 0.25% trypsin (Thermo Scientific-gibco, 15090046) and washed three times in PBS. The final cell pellet was resuspended and diluted in endotoxin-free PBS (between 0.2 x 10^6 to 0.5 x 10^6 cells per 100µl) and injected s.c. in the shaved right flank of each recipient mouse. Tumor growth was monitored every 2 to 3 days, and the longest tumor diameter (l) and perpendicular width (w) were measured using digital Vernier calipers; tumor volume was calculated using the formula (*74*): length x width^2 / 2 and is expressed as mm^3. Mice that were poorly injected and did not subsequently develop tumors through the course of the study were excluded from the analysis.

In vivo administration of immune-checkpoint blockade therapy

For immune-checkpoint therapy in vivo, anti-PD1 monoclonal antibody (clone RMP1-14, BioXCell, BE0146) or rat IgG2a isotype control (clone 2A3, BioXCell, BE0089) was administered i.p. at 200µg / 200mL PBS per mouse from day 3 post-tumor cell transplantation, every 3 days up to a maximum of six doses. Alternatively, mice received anti-CTLA-4 monoclonal antibody (clone 9D9, BioXCell, BP0164) or rat IgG2b isotype control (clone MPC-11, BioXCellBE0086) 50µg / 200mL i.p. on days 6, 9 and 12.

In vivo CD8 T cell depletion

For $CD8^+$ T cell depletion, mice received 300µg / 200mL of anti-CD8 (clone 2.43, BioXCell, BE0061) or rat IgG2b isotype control (clone LTF-2, BioXCell, BE0090) i.p. from 3 days prior to inoculation of tumor cells and followed twice per week until the end of the experiment (days: 1, 4, 7, 10, 13, 16, 19, 22).

Analysis of immune populations in the tumor tissue

Tumors were excised at the indicated days after cell transplantation. Tumor weight of individual tumors was determined using a microscale. For subsequent analysis by flow cytometry, tumors were cut into pieces and digested with 200 U/mL collagenase IV (Worthington, LS004188) and 100mg/mL DNase I (Roche, 11284932001) for 60 min at 37^0C. Tissue was passed through a 70 µm cell strainer (Falcon), washed with FACS buffer (PBS with1% FCS and 2mM EDTA) and cells were incubated with Fc block (CD16/32, clone 2.4G2, BD Biosciences, 553141) for 10 min in 4^oC before proceeding with antibody mediated staining.

For the *ex vivo* analysis of T cells, a total of $5x10^6$ isolated cells were treated with 50 ng/mL PMA (Sigma, 16561-29-8), 1 µg/mL ionomycin (Santa Cruz, 56092-82-1) and 1x brefeldin A (eBioscience, 00-4506-51) in complete RPMI medium and incubated for 4 h in Corning 96-well plates. Cell suspensions were stained with LIVE/DEAD Fixable Blue Dead Cell dye (Life Technologies, L34962) according to manufacturer's protocol and subsequently stained with the following antibodies: BV785-CD45.2 (104, Biolegend, 109839, AB_2562604, 1:200 dilution), APC/Cy7 TCRβ (H57-597, Biolegend, 109220, AB_893624, 1:100 dilution), BV605-CD8a (53-6.7, Biolegend, 100744, AB_2562609, 1:200 dilution), PerCP/Cy5.5-CD4 (RM4-5, BD Biosciences, 553052, AB_394587, 1:200 dilution), APC-CD44 (IM7, BD Bioscience, 559250, AB_398661, 1:200 dilution). Cells were fixed and permeabilized using the Fixation/Permeabilisation buffer-Foxp3 Kit (E-Biosciences, 00-5523-00) according to the manufacturer's protocol. For intracellular staining, samples were stained in permeabilization buffer using the following antibodies. FITC-IFNγ (XMG1.2, BD Pharmingen, 554411, AB_395375, 1:100 dilution), Pacific Blue-GZMB (GB11, Biolegend, 515408, AB_2562196, 1:200 dilution), PE-FOXP3 (FJK-16s, E-Bioscience, 12-5773-82, AB_465936, 1:200 dilution). Quantification of total cell numbers by flow cytometry was done using beads (Beckman Coulter). Samples were acquired on a Fortessa X20 B (BD Biosciences). Data were analysed using FlowJo software. After gating on single, live $CD45.2^+$ cells, immune cell populations were defined the following: $CD4^+$ and $CD8^+$ T cells ($CD8a^-CD4^+$ and $CD4^-CD8a^+$ as a proportion of gated live $CD45.2^+TCR\beta^+$), $CD44^+$, $IFN\gamma^+$ and $Foxp3^+$ $CD4^+$ T cells ($CD44^+$, $IFN\gamma^+$ or $Foxp3^+$ as a proportion of gated live $CD45.2^+TCR\beta^+CD8a^-CD4^+$), $CD44^+$, $IFN\gamma^+$ $CD8a^+$ T cells ($CD44^+$ or $IFN\gamma^+$ as a proportion of gated live $CD45.2^+TCR\beta^+CD4^-CD8a^+$).

Analysis of intestinal lamina propria lymphocytes and gut-associated lymphoid tissues

Mouse small intestine, cecum and colon were collected in IMDM (Thermo Scientific-gibco, 21980-082) containing 5% FCS. In the case of the small intestine, Peyer's patches were removed. Mouse intestines were cut longitudinally and carefully washed with PBS to remove fecal particles. Mucus was carefully removed from the intestines by scraping with a scalpel blade. Then, the intestines were vortexed (5 seconds) in ice-cold PBS and the supernatant was discarded. Intestinal tissues were placed in Falcon tubes with pre-warmed IMDM containing 1% FCS, 5 mM EDTA, 1X pen-strep (Thermo Scientific-gibco, 15140-122), 10 mM HEPES (Thermo Scientific-gibco, 11360039), 1X glutamine (Thermo Scientific-gibco, 25030-081) and 2mM DTT (Invitrogen, Y00147) and were incubated at 37°C for 20 min in shaking incubator to remove

epithelial cells. Supernatant were removed and the tissues were washed with cold IMDM/PBS. Small intestines were cut in pieces of 0.5cm length and were placed in Falcon tubes with pre-warmed IMDM medium containing 1% FCS, 1X pen-strep, 10 mM HEPES, 1X glutamine, 1 mg/mL Collagenase VIII (Sigma, C2139-1G) and 33.3 μg/mL DNAse I (Roche, 11284932001) and were incubated at 37°C for 10 min in a shaking incubator. Colon and cecum samples were cut in pieces and were placed in Falcon tubes containing pre-warmed IMDM medium containing 1% FCS, 1X pen-strep, 10 mM HEPES, 1X glutamine, 0.42 mg/mL Liberase (Roche, 05401020001) and 33.3 μg/mL DNAse I (Roche, 11284932001) and were incubated at 37°C for 30 min in a shaking incubator. FACS buffer was added to inhibit enzymatic activity and subsequently the tissue suspensions were passed through 100μm cell strainer on top of a 50-mL tubes using a syringe plunger to force any remaining tissue through the strainer. Cell suspensions were centrifuged at 600g for 10 min, the supernatants were discarded and the cell pellets were resuspended in 40% (v/v) Percoll diluted in IMDM (GE Healthcare,17-0891-01) and were centrifuged at 450g for 8 min. Percoll was aspirated and the remaining cells were restimulated with 50ng/mL PMA, 1 μg/mL ionomycin and 1x brefeldin A in complete RPMI medium and incubated for 4 h in Corning 96-well plates.

For the *ex vivo* analysis of intestinal lamina propria lymphocytes, cell suspensions were stained with LIVE/DEAD Fixable Blue Dead Cell dye (Life Technologies, L34962) according to manufacturer's protocol and subsequently stained with the following antibodies: BV785-CD45.2 (104, Biolegend, 109839, AB_2562604, 1:200 dilution), APC/Cy7 TCRβ (H57-597, Biolegend, 109220, AB_893624, 1:100 dilution), PE-Cy7-TCR-delta (GL3, Biolegend, 118124, AB_11203530, 1:100 dilution), BV605-CD8a (53-6.7, Biolegend, 100744, AB_2562609, 1:200 dilution), CD4 BV785 (RM4-5, Biolegend, 100551, AB_11218992, 1:200 dilution). Cells were fixed and permeabilized using the Fixation/Permeabilisation buffer-Foxp3 Kit (E-Biosciences, 00-5523-00) according to the manufacturer's protocol. For intracellular staining the samples were stained in permeabilization buffer using the following antibodies: PE-FOXP3 (FJK-16s, E-Bioscience, 12-5773-82, AB_465936, 1:200 dilution), Pacific blue-IL17A (TC11-18H10.1, Biolegend, 506918, AB_893545, 1:100 dilution), APC-IL22 (IL22JOP, Invitrogen, 17-7222-82, AB_10597583, 1:100 dilution), FITC-IFNγ (XMG1.2, BD Pharmingen, 554411, AB_395375, 1:100 dilution). Quantification of total cell numbers by flow cytometry was done using beads (Beckman Coulter). Samples were acquired on a Fortessa X20 B (BD Biosciences). Data were analyzed using FlowJo software. After gating on single, live CD45.2$^+$ cells, immune cell populations were defined the following: CD4$^+$ and CD8$^+$ T cells (CD4$^+$ and CD8a$^+$ as a proportion of gated live

CD45.2^{+}TCRdelta- TCRβ^{+}), IFNγ^{+}, IL22^{+} and IL17A^{+} CD4^{+} T cells (IFNγ^{+}, IL22^{+} or IL17A^{+} as a proportion of gated live CD45.2^{+} TCRdelta^{-}TCRβ^{+}CD4^{+}), CD8^{+}IFNγ^{+} T cells (IFNγ^{+} as a proportion of gated live CD45.2^{+} TCRdelta^{-}TCRβ^{+}CD8a^{+}), TCRγδ^{+} T cells (gated on live CD45.2^{+}TCRβ^{-} TCRdelta^{+}), IFNγ^{+}, IL22^{+} and IL17A^{+} TCRγδ^{+}cells (IFNγ^{+}, IL22^{+} or IL17A^{+} as a proportion of gated live CD45.2^{+}TCRβ^{-}TCRdelta^{+}), ILCs (gated on live CD45.2^{+}TCRβ^{-}TCRdelta^{-}), IFNγ^{+}, IL22^{+} and IL17A^{+} ILCs (IFNγ^{+}, IL22^{+} or IL17A^{+} as a proportion of gated live CD45.2^{+} TCRβ^{-}TCRdelta^{-}).

Small intestine and colon samples were prepared using the “Swiss roll” technique, fixed in 4% paraformaldehyde and stained with hematoxylin and eosin. Images were obtained with 10x magnification using Slide Scanner (Zeiss Axio Scan.Z1). Gut-associated lymphoid tissues (GALT) were semi-quantitatively assessed blindly by two board-certified veterinaries using a gut-associated lymphoid tissue scoring system, with a score from 0 – 2 (0 = absent to minimal; 1 = moderate hyperplasia; 2 = marked hyperplasia). Morphologically, a marked GALT score was given when there were abundant and/or single hyperplastic lymphoid follicles containing a wide germinal center with apoptotic and mitotic figures.

Gut permeability assay

Mice were fasted for 4 hours prior to oral administration with 12mg of freshly prepared 4 kDa fluorescein isothiocyanate (FITC)-dextran (Sigma-Aldrich) in PBS. As a positive control of increased gut permeability, WT mice were treated with 2% DSS (MP biomedicals) in drinking water for 5 days prior to FITC-dextran administration. Blood was collected 30min after oral gavage via the tail vein. At 3 hours post injection, mice were culled, and blood was collected from the heart. As a negative control, blood was sampled from a WT mouse that was not injected with FITC-dextran. Blood was centrifuged at 1,000g for 10 min at 4°C, and serum was diluted 1:2 in PBS. 100μl of diluted serum and standards (0-200 mg/mL) were plated in duplicates, and fluorescence was measured using a Tecan spectrophotometer (Tecan Group Ltd.) at an excitation wavelength of 485nm and emission wavelength of 535nm.

Generation of bone marrow chimeras

CD45.1 or CD45.1.2 WT mice were exposed to two doses of 6.6Gy γ-irradiation with a 5-hour interval. The following day the mice received i.v. 2×10^{6} BM cells from CD45.2 WT or *Myd88*$^{-/-}$ mouse donors in 200μL PBS. The mice were placed on acidified drinking water 1 week prior to and 5 weeks following irradiation. Subsequently, reconstituted chimeric mice were returned to non-acidified drinking water for 3 weeks before FT from WT or *Gc*$^{-/-}$ mice (week 8 post-BM

transplantation). Chimeric mice were challenged s.c. with tumors at two weeks post-fecal transplantation as described above.

Mice were bled once by venipuncture of a superficial blood vessel to assess bone marrow chimerism in blood cells before they received the first fecal transplant as follows. Mouse blood was mixed with EDTA (0.5M) and Fc block at 4°C for 10 min and subsequently stained with LIVE/DEAD Fixable Blue Dead Cell dye (Life Technologies, L34962, 1:500 dilution) according to manufacturer's protocol and subsequently stained with the following antibodies: APC-CD45.1 (A20, Invitrogen, 17-0453-82, AB_469398, 1:400 dilution), PE Cy7-CD45.2 (104, Biolegend, 109830, AB_1186103, 1:400 dilution), BV785-CD3 (145-2C11, Biolegend, 100355, AB_2565969, 1:100 dilution), PE-CD19 (1D3, BD Pharmigen, 553786, AB_395050, 1:400 dilution), Pacific Blue-Gr1 (RB6-8C5, Biolegend, 108430, AB_893559, 1:200 dilution), BUV395-CD11b (M1/70, BD Biosciences, 563553, AB_2738276, 1:400 dilution), AF700-MHC-II (I-A/I-E) (M5/114.15.2, E-Bioscience, 56-5321-82, AB_494009, 1:400 dilution), ef780-CD11c (N418, E-Bioscience, 47-0114-82, AB_1548652, 1:100 dilution). Cells were fixed using the red blood lysis solution kit (BD FACS Lysing Solution 10X, 349202) as described in manufacturer's protocol. Samples were acquired on a FACSymphony cell analyzer (BD Biosciences). Quantification of total cell numbers by flow cytometry was done using beads (Beckman Coulter). Samples were acquired on a Fortessa X20 B (BD Biosciences). Data were analyzed using FlowJo software. $CD45.1^{+}$ and $CD45.2^{+}$ cells were defined as a proportion of single, live granulocytes ($CD3^{-}CD19^{-}MHCII^{-}CD11c^{-}CD11b^{+}GR1^{+}$), mononuclear phagocytes ($CD3^{-}CD19^{-}MHCII^{+}CD11c^{+}$), B cells ($CD3^{-}CD19^{+}$) or T cells ($CD19^{-}CD3^{+}$).

DSS-induced colitis model

Colitis was induced by the addition of 2% DSS (Thermo Scientific, J14489.22) to the drinking water for 7 days, and were monitored, daily, for weight loss and disease progression. Moderate severity limits were imposed, with 20% weight loss or two moribund characteristics judged to be the severity threshold. Mice were euthanized at day 7 post-DSS administration or earlier if the symptoms of clinical disease (significant weight loss or diarrhea) became apparent. Colitis progression was assessed using the Disease Activity Index (DAI), as previously described (*75*).

Large intestines were fixed in 4% paraformaldehyde and hematoxylin and eosin staining was performed on 5μm paraffin-embedded cut serial sections (caecum-colon-rectum). Images were obtained at 20x magnification using Slide Scanner (Zeiss Axio Scan.Z1). Colitis severity

was assessed blindly using a histological scoring system as described previously (*75*). In addition, spleen mass and colon length were measured to assess the severity of intestinal inflammation.

Western blot

Dissected tissues from VDR^{WT} and $VDR^{\Delta IEC}$ were snap frozen in liquid nitrogen and stored at -80°C until required. 20-40 mg of each tissue was homogenized in lysis buffer (Cell Lysis Buffer (10X, Cell Signaling Technology), cOmplete Protease Inhibitor Cocktail (Sigma Aldrich), Halt Protease & Phosphatase Inhibitor Cocktail (Thermo Fisher Scientific), PMSF Protease Inhibitor (Thermo Fisher Scientific)) using QIAGEN TissueLyser LT (50 Hz, 10 min). Protein suspensions were centrifuged twice (1000 x *g,* 2 min, and 10,000 x g, 10 min) and supernatants harvested. Protein concentrations were determined using Pierce BCA Protein Assay Kit (Thermo Fisher Scientific) per the manufacturer's instructions. Equal quantities of protein from VDR^{WT} and $VDR^{\Delta IEC}$ tissues (25 µg small and large intestine, 50 µg kidney, and 100 µg spleen) were denatured at 75°C for 10 min in 50 mM DTT and NuPage LDS Loading Dye (4X, Thermo Fisher Scientific). Samples were run on 7.5 % Mini-PROTEAN TGX Pre-cast Protein Gels in Tris/Glycine/SDS buffer alongside Precision Plus Protein Dual Color Standards (all Bio-Rad) for 60 min at 100 V. Protein transfer to 0.2 µm PVDF membrane was performed using the Trans-Blot Turbo Transfer System (Bio-Rad) per the manufacturer's instructions. Membrane was blocked in 5 % milk in Tris-buffered saline with 0.1 % Tween (TBST) for 1 h at room temperature (RT) before addition of Vitamin D3 Receptor (D2K6W) Rabbit mAb (1:2000, Cell Signaling Technology) in 5 % milk in TBST overnight at 4°C. After three washes in TBST, membrane was incubated with GAPDH (14C10) Rabbit mAb (1:5000, Cell Signaling Technology) in 5 % milk in TBST for 1 h at RT. After three washes, VDR and GAPDH were detected using secondary anti-rabbit IgG-HRP (7074S) (1:2000, Cell Signaling Technology) in 5 % milk in TBST for 1 h at RT. Membrane was incubated with SuperSignal West Pico PLUS chemiluminescent signal (Thermo Fisher Scientific) and imaged using an Amersham ImageQuant 800.

Quantification of Vitamin D_3 metabolites in serum using supercritical fluid chromatography-Mass spectrometry (SFC-MS)

Supercritical fluid chromatography-mass spectrometry (SFC-MS) was used for the measurement of $VitD_3$ metabolites in mouse serum based on the elution time of isotope-labelled $VitD_3$ internal standards (supplemental table 3). Serum samples (200µL) were spiked with 20µL of internal

standard 125 ng/mL in methanol 25-HydroxyvitaminD$_3$(6,19,19-d3) (Sigma, 705888) and incubated for 10 min on ice. Acetonitrile (500μL, HPLC grade) was added to samples and vortexed for 1 min to precipitate protein and dissociate vitamin D metabolites from vitamin D binding proteins. Samples were centrifuged at 12,700 rpm for 10 min at 4°C and the supernatant (SN1) was transferred to a clean Eppendorf 1.5 mL tube. The pellet was re-extracted with 500μL of acetonitrile, vortexed and centrifuged (12,700 rpm, 10 min at 4°C). The supernatant (SN2) was transferred and combined with SN1. Combined SN extracts were dried under a gentle stream of nitrogen. Ethyl acetate (200μL) and ultrapure water (100μL) were added to the dry samples, vortexed for 1 minute, and centrifuged (12,700 rpm, 5 min at 4°C). The upper organic layer (OL1) was transferred to a new Eppendorf 1.5 mL tube while the aqueous layer was re-extracted by adding 200μL ethyl acetate. Samples were vortexed, centrifuged (12,700 rpm, 5 min at 4°C) and the organic layer (OL2) was combined with OL1. The OL fractions were dried under a gentle stream of nitrogen and reconstituted in 50μL of methanol. Samples were stored at -20 °C (overnight) or -80°C (short term) until further SFC-MS analysis.

The SFC separation of 1 μL injection volume was performed using a Waters Acquity UPC2 system, coupled to a Waters Xevo TQ-S micro QqQ MS detector (Waters Corporation, USA), with a CSH fluoro-phenyl column (50 x 2.1 mm, 1.7 μm) set at 50 °C and the ABPR (automated back pressure regulator) at 1500 psi. The mobile phase was CO_2 (A) and methanol (B) flowing at a 1 mL/min rate. The elution started at 2% B, maintained for 0.95 min, followed by an increased to 5% at minute 1.08 and maintained until minute 2.67. Methanol was increased to 10%, maintained from minute 3.65 to 5.1, and finally returned to 2% at minute 5.61, with a re-equilibration of 59 seconds. Methanol with 0.2% ammonium formate at 0.25 mL/min flow rate was used as make-up solvent to enhance the ionization process for vitamin D3 metabolites before entering the MS. Multiple reaction monitoring (MRM) was used for vitamin D$_3$ metabolite detection, using positive electrospray ionization mode (ES+) with MRM channels, parent and daughter ions, cone voltage and collision energies shown in Supplemental Table 3. Capillary voltage was set to 3.5 kV, desolvation flow to 1000 L/Hr and desolvation temperature to 500 °C. Data were recorded using MassLynx V4.2 and analysed using TargetLynx XS V4.2 software.

The developed method was subject to validation by assessing the following parameters: selectivity, linearity, limits of detection (LOD) and quantification (LOQ), recovery, accuracy and precision. Vitamin D depleted charcoal stripped serum (Golden West Biologicals Inc.), certified for vitamin D UHPLC-MS/MS applications was used as control matrix for validation experiments. Linearity was evaluated from 2.5 to 1000 ng/mL (final concentration in serum from 0.625 to 250

ng/mL). The LOD and LOQ were set using a signal-to-noise ratio of 3 and 10, respectively. Three controls, at low (10 ng/mL), medium (50 ng/mL) and high (500 ng/mL) concentrations, were used to assess recovery, accuracy and precision in triplicate on four non-consecutive days. Samples with poor recovery of the internal standards were excluded from the analysis.

Shallow shotgun metagenomic sequencing of fecal matter

Transnetyx Microbiome kits containing barcoded sample collection tubes were provided by Transnetyx (Cordova, TN, USA). Mouse fecal samples were placed in individual tubes containing DNA stabilization buffer to ensure reproducibility, stability, and traceability, and shipped for DNA extraction, library preparation, and sequencing by Transnetyx (Cordova, TN USA). DNA extraction was optimized and fully automated using a robust process for reproducible extraction of inhibitor-free, high molecular weight genomic DNA that captures the true microbial diversity of stool samples. DNA extraction is performed using the Qiagen DNeasy 96 PowerSoil Pro QIAcube HT extraction kit and protocol. After DNA extraction and quality control (QC), genomic DNA was converted into sequencing libraries using a method optimized for minimal bias. Unique dual indexed (UDI) adapters were used to ensure that reads and/or organisms are not misassigned. Library preparation is performed using the KAPA HyperPlus library preparation protocol. After QC, the libraries were sequenced using the Illumina NovaSeq instrument and the shotgun sequencing method (a depth of 2 million 2x150 bp read pairs), which enables species and strain level taxonomic resolution. FASTA/Q files are available upon request.

Analysis of shotgun sequencing data was performed using JAMS_BW package (v1.8.0) (*76*) with its Jan 2022 database publicly available in GitHub as described (*77*, *78*). Taxonomy was expressed as the last known taxa (LKT), or the taxonomically lowest unambiguous classification determined for each query sequence, using Kraken's confidence scoring threshold of 5×10^{-6} (using the --confidence parameter); p = phylum, c = class, o = order, f = family, g = genus, s = species. Relative abundance for each LKT within each sample was obtained by dividing the number of base pairs covering all contigs and unassembled reads pertaining to that LKT by the total number of non-host base pairs sequenced for that sample. Relative abundances were expressed as PPM. Gene product annotations were obtained using Prokka. Low abundant features were filtered if they were not >50 PPM (taxonomy) or >5 PPM (gene product) in at least 5% of samples. For taxonomy, information must come from >70% contigs in at least 50% of samples and taxon genome completeness must be >5% in at least 5% of samples.

For each feature obtained from the shotgun metagenomic sequencing, p values were calculated using the Mann–Whitney–Wilcoxon U test on PPM relative abundance for that feature in samples for the following pairwise comparisons: WT + $VitD_3^{Standard}$ vs. *Gc*$^{-/-}$ + $VitD_3^{Standard}$ performed for two independent experiments; WT + $VitD_3^{High}$ vs. *Gc*$^{-/-}$ + $VitD_3^{High}$; WT + $VitD_3^{Standard}$ vs. WT + $VitD_3^{High}$. A meta-analysis method was used to determine common features associated with changes in the tumor growth phenotype. Fold change was calculated using median within each group and then averaged (mean) across groups when indicated as Combined. Features that agreed in directionality of differences across individual comparisons were considered for the meta-analysis. The combined p value (cp) for meta-analysis of across-group comparisons was calculated using Fishers p value. For each feature type, the cut-offs for the meta-analysis were as followed: p-value< 0.2, CP< 0.1, FDR<0.15.

RNA sequencing of colon

Mouse colon was harvested and homogenized in Precellys lysing kit (Bertin Instruments, P0000917-LYSK0-A) and Precellys 24 tissue homogenizer (Bertin Instruments, P000062-PEVO0-A). RNA was extracted from the homogenized tissue using the RNeasy Mini Kit (QiaGEN, 74106).

Biological replicate libraries were prepared using the polyA KAPA mRNA HyperPrep Kit and sequenced on Illumina HiSeq 4000 platform, generating ~53 million 75bp single end reads per sample. The RSEM package (*79*) (version 1.3.30) in conjunction with the STAR alignment algorithm (*79*) (version 2.5.2a) was used for the mapping and subsequent gene-level counting of the sequenced reads with respect to Ensembl mouse GRCm.38.89 version transcriptome. Normalization of raw count data and differential expression analysis was performed with the DESeq2 package[70] (version 1.24.1) within the R programming environment (version 3.6.0).

Vitamin D and cancer risk epidemiological study in Danish population

The aim of the analysis was to estimate hazard ratio of cancer by site given vitamin D measurement measured at least one year before cancer diagnosis using data from the Danish health registries covering the period 2008-2017. The described analyses combine data from the Central Person Registry, the Cancer Registry and the Register of Laboratory Results for Research (*48*, *49*). The study is based on data from the Danish nationwide registers (https://sundhedsdatastyrelsen.dk [sundhedsdatastyrelsen.dk]), which does not require ethical permission. The Danish Act on Processing of Personal Data has been followed.

Central Person Registry: Personal identifiers on everybody in Denmark. Data currently available from 2.4.1968 until 31.12.2018. Cancer Registry: Data on all cancers diagnosed in Denmark, currently available from 1.1.1943 until 31.12.2017. Register of Laboratory Results for Research (RLRR). The RLRR covers all regions of Denmark and includes the results of biochemistry and immunology tests taken at hospitals and general practitioners with data from 2008 and onwards. The Cancer Registry contains data on all cancers diagnosed in Denmark from the 1940s and onwards. The study population consisted of all individuals living in Denmark between 2008 and 2017 with at least one serum measurement of 25-OHD (NPU10267 (P-25-Hydroxy-Vitamin D(D_2+D_3)) who were living in Denmark in the period from one year before the first sample and had never had a cancer diagnosis at the time of the first sample. Exposure was defined based on the first 25-OHD measurement in RLRR. Follow-up started one year after sample was taken since vitamin D might be measured as part of testing connected to diagnosis. People who died, emigrated, or had cancer in the first year after the first sample were excluded. The first vitamin 25-OHD measurement in the Register of Laboratory Results for Research starting follow-up one year after sample was taken since vitamin D might be measured as part of testing connected to diagnosis. A total of 1,496,766 individuals had at least one 25-OHD measurement with a usable, non-missing result during 2008 and 2016 and satisfied the inclusion criteria. Among these, 38,905 individuals had measurements above 125 nmol/L that are considered too high and might cause health problems. Because of this restriction individuals with 25-OHD values above 125 nmol/L are excluded resulting in a final study population of 1,457,861 individuals. Median length of follow-up (starting one year after sample was taken) was 2.9 years (IQR: 1.3-5.1). Characteristics of individuals including sex, age, year of sample collection and Charlson's comorbidity index are available upon request.

Bioinformatic analysis of human tissues and cancer patient data

Normalized read counts for *VDR* expression were downloaded from the Genotype-Tissue Expression (GTEx) resource Biobank [https://gtexportal.org]. RSEM normalised gene expression data was downloaded from TCGA [https://gdac.broadinstitute.org/] for 21 cancer datasets. All analysis were run using R version 3.6.1. Healthy samples and replicates samples were removed. Only one tumor sample per patient was taken forward for downstream analyses. A Vitamin D-VDR gene signature consisting of 237 genes was generated using primary VDR target genes that have been identified using ChIP-sequencing in primary human cells following in vitro stimulation with 1,25-$(OH)_2D$ (*11, 42–46*) (Supplemental Table 2). Where fold change of gene expression was

reported, genes were selected based on log_2Fold Change>1.5. The human cell types used include the monocytic THP1 cell line, blood mononuclear cells, fibroblasts, cancer-associated fibroblasts, hepatic stellate cells, colorectal cancer cells and lymphoblasts. From these 237 genes, 223 were present in the TCGA datasets (Supplemental Table 2) and patients were ranked using the sum of the 223 gene Vitamin D-VDR signature. Patients in the top and bottom quartiles were used to draw the survival plots. To assess the effect of age, sex and stage on the survival, forest plots were generated with the full data using the 'survminer' package (https://CRAN.R-project.org/package=survminer). In the CP1000$^+$ cohort (*47*) 230 genes (Supplemental Table 2) were presented and their sum expression was used as the Vitamin D-VDR signature and its expression was compared across groups that exhibited differential response to immune checkpoint inhibitors (CPI). Based on the advice from leading oncologists in Memorial Sloan Kettering Cancer Centre (MSKCC), "Exceptional response" to CPI was defined as complete (CR) or partial response (PR) with more or equal to 24 months survival, whereas "Rapid Progressor" was defined as disease progression (PD) with overall survival (OS) of less than 3 months.

Quantification and statistical analysis

Most statistical analyses were performed using GraphPad Prism software (GraphPad). Statistical tests are listed in the associated figure legends. Statistical significance between two groups was determined using an unpaired two-tailed Student's t test with Welch Correction. Statistical analyses for two or more groups were done by one- or two-way ANOVA followed by Bonferroni multiple-comparison post hoc correction. One-way ANOVA was used to compare average means of two or more groups obtained in a single time point. Two-way ANOVA was used to compare average means of two or more groups over time (tumor growth profile, mouse weight and disease activity curves) or average median of multiple groups of different genotypes (colon length, spleen mass, alpha diversity). The Log-rank "Mantel-Cox" (for two group comparison) and "for trend' (for multiple group comparison) tests were used to determine statistical significance for incidence of tumor rejection or overall survival in mice and cancer patient data from TCGA. In the gene-enrichment analysis using genes were ranked by the Wald's test false discovery rate (FDR)-adjusted p were calculated. PERMANOVA was used to compare taxa diversity between two groups. Differentially expressed genes were defined as those showing statistically significant differences between genotype and treatment groups (FDR <0.05). Gene lists ranked by the Wald statistic were used to look for pathway and biological process enrichment using the Broad's GSEA software (version 2.1.0) with gene sets from

MSigDB (*80*) (version 6). Frequency of tumour stages were compared between groups using Chi-squared test. Time from one year after first vitamin D_2+D_3 sample until first diagnosis of cancer was analyzed by a Cox regression model using age as the underlying time scale and adjusting for sex, age at sample and Charlson's comorbidity index calculated based on the five years before the sample was taken (Quan 2005). Data are shown as mean±SD or mean±SEM or median or hazard ratios with 95% confidence intervals as indicated in the figure legends. Significance was assumed with *p < 0.05, **p < 0.01, ***p < 0.001, ****p < 0.0001.

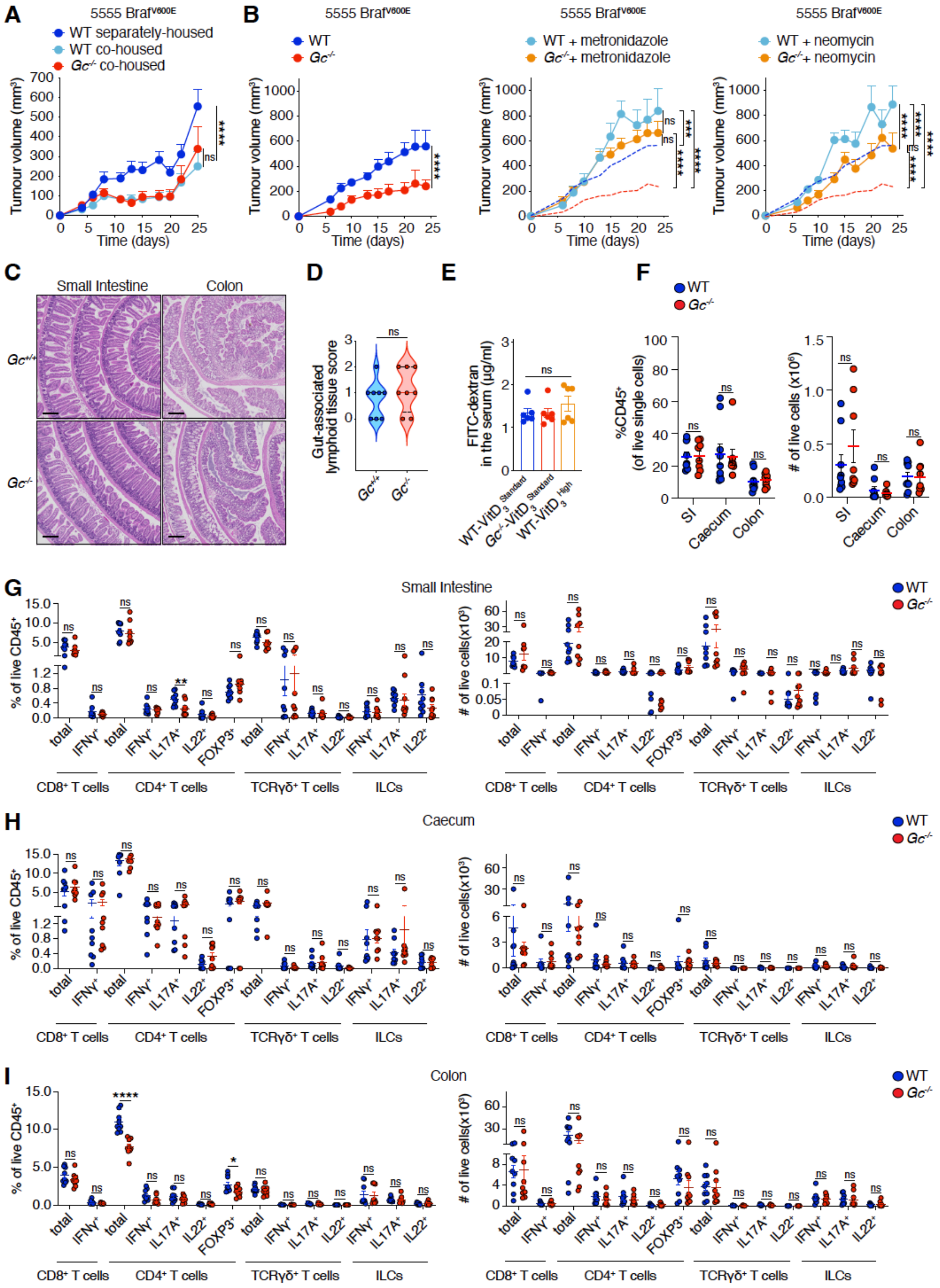

Supplementary Fig. 1. *Gc*$^{-/-}$ mice exhibit microbiome-dependent tumor resistance in absence of intestinal barrier disruption or inflammation. (**A-B**) Growth profile of 0.2 x 10^6

5555 $Braf^{V600E}$ cancer cells implanted into: (**A**) Separately housed WT C57BL/6J (n=12) and co-housed WT (n=12) and $Gc^{-/-}$ (n=12) groups of mice. Data are representative of two independent experiments. (**B**) Separately housed groups of WT or $Gc^{-/-}$ mice that received or not metronidazole (1 g/L) or neomycin (1 g/L) in the drinking water starting from 2 weeks prior to tumor inoculation. WT (n=10), $Gc^{-/-}$ (n=10), WT + metronidazole (n=9), $Gc^{-/-}$ + metronidazole (n=10), WT + neomycin (n=11), $Gc^{-/-}$ + neomycin (n=10). (**C-D**) Separately housed groups of $Gc^{-/-}$ (n=8) and $Gc^{+/+}$ (n=8) littermate control mice inoculated with 0.2 x 10^6 5555 $Braf^{V600E}$ cancer cells. (**C**) Representative H&E images of small intestine and colon samples (scale bar, 250 μm). (**D**) Quantification of immune infiltrates and gut-associated lymphoid tissues in histology samples from (C). (**E**) WT or $Gc^{-/-}$ mice (n=6 mice per group) fed with $VitD_3$ standard (2 IU/g) or $VitD_3$ high (10 IU/g) diet for 3.5 weeks were gavaged with FITC-dextran and fluorescence was measured in plasma 3h later. (**F-I**) Quantification of (**F**) total or (**G-I**) the indicated immune cell populations in (**G**) small intestine, (**H**) caecum and (**I**) colon of separately housed groups of WT C57BL/6J (n=9) or $Gc^{-/-}$ (n=9) mice. Data in (A-B) are presented as tumor volume (mm^3) + SEM and are from one experiment. Data in (D) are presented as median of lymphoid tissue score and are from one experiment. Data in (E) are presented as mean ± SEM from two independent experiments. Data in (F-I) are presented as mean frequency (left) or numbers of cells (right) from three independent experiments. Tumor growth profiles (A-B) were compared using Bonferroni-corrected two-way ANOVA. Groups in (E) were compared using Bonferroni-corrected one-way ANOVA. Groups in (D, F-I) were compared using two-tailed unpaired t test with Welch's correction. *$p<0.05$, **$p<0.01$, ****$p< 0.0001$; ns, not significant.

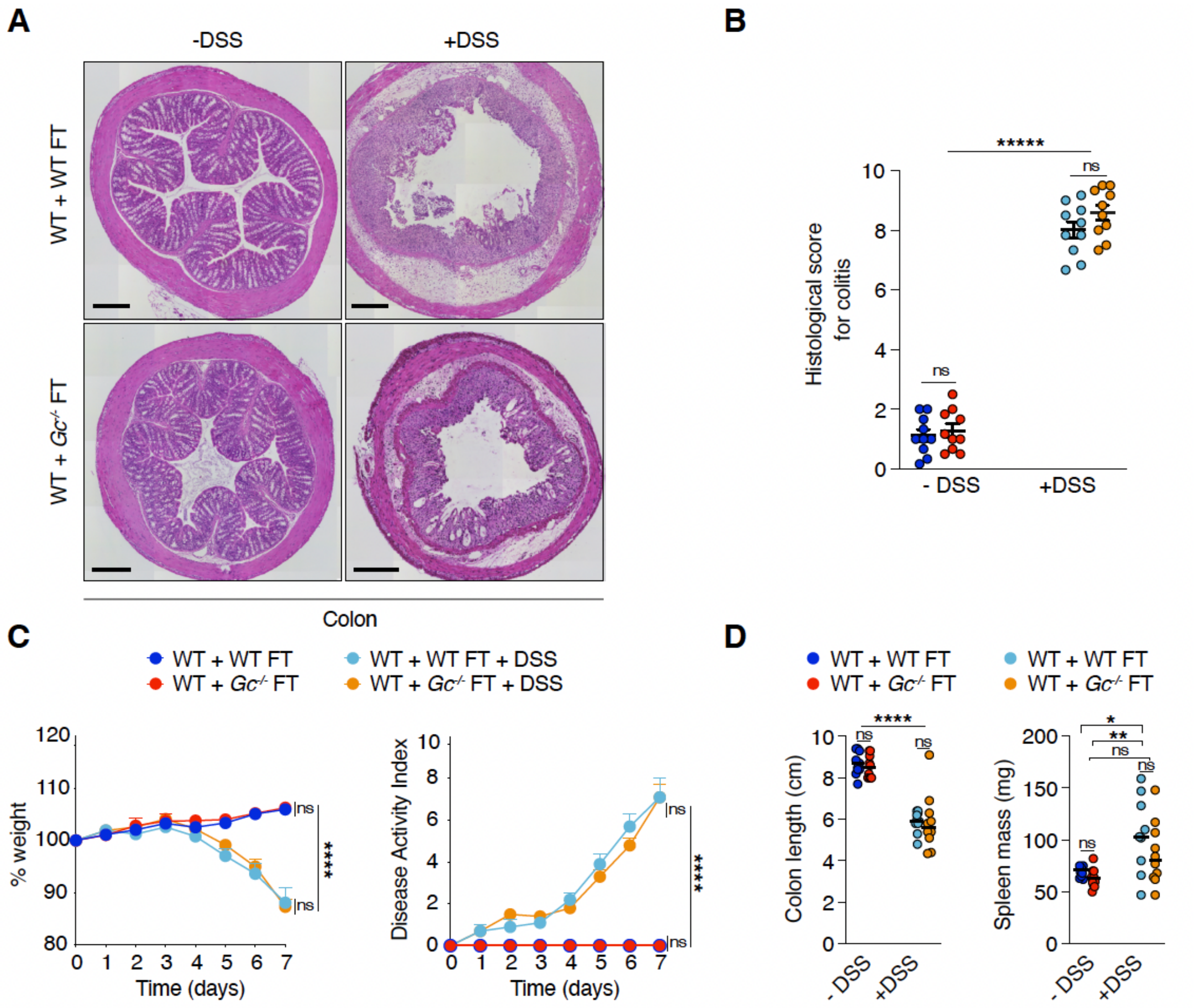

Supplementary Fig. 2. Gc deficiency does not alter sensitivity of mice to DSS-induced colitis. (**A-D**) Separately housed WT C57BL/6J mouse groups (n=10 per group) that received FT from WT C57BL/6J or *Gc*$^{-/-}$ donors (on days -14 and -12) prior to administration or not of 2% DSS (day 0) in the drinking water. DSS-induced colitis was measured after 7 days of switching to DSS water. (**A**) Representative H&E images of transversely cut colon segments (scale bar, 250 µm). (**B**) Histopathological scoring of colitis severity. (**C**) Quantification of normalised colon mass (left) and disease activity index (right) measurement over time following DSS administration. (**D**) Quantification of colon length (cm) (left) and spleen mass (mg) (right) over time following DSS administration. Data in (B-C) are presented as mean ± SEM, data in (D) are presented as median and are from one experiment. Data in (B-D) were compared using Bonferroni-corrected two-way ANOVA. *p<0.05, **p<0.01, ****p< 0.0001; ns, not significant.

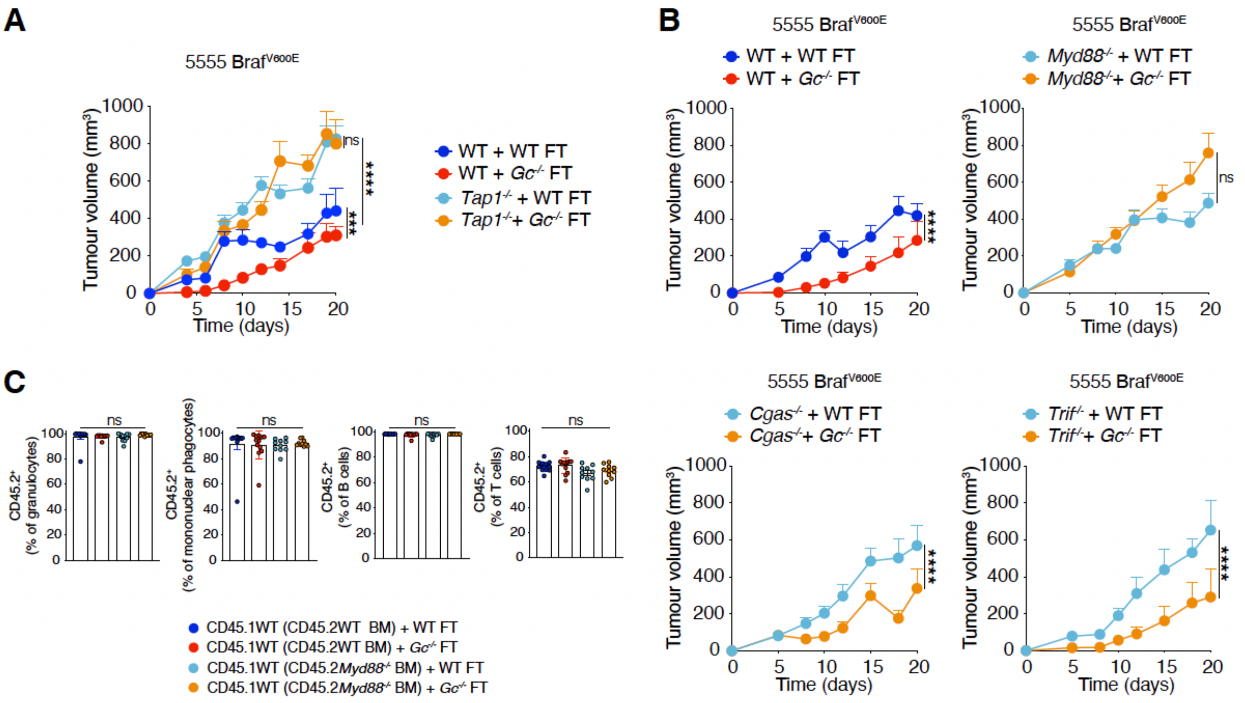

Supplementary Fig. 3. Fecal transplants from *Gc*$^{-/-}$ mice augment tumor control dependent on CD8$^+$ T cells and MyD88. (**A-B**) Growth profile of 0.2 x 10^6 5555 Braf^{V600E} cancer cells implanted into the indicated separately housed groups of mice that received (on days -14 and -12 prior to tumor inoculation) FT from WT C57BL/6J or *Gc*$^{-/-}$ donors. (**A**) WT + WT FT (n=10), WT + *Gc*$^{-/-}$ FT (n=10), *Tap1*$^{-/-}$ + WT FT (n=10), *Tap1*$^{-/-}$ + *Gc*$^{-/-}$ FT (n=10). (**B**) WT + WT FT (n=9), WT + *Gc*$^{-/-}$ FT (n=10), *Cgas*$^{-/-}$ + WT FT (n=11), *Cgas*$^{-/-}$ + *Gc*$^{-/-}$ FT (n=11), *Trif*$^{-/-}$ + WT FT (n=10), *Trif*$^{-/-}$ + *Gc*$^{-/-}$ FT (n=11), *Myd88*$^{-/-}$ + WT FT (n=9), *Myd88*$^{-/-}$ + *Gc*$^{-/-}$ FT (n=10). (**C**) Quantification of the indicated immune populations in the blood of irradiated CD45.1 WT mice whose bone marrow (BM) was reconstituted using BM from CD45.2 WT or *Myd88*$^{-/-}$ donors. WT (WT BM) + WT FT (n=11), WT (WT BM) + *Gc*$^{-/-}$ FT (n=12), WT (*Myd88*$^{-/-}$ BM) + WT FT (n=10), WT (*Myd88*$^{-/-}$ BM) *Gc*$^{-/-}$ FT (n=10). Data in (A-B) are presented as tumor volume (mm^3) + SEM. Data in (A) are representative of two independent experiments. Data in (B) are from one experiment apart from the growth profiles of WT and *Myd88*$^{-/-}$ mice that are representative of two independent experiments. Data in (C) are presented as mean frequency ± SEM and are representative of two experiments. Data in (A-C) were compared using Bonferroni-corrected two-way ANOVA. ***$p< 0.001$, ****$p< 0.0001$; ns, not significant.

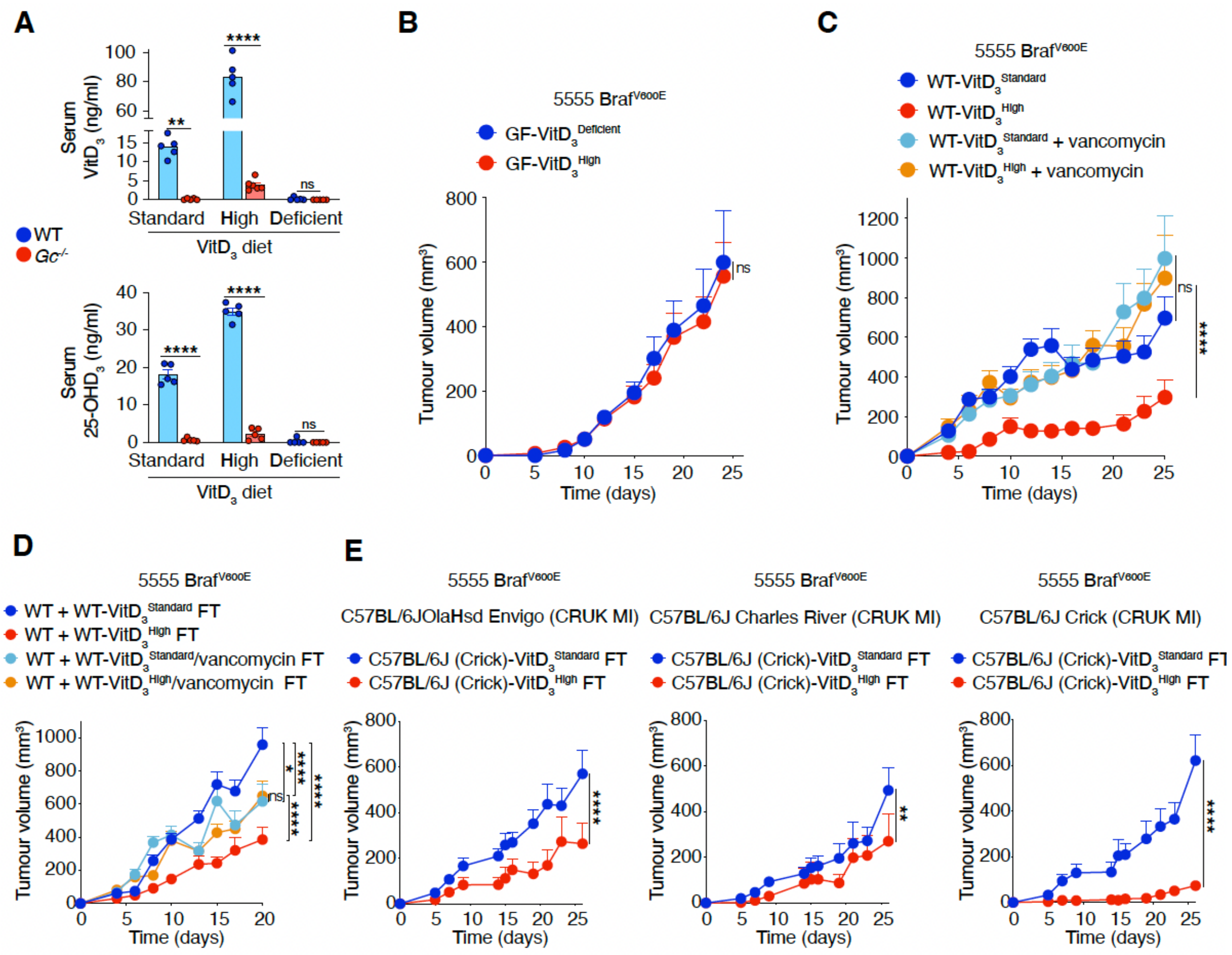

Supplementary Fig. 4. Mice with increased VitD dietary supplementation develop microbiome-dependent transmissible tumor resistance. (**A**) Quantification of VitD$_3$ metabolites in the serum of separately housed groups of WT C57BL/6J (n=5 per group) or *Gc*$^{-/-}$ (n=5 per group) mice that were fed with VitD$_3$ standard (2 IU/g), deficient (0 IU/g) or high (10 IU/g) diet starting from 3.5 weeks. (**B-E**) Growth profile of 0.2 x 10^6 5555 Braf^{V600E} cancer cells implanted into: **B**) Separately housed groups of C57BL/6J germ-free (GF) mice (n=10 per group) that were fed with VitD$_3$ deficient (2 IU/g), or high (10 IU/g) diet starting from 3.5 weeks prior to tumor inoculation. (**C**) Separately housed groups of C57BL/6J WT mice (n=10 per group) that were fed with VitD$_3$ standard (2 IU/g), or high (10 IU/g) diet starting from 3.5 weeks prior to tumor inoculation and received or not vancomycin (0.5 g/L) in the drinking water starting from 2 weeks prior to tumor inoculation. (**D**) C57BL/6J WT mice (n=10 per group) that received FT (on day -14 and -12) from donors treated as in (C). (**E**) C57BL/6 mice were purchased from two different commercial vendors [C57BL/6JOlaHsd2 Envigo (n=10 per group], [C57BL/6J Charles River (n=10 vs. n=9)] or were procured from the Crick [C57BL/6J Crick (n=8 vs. n=8)] and separately

housed at CRUK Manchester Institute (CRUK MI). All groups received FT (on day -14 and -12) from C57BL/6J Crick donors that were fed with $VitD_3$ standard (2 IU/g) or high (10 IU/g) diet. Data in (A) are presented as mean concentration ± SEM and are from one experiment. Data in (B-E) are presented mean tumor volume (mm^3) + SEM and are from one experiment. Data in (A-E) were compared using Bonferroni-corrected two-way ANOVA. *$p<0.05$, **$p<0.01$, ****$p<0.0001$; ns, not significant.

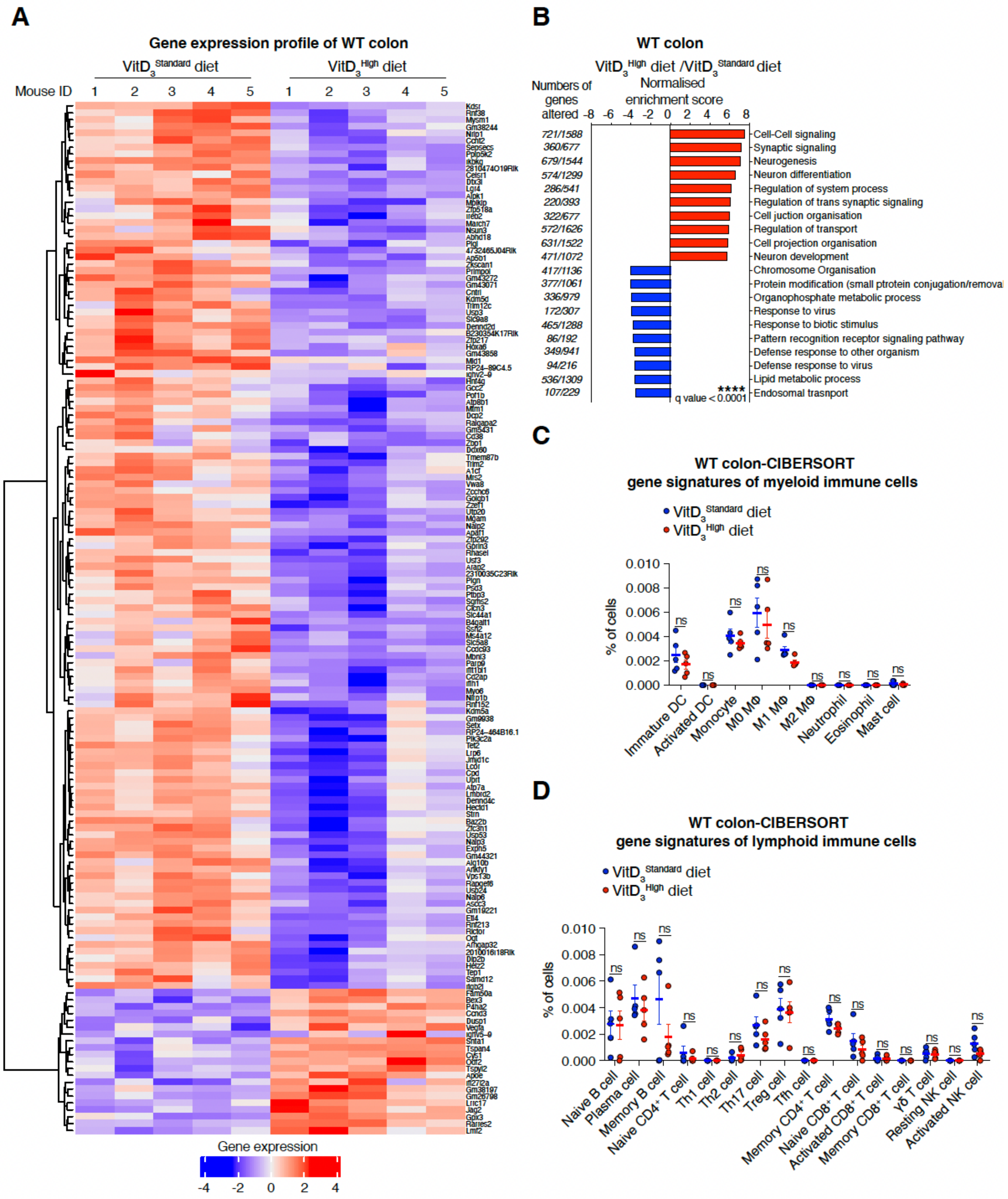

Supplementary Fig. 5. RNA sequencing of the colon of WT mice fed with standard or high VitD diet. (**A-D**) RNA sequencing of colons from separately housed groups of WT C57BL/6J mice (n=5 per group) that were fed with $VitD_3$ standard (2 IU/g) or high (10 IU/g) diet for 3.5 weeks. (**A**) Gene expression profile (heatmap) of individual colon samples. (**B**) Gene ontology

biological process (GOBP) analysis in the high ($VitD_3^{High}$ diet) comparing to the ($VitD_3^{Low}$ diet) group of colon samples mapped to Gene Ontology Biological Process (GOBP) database. (**C-D**) Proportion of CIBERSORT-gene signatures of (**C**) myeloid and (**D**) lymphoid immune populations within individual colon samples. Gene expression in (A) is presented as normalized read counts across samples for each gene (z-scores). In (B) all genes were ranked by the Wald's test and FDR-adjusted p values (q values) were calculated. In (A-B), data are normalized gene expression in (A) and normalized enrichment score in (B) with positive values in red, negative values in blue. In (C-D) data are presented as frequency mean ± SEM of gene signatures within a sample. In (C-D) frequency means of gene signatures between groups were compared using Bonferroni-corrected two-way ANOVA. ****$p < 0.0001$; ns, not significant. Data in (A-D) are from one experiment.

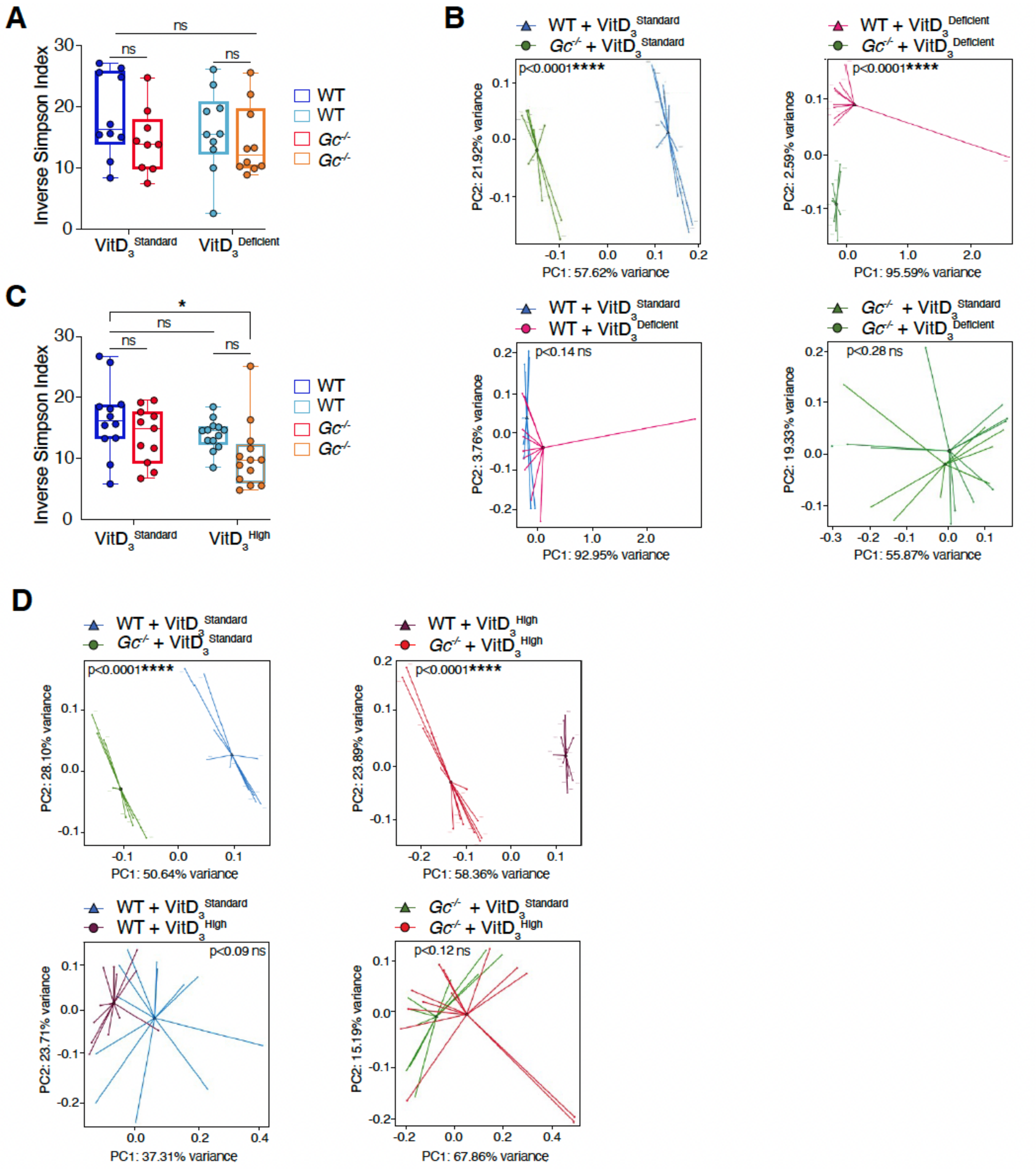

Supplementary Fig. 6. Bacterial taxonomic diversity in the fecal matter of mice with high or low VitD availability. (**A-D**) Sequenced bacterial taxa diversity (**A, C**) within (alpha diversity, inverse Simpson index) and (**B, D**) across (beta diversity, principal component analysis) fecal samples from separately housed groups of WT C57BL/6J or *Gc*$^{-/-}$ mice that were fed with VitD$_3$ standard (2 IU/g) or deficient (0 IU/g) **(A, B)** or VitD$_3$ standard (2 IU/g) or high (10 IU/g) **(C, D)** diet

for 3.5 weeks. (A, B) WT + $VitD_3^{Standard}$ (n=10), *Gc*$^{-/-}$ + $VitD_3^{Standard}$ (n=9), WT + $VitD_3^{Deficient}$ (n=10), *Gc*$^{-/-}$ + $VitD_3^{Deficient}$ (n=10). (C-D) WT + $VitD_3^{Standard}$ (n=12), *Gc*$^{-/-}$ + $VitD_3^{Standard}$ (n=11), WT + $VitD_3^{High}$ (n=13), *Gc*$^{-/-}$ + $VitD_3^{High}$ (n=13). Data in (A, C) are presented as inverse Simpson index box-and-whisker plots with median, 25th and 75th percentiles represented by the box and min/max by the whiskers and are from one experiment. Data in (B, D) are presented as projected data of variances of two individual principal components (PC1 and PC2). Inverse Simpson indexes (A, C) were compared using Bonferroni-corrected two-way ANOVA. Taxa composition between groups in (B, D) were compared using PERMANOVA. *$p<0.05$, **$p<0.01$, ***$p<0.001$, ****$p<0.0001$; ns, not significant.

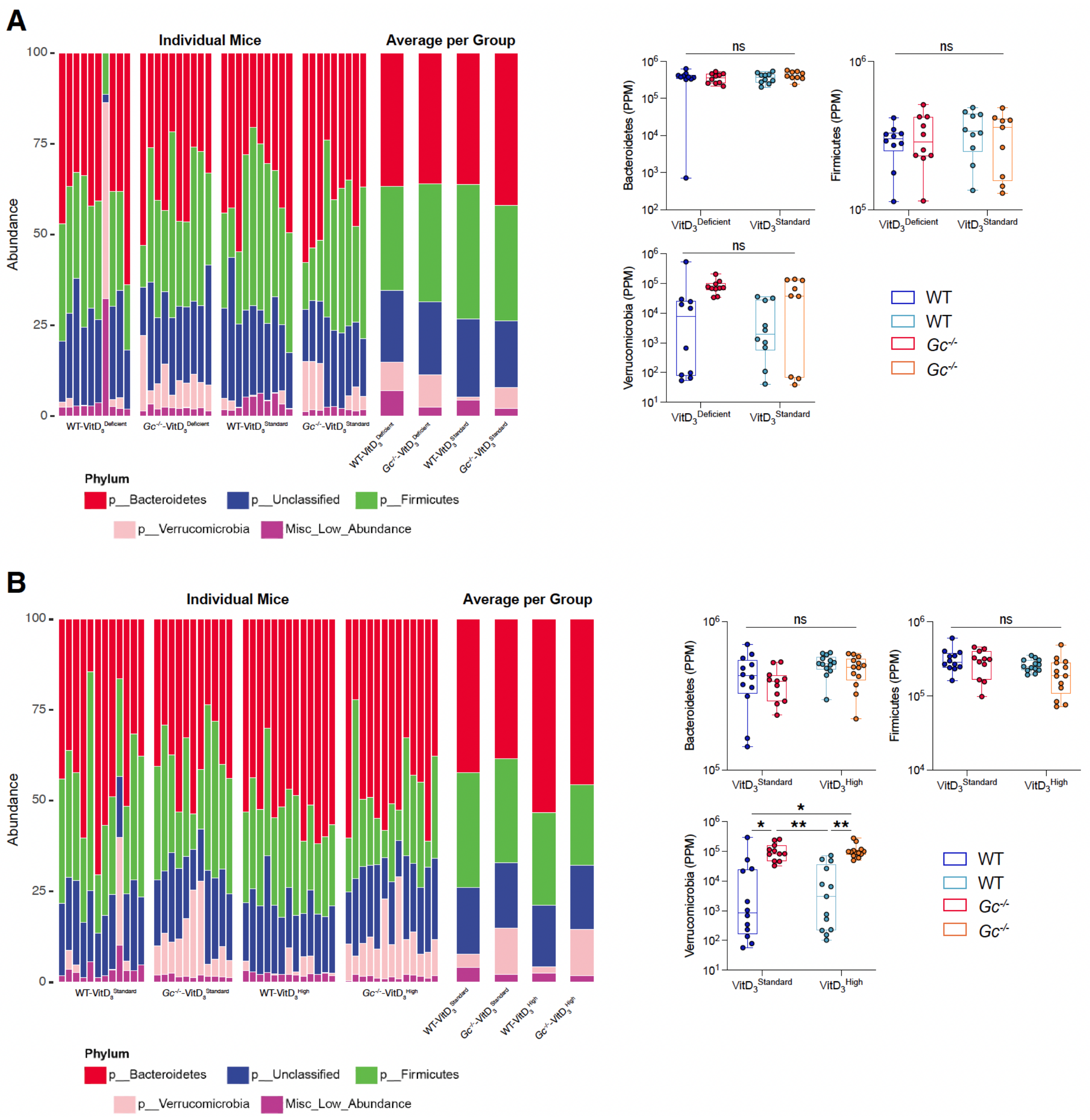

Supplementary Fig. 7. Phylum abundance in the fecal matter of mice with high or low VitD availability. (**A-B**) Relative abundance bar plots of bacterial phyla in fecal samples from separately housed groups of WT C57BL/6J or *Gc*$^{-/-}$ mice that were fed with VitD$_3$ standard (2 IU/g) or deficient (0 IU/g) **(A)** or VitD$_3$ standard (2 IU/g) or high (10 IU/g) **(B)** diet for 3.5 weeks. (A) WT + VitD$_3$^{Standard} (n=10), *Gc*$^{-/-}$ + VitD$_3$^{Standard} (n=9), WT + VitD$_3$^{Deficient} (n=10), *Gc*$^{-/-}$ + VitD$_3$^{Deficient} (n=10). (B) WT + VitD$_3$^{Standard} (n=12), *Gc*$^{-/-}$ + VitD$_3$^{Standard} (n=11), WT + VitD$_3$^{High} (n=13), *Gc*$^{-/-}$ + VitD$_3$^{High} (n=13). Data in (A, B) are shown as stacked bar plots for individual mice separated by groups (left) and averaged per group (center), and as relative abundance (PPM) box-and-whisker plots with median, 25th and 75th percentiles represented by the box and min/max by the whiskers (right).

Taxa abundances (A, B, right panel) were compared using Bonferroni-corrected two-way ANOVA. $*p<0.05$, $**p<0.01$; ns, not significant.

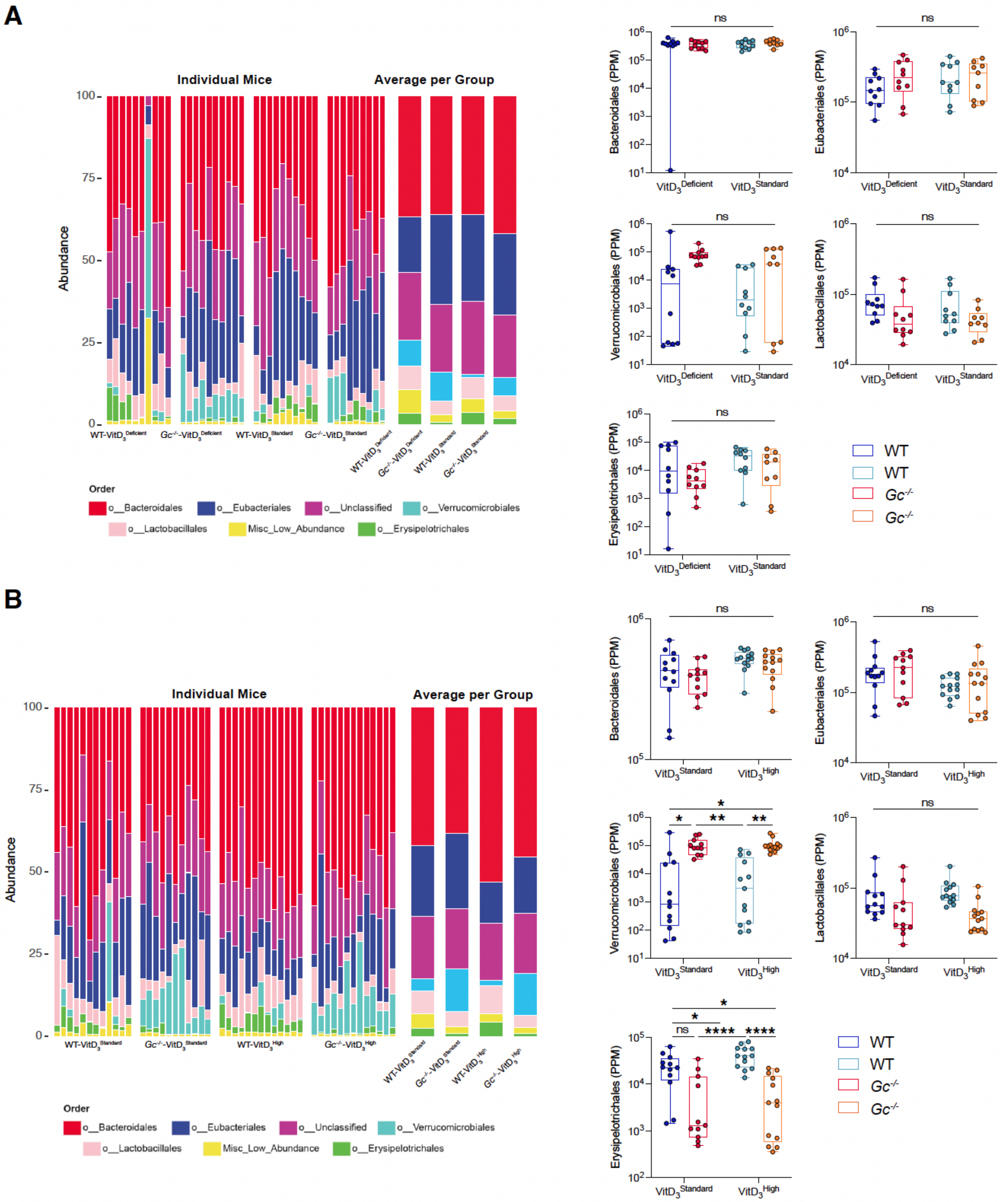

Supplementary Fig. 8. Order abundance in the fecal matter of mice with high or low VitD availability. (**A-B**) Relative abundance bar plots of bacterial orders in fecal samples from separately housed groups of WT C57BL/6J or *Gc*$^{-/-}$ mice that were fed with VitD$_3$ standard (2 IU/g) or deficient (0 IU/g) **(A)** or VitD$_3$ standard (2 IU/g) or high (10 IU/g) **(B)** diet for 3.5 weeks. (A)

WT + $VitD_3^{Standard}$ (n=10), *Gc*$^{-/-}$ + $VitD_3^{Standard}$ (n=9), WT + $VitD_3^{Deficient}$ (n=10), *Gc*$^{-/-}$ + $VitD_3^{Deficient}$ (n=10). (B) WT + $VitD_3^{Standard}$ (n=12), *Gc*$^{-/-}$ + $VitD_3^{Standard}$ (n=11), WT + $VitD_3^{High}$ (n=13), *Gc*$^{-/-}$ + $VitD_3^{High}$ (n=13). Data in (A, B) are shown as stacked bar plots for individual mice separated by groups (left) and averaged per group (center), and as relative abundance (PPM) box-and-whisker plots with median, 25th and 75th percentiles represented by the box and min/max by the whiskers (right). Taxa abundances (A, B, right panel) were compared using Bonferroni-corrected two-way ANOVA. *p<0.05, **p< 0.01, ****p< 0.0001; ns, not significant.

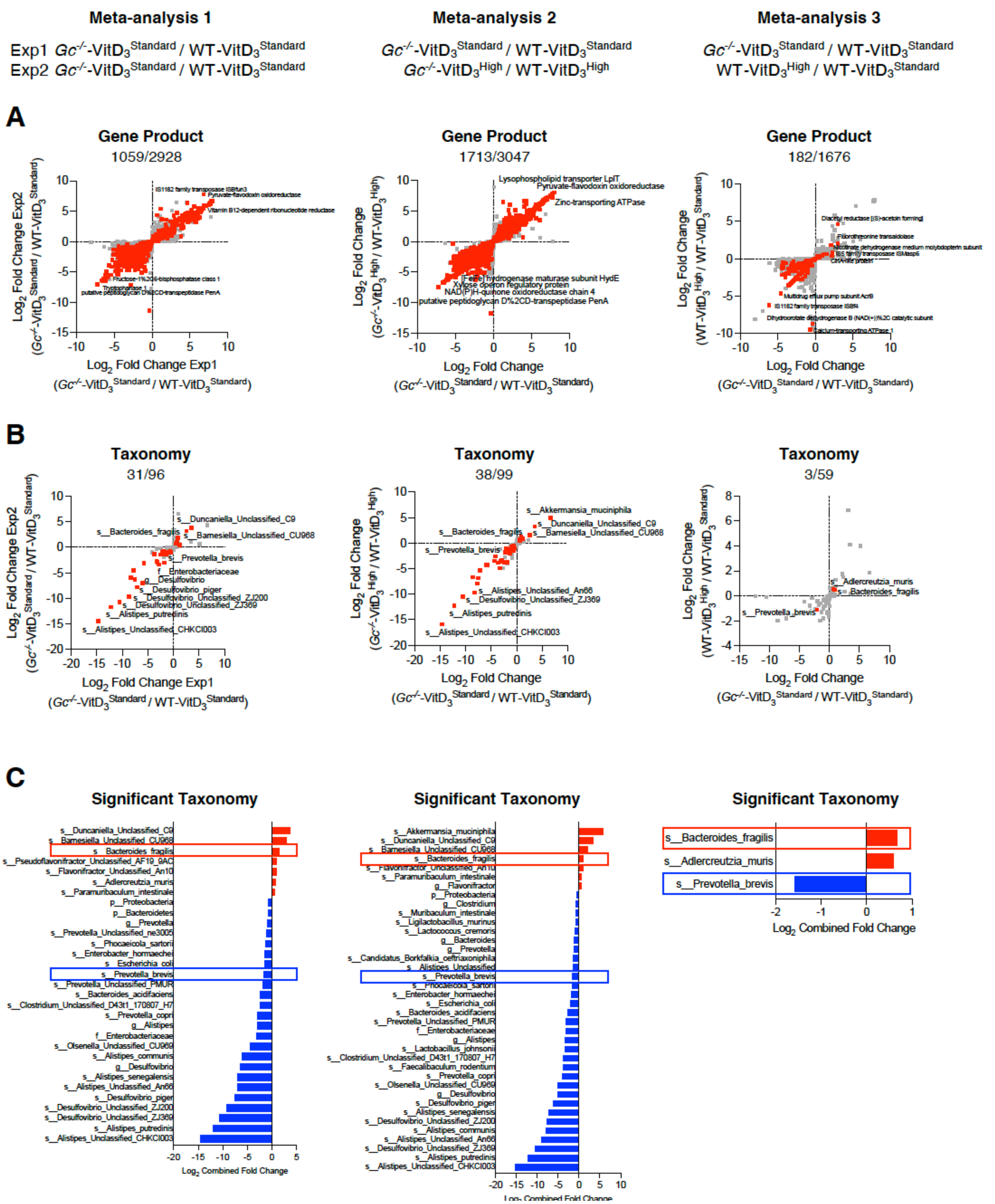

Supplementary Fig. 9. Meta analyses of taxonomic and functional bacterial features in the fecal matter of mice with high or low VitD availability. (**A-C**) Meta-analyses of indicated comparisons for (**A**) bacterial gene products and (**B, C**) taxonomy associated with differences in

VitD availability: Meta1 (left), Exp 1 WT + $VitD_3^{Standard}$ (n=10) vs. *Gc*$^{-/-}$ + $VitD_3^{Standard}$ (n=9) and Exp2 WT + $VitD_3^{Standard}$ (n=12) vs. *Gc*$^{-/-}$ + $VitD_3^{Standard}$ (n=11); Meta2 (center), WT + $VitD_3^{Standard}$ (n=12) vs. *Gc*$^{-/-}$ + $VitD_3^{Standard}$ (n=11) and WT + $VitD_3^{High}$ (n=13) vs. *Gc*$^{-/-}$ + $VitD_3^{High}$ (n=13); Meta3 (right), WT + $VitD_3^{Standard}$ (n=12) vs. *Gc*$^{-/-}$ + $VitD_3^{Standard}$ (n=11) and WT + $VitD_3^{High}$ (n=13) vs. WT + $VitD_3^{Standard}$ (n=12). Only features that showed consistent direction of fold change across both comparisons were used for the meta-analyses and plotted. Data in (A, B) are presented as $\log_2$ median fold change for each comparison and in (C) as average of $\log_2$ median fold change across both comparisons in each meta-analysis. In (A, B and C), p values were calculated using the Mann–Whitney–Wilcoxon U test on relative abundance (PPM) for that feature in samples within each group for pairwise comparisons. The combined p value (cp) for meta-analysis of within-group comparisons was calculated using Fishers P value. In (A, B) the number of significant/total features in each analysis are shown above each plot. In (C), significantly different taxonomy common across all three analyses are highlighted. For each feature type, the cut-offs for the meta-analysis were: $p < 0.2$, $cp < 0.1$, FDR<0.15 (red); not significant (grey).

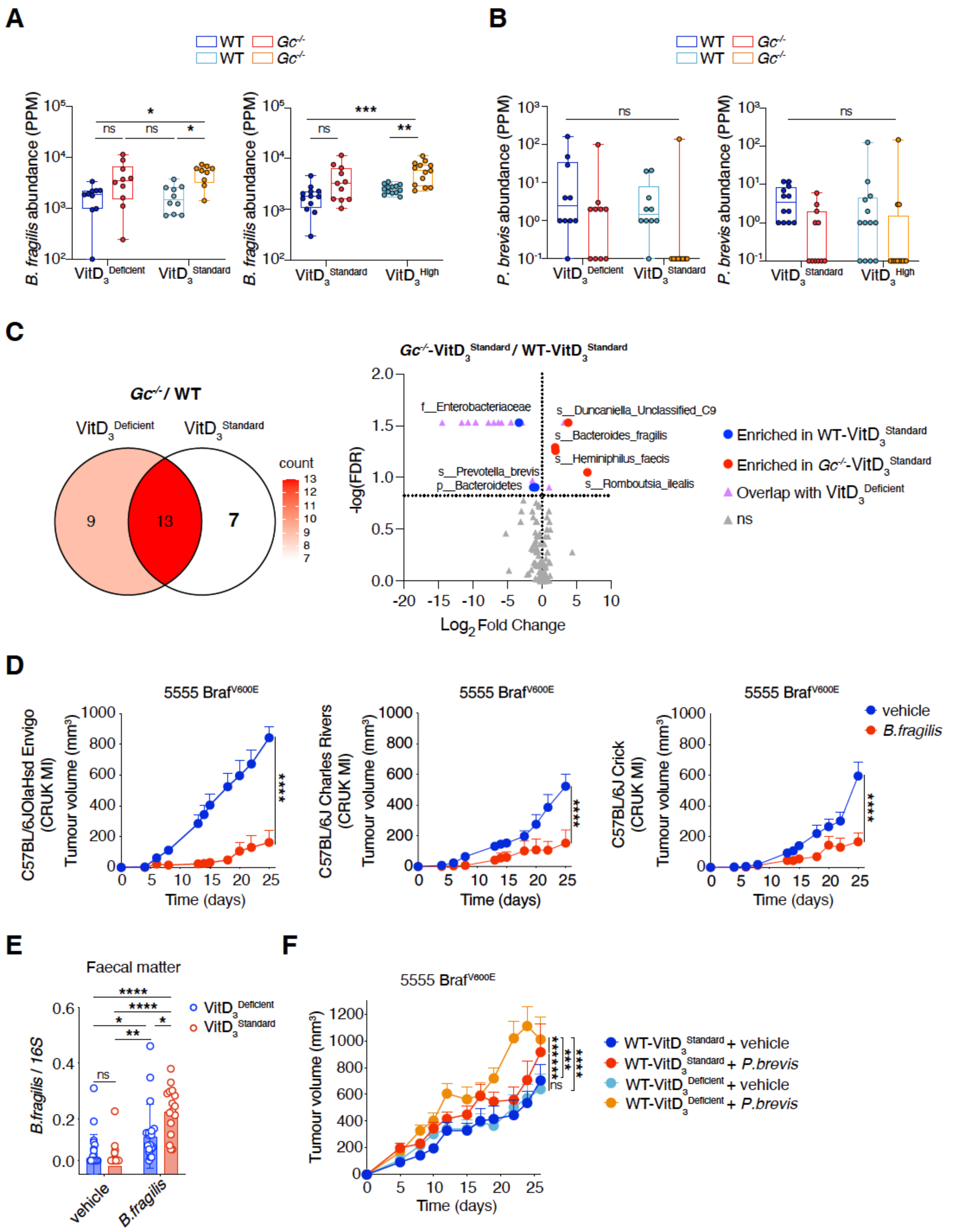

Supplementary Fig. 10. *B. fragilis* is associated with $VitD_3$ availability and enhanced tumor resistance. (**A-C**) Sequence analysis of bacterial taxa in fecal samples from separately housed

groups of WT C57BL/6J or *Gc*$^{-/-}$ mice that were fed with $VitD_3$ standard (2 IU/g) or deficient (0 IU/g) or $VitD_3$ standard (2 IU/g) or high (10 IU/g) diet for 3.5 weeks. Relative abundance of (**A**) *Bacteroides fragilis* and (**B**) *Prevotella brevis*. (**C**) Differentially abundant taxa between WT and *Gc*$^{-/-}$ present when mice are given $VitD_3^{Standard}$ but not $VitD_3^{Deficient}$; (left) Venn diagram showing number of overlapping and exclusive differentially abundant taxa across both diets and (right) volcano plot highlighting 7 taxa exclusive to $VitD_3^{Standard}$. (A, B, and C) WT + $VitD_3^{Standard}$ (n=10), *Gc*$^{-/-}$ + $VitD_3^{Standard}$ (n=9), WT + $VitD_3^{Deficient}$ (n=10), *Gc*$^{-/-}$ + $VitD_3^{Deficient}$ (n=10). (A, B) WT + $VitD_3^{Standard}$ (n=12), *Gc*$^{-/-}$ + $VitD_3^{Standard}$ (n=11), WT + $VitD_3^{High}$ (n=13), *Gc*$^{-/-}$ + $VitD_3^{High}$ (n=13). (**D**) Growth profile of 0.2 x 10^6 5555 $Braf^{V600E}$ cancer cells implanted into separately housed groups of C57BL/6JOlaHsd2 Envigo (n=10 per group), C57BL/6J Charles River (n=9 vs. n=10), C57BL/6J Crick (n=10 vs. n=9) before receiving *B. fragilis* or vehicle at CRUK Manchester Institute (CRUK MI). Mice received 10^9 *B. fragilis* by oral gavage on days -14, -12 and -10 prior to tumor inoculation. (**E**) Q-PCR quantification of 16S rRNA gene of *B. fragilis* in fecal matter of mice that were fed $VitD_3$ standard or deficient diet and received *B.fragilis* or vehicle, relative to abundance of total 16S rRNA gene. WT-$VitD_3^{Standard}$ + vehicle (n=17), WT-$VitD_3^{Deficient}$+ vehicle (n=18), WT-$VitD_3^{Standard}$ + *B.fragilis* (n=16), WT-$VitD_3^{Deficient}$ + *B.fragilis* (n=19). (**F**) Growth profile of 0.2 x 10^6 5555 $Braf^{V600E}$ cancer cells implanted into separately housed groups of WT C57BL/6J mice (n=10 per group) fed with $VitD_3$ standard or $VitD_3$ deficient diet, starting 3.5 weeks before receiving *P. brevis* or vehicle. Mice received 10^9 *P. brevis* by oral gavage on days -14, -12 and -10 prior to tumor inoculation. Data in (A, B) are presented as relative abundance (PPM) box-and-whisker plots with median, 25th and 75th percentiles represented by the box and min/max by the whiskers and are from one experiment. Data in (C) is presented as log_2 median fold change vs. -log(FDR). Data in (D) are presented as mean ± SD of two independent experiments. Data in (D and F) are presented as tumor volume (mm^3) + SEM of one experiment (D) or are representative of two independent experiments (F). Data in (C) were compared using the Mann–Whitney–Wilcoxon U test on PPM in samples within each diet for pairwise comparisons with significance indicated by $p < 0.05$, FDR < 0.15 within each comparison. Data in (A, B, D, E, and F) were compared using Bonferroni-corrected two-way ANOVA. *p<0.05, **p<0.01, ***p<0.001, ****p< 0.0001; ns, not significant.

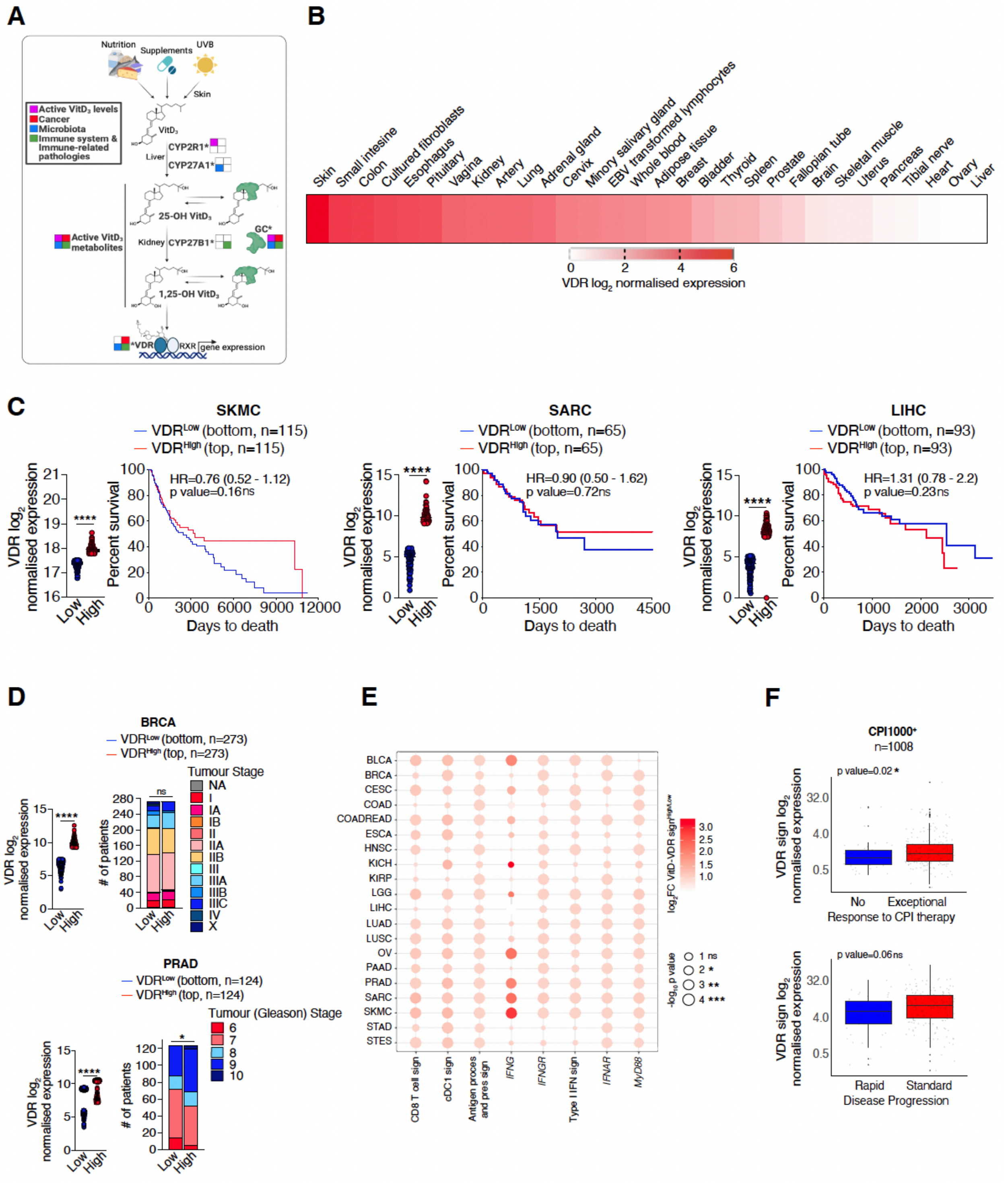

Supplementary Fig. 11. Genome-wide association studies and association of patient survival, tumor stage and response to checkpoint inhibitors with gene expression in human tumors. (**A**) Schematic representation of the correlation of VitD$_3$ metabolites and proteins involved in VitD$_3$ biosynthesis, availability or biological function with cancer risk (red square),

microbiota alterations (purple square) or immune parameters in health and disease (green square). The asterisk indicates polymorphisms in the indicated genes that are identified in genome-wide association studies correlated with at least one of the four parameters listed (coloured squares). (**B**) Human tissue expression of *VDR* from the Genotype-Tissue Expression database. (**C**) Prognostic value of *VDR* expression for overall survival and hazard ratio comparing samples with the lowest (*VDR*^{Low}) versus highest (*VDR*^{High}) expression in the indicated TCGA datasets. Skin cutaneous melanoma (SKMC, n=460), sarcoma (SARC, n=259), liver hepatocellular carcinoma (LIHC, n=370) bottom and top 25% of patient cohort. (**D**) Prognostic value of *VDR* expression for tumor stage comparing samples with the lowest (*VDR*^{Low}) versus highest (*VDR*^{High}) expression in the indicated TCGA datasets. Breast cancer (BRCA, n=1092), prostate adenocarcinoma (PRAD, n=497) bottom and top 25% of patient cohort. (**E**) Expression of the indicated immune related gene signatures and genes comparing samples with the (VitD-VDR gene sign^{High}) versus the lowest (VitD-VDR gene sign^{Low}) expression in the indicated TCGA datasets. (**F**) *VDR* expression in samples with no versus exceptional response (top) and rapid versus standard disease progression (bottom) of patients (n=1008) treated with checkpoint inhibitors (CPI1000^{+} cohort). In (C, D) data are presented as mean of log2 normalized expression ± SEM. In (E) data are presented as log2 fold change (FC) of gene or gene signature expression comparing VitD-VDR gene sign High/Low groups (colour intensity represent the magnitude of FC and the size of the circle depicts the magnitude of the probability value). In (F) data are presented as log2 normalized expression box-and-whisker plot with median, 25th and 75th percentiles represented by the box and min/max by the whiskers. Survival (Kaplan-Meier) curves in (C) were compared using Log-rank (Mantel-Cox) test. In (C) hazard ratios (HR) with 95% confidence interval showed. In (C, D and E) expression of genes and gene signatures between groups were compared using two-tailed unpaired t test with Welch's correction. In (D) frequency of tumour stage was compared between groups using Chi-squared test. In (F) gene expression between the groups was compared using Wilcoxon signed-rank test. *p<0.05, **p<0.01, ***p< 0.001; ns, not significant.

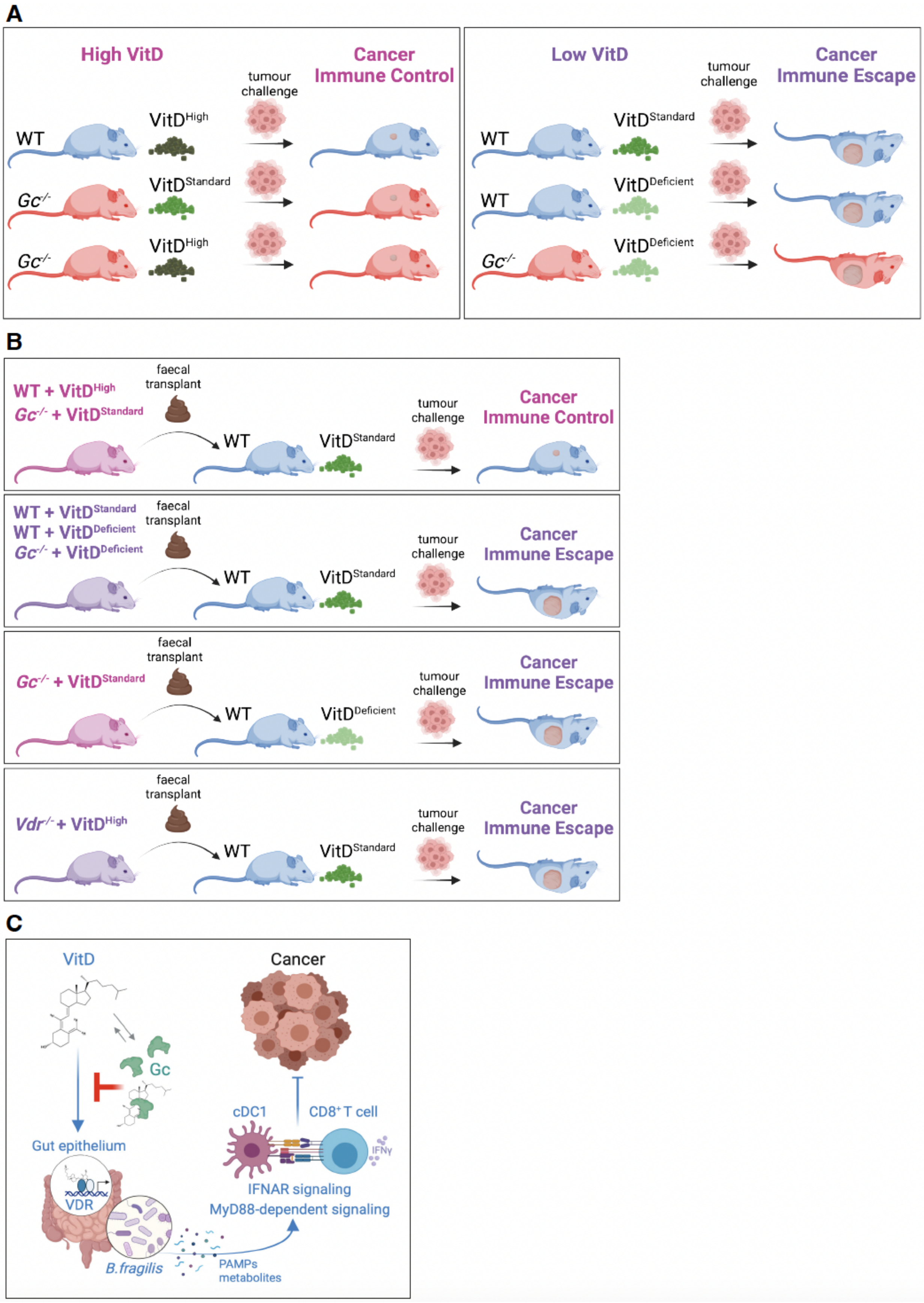
A
High VitD
Cancer Immune Control
tumour challenge
WT
VitDHigh
Gc-/-
VitDStandard
Gc-/-
VitDHigh
Low VitD
Cancer Immune Escape
tumour challenge
WT
VitDStandard
WT
VitDDeficient
Gc-/-
VitDDeficient
B
WT + VitDHigh
Gc-/- + VitDStandard
faecal transplant
WT
VitDStandard
tumour challenge
Cancer Immune Control
WT + VitDStandard
WT + VitDDeficient
Gc-/- + VitDDeficient
faecal transplant
WT
VitDStandard
tumour challenge
Cancer Immune Escape
Gc-/- + VitDStandard
faecal transplant
WT
VitDDeficient
tumour challenge
Cancer Immune Escape
Vdr-/- + VitDHigh
faecal transplant
WT
VitDStandard
tumour challenge
Cancer Immune Escape
C
VitD
Cancer
Gc
cDC1
CD8+ T cell
IFNγ
Gut epithelium
VDR
IFNAR signaling
MyD88-dependent signaling
B.fragilis
PAMPs
metabolites

Supplementary Fig. 12. Schematic summary of the findings. (**A-B**) Mice with increased VitD activity display (**A**) endogenous and (**B**) transmissible tumor resistance. (**C**) VitD acts on intestinal epithelial cells, which modulate the gut microbiome increasing a niche for *B. fragilis*, which boosts T cell responses to cancer. Limited VitD activity due to dietary restriction of VitD and sequestration by plasma Gc globulin dampens microbiome-dependent anti-cancer immunity.

Supplemental Table 1. Polymorphisms in genes regulating VitD availability correlate with cancer risk, alterations in microbiota and/or changes in immune parameters.

Supplemental Table 2. VitD-VDR gene signature in TCGA and CP1000^{+} cohorts.

Supplemental Table 3. Elution time of isotope-labelled VitD$_3$ internal standards in SFC-MS analysis.

References and Notes

1. E. A. Yamamoto, T. N. Jørgensen, Relationships Between Vitamin D, Gut Microbiome, and Systemic Autoimmunity. *Front. Immunol.* **10**, 3141 (2020). doi:10.3389/fimmu.2019.03141 Medline

2. M. Medrano, E. Carrillo-Cruz, I. Montero, J. A. Perez-Simon, Vitamin D: Effect on Haematopoiesis and Immune System and Clinical Applications. *Int. J. Mol. Sci.* **19**, 2663 (2018). doi:10.3390/ijms19092663 Medline

3. R. M. Lucas, S. Gorman, S. Geldenhuys, P. H. Hart, Vitamin D and immunity. *F1000Prime Rep.* **6**, 118 (2014). doi:10.12703/P6-118 Medline

4. A. Clark, N. Mach, Role of Vitamin D in the Hygiene Hypothesis: The Interplay between Vitamin D, Vitamin D Receptors, Gut Microbiota, and Immune Response. *Front. Immunol.* **7**, 627 (2016). doi:10.3389/fimmu.2016.00627 Medline

5. M. Waterhouse, B. Hope, L. Krause, M. Morrison, M. M. Protani, M. Zakrzewski, R. E. Neale, Vitamin D and the gut microbiome: A systematic review of in vivo studies. *Eur. J. Nutr.* **58**, 2895–2910 (2019). doi:10.1007/s00394-018-1842-7 Medline

6. J. Wang, L. B. Thingholm, J. Skiecevičienė, P. Rausch, M. Kummen, J. R. Hov, F. Degenhardt, F.-A. Heinsen, M. C. Rühlemann, S. Szymczak, K. Holm, T. Esko, J. Sun, M. Pricop-Jeckstadt, S. Al-Dury, P. Bohov, J. Bethune, F. Sommer, D. Ellinghaus, R. K. Berge, M. Hübenthal, M. Koch, K. Schwarz, G. Rimbach, P. Hübbe, W.-H. Pan, R. Sheibani-Tezerji, R. Häsler, P. Rosenstiel, M. D'Amato, K. Cloppenborg-Schmidt, S. Künzel, M. Laudes, H.-U. Marschall, W. Lieb, U. Nöthlings, T. H. Karlsen, J. F. Baines, A. Franke, Genome-wide association analysis identifies variation in vitamin D receptor and other host factors influencing the gut microbiota. *Nat. Genet.* **48**, 1396–1406 (2016). doi:10.1038/ng.3695 Medline

7. K. K. Deeb, D. L. Trump, C. S. Johnson, Vitamin D signalling pathways in cancer: Potential for anticancer therapeutics. *Nat. Rev. Cancer* **7**, 684–700 (2007). doi:10.1038/nrc2196 Medline

8. S.-M. Jeon, E.-A. Shin, Exploring vitamin D metabolism and function in cancer. *Exp. Mol. Med.* **50**, 1–14 (2018). doi:10.1038/s12276-018-0038-9 Medline

9. C. Carlberg, A. Muñoz, An update on vitamin D signaling and cancer. *Semin. Cancer Biol.* **79**, 217–230 (2022). doi:10.1016/j.semcancer.2020.05.018 Medline

10. M. H. Sherman, R. T. Yu, D. D. Engle, N. Ding, A. R. Atkins, H. Tiriac, E. A. Collisson, F. Connor, T. Van Dyke, S. Kozlov, P. Martin, T. W. Tseng, D. W. Dawson, T. R. Donahue, A. Masamune, T. Shimosegawa, M. V. Apte, J. S. Wilson, B. Ng, S. L. Lau, J. E. Gunton, G. M. Wahl, T. Hunter, J. A. Drebin, P. J. O'Dwyer, C. Liddle, D. A. Tuveson, M. Downes, R. M. Evans, Vitamin D receptor-mediated stromal reprogramming suppresses pancreatitis and enhances pancreatic cancer therapy. *Cell* **159**, 80–93 (2014). doi:10.1016/j.cell.2014.08.007 Medline

11. G. Ferrer-Mayorga, G. Gómez-López, A. Barbáchano, A. Fernández-Barral, C. Peña, D. G. Pisano, R. Cantero, F. Rojo, A. Muñoz, M. J. Larriba, Vitamin D receptor expression and

associated gene signature in tumour stromal fibroblasts predict clinical outcome in colorectal cancer. *Gut* **66**, 1449–1462 (2017). doi:10.1136/gutjnl-2015-310977 Medline

12. J. Wactawski-Wende, J. M. Kotchen, G. L. Anderson, A. R. Assaf, R. L. Brunner, M. J. O'Sullivan, K. L. Margolis, J. K. Ockene, L. Phillips, L. Pottern, R. L. Prentice, J. Robbins, T. E. Rohan, G. E. Sarto, S. Sharma, M. L. Stefanick, L. Van Horn, R. B. Wallace, E. Whitlock, T. Bassford, S. A. A. Beresford, H. R. Black, D. E. Bonds, R. G. Brzyski, B. Caan, R. T. Chlebowski, B. Cochrane, C. Garland, M. Gass, J. Hays, G. Heiss, S. L. Hendrix, B. V. Howard, J. Hsia, F. A. Hubbell, R. D. Jackson, K. C. Johnson, H. Judd, C. L. Kooperberg, L. H. Kuller, A. Z. LaCroix, D. S. Lane, R. D. Langer, N. L. Lasser, C. E. Lewis, M. C. Limacher, J. E. Manson, Women's Health Initiative Investigators, Calcium plus vitamin D supplementation and the risk of colorectal cancer. *N. Engl. J. Med.* **354**, 684–696 (2006). doi:10.1056/NEJMoa055222 Medline

13. M. Jenab, H. B. Bueno-de-Mesquita, P. Ferrari, F. J. B. van Duijnhoven, T. Norat, T. Pischon, E. H. J. M. Jansen, N. Slimani, G. Byrnes, S. Rinaldi, A. Tjønneland, A. Olsen, K. Overvad, M.-C. Boutron-Ruault, F. Clavel-Chapelon, S. Morois, R. Kaaks, J. Linseisen, H. Boeing, M. M. Bergmann, A. Trichopoulou, G. Misirli, D. Trichopoulos, F. Berrino, P. Vineis, S. Panico, D. Palli, R. Tumino, M. M. Ros, C. H. van Gils, P. H. Peeters, M. Brustad, E. Lund, M.-J. Tormo, E. Ardanaz, L. Rodríguez, M.-J. Sánchez, M. Dorronsoro, C. A. Gonzalez, G. Hallmans, R. Palmqvist, A. Roddam, T. J. Key, K.-T. Khaw, P. Autier, P. Hainaut, E. Riboli, Association between pre-diagnostic circulating vitamin D concentration and risk of colorectal cancer in European populations:a nested case-control study. *BMJ* **340**, b5500 (2010). doi:10.1136/bmj.b5500 Medline

14. C. G. Woolcott, L. R. Wilkens, A. M. Y. Nomura, R. L. Horst, M. T. Goodman, S. P. Murphy, B. E. Henderson, L. N. Kolonel, L. Le Marchand, Plasma 25-hydroxyvitamin D levels and the risk of colorectal cancer: The multiethnic cohort study. *Cancer Epidemiol. Biomarkers Prev.* **19**, 130–134 (2010). doi:10.1158/1055-9965.EPI-09-0475 Medline

15. S. Gandini, M. Boniol, J. Haukka, G. Byrnes, B. Cox, M. J. Sneyd, P. Mullie, P. Autier, Meta-analysis of observational studies of serum 25-hydroxyvitamin D levels and colorectal, breast and prostate cancer and colorectal adenoma. *Int. J. Cancer* **128**, 1414–1424 (2011). doi:10.1002/ijc.25439 Medline

16. Y. Ma, P. Zhang, F. Wang, J. Yang, Z. Liu, H. Qin, Association between vitamin D and risk of colorectal cancer: A systematic review of prospective studies. *J. Clin. Oncol.* **29**, 3775–3782 (2011). doi:10.1200/JCO.2011.35.7566 Medline

17. J. De Smedt, S. Van Kelst, V. Boecxstaens, M. Stas, K. Bogaerts, D. Vanderschueren, C. Aura, K. Vandenberghe, D. Lambrechts, P. Wolter, O. Bechter, A. Nikkels, T. Strobbe, G. Emri, V. Marasigan, M. Garmyn, Vitamin D supplementation in cutaneous malignant melanoma outcome (ViDMe): A randomized controlled trial. *BMC Cancer* **17**, 562 (2017). doi:10.1186/s12885-017-3538-4 Medline

18. L. Yang, H. Chen, M. Zhao, P. Peng, Prognostic value of circulating vitamin D binding protein, total, free and bioavailable 25-hydroxy vitamin D in patients with colorectal cancer. *Oncotarget* **8**, 40214–40221 (2017). doi:10.18632/oncotarget.16597 Medline

19. J. E. Manson, N. R. Cook, I.-M. Lee, W. Christen, S. S. Bassuk, S. Mora, H. Gibson, D. Gordon, T. Copeland, D. D'Agostino, G. Friedenberg, C. Ridge, V. Bubes, E. L.

Giovannucci, W. C. Willett, J. E. Buring, VITAL Research Group, Vitamin D Supplements and Prevention of Cancer and Cardiovascular Disease. *N. Engl. J. Med.* **380**, 33–44 (2019). doi:10.1056/NEJMoa1809944 Medline

20. K. Ng, H. S. Nimeiri, N. J. McCleary, T. A. Abrams, M. B. Yurgelun, J. M. Cleary, D. A. Rubinson, D. Schrag, R. Miksad, A. J. Bullock, J. Allen, D. Zuckerman, E. Chan, J. A. Chan, B. M. Wolpin, M. Constantine, D. J. Weckstein, M. A. Faggen, C. A. Thomas, C. Kournioti, C. Yuan, C. Ganser, B. Wilkinson, C. Mackintosh, H. Zheng, B. W. Hollis, J. A. Meyerhardt, C. S. Fuchs, Effect of High-Dose vs Standard-Dose Vitamin D_3 Supplementation on Progression-Free Survival Among Patients With Advanced or Metastatic Colorectal Cancer: The SUNSHINE Randomized Clinical Trial. *JAMA* **321**, 1370–1379 (2019). doi:10.1001/jama.2019.2402 Medline

21. M. Urashima, H. Ohdaira, T. Akutsu, S. Okada, M. Yoshida, M. Kitajima, Y. Suzuki, Effect of Vitamin D Supplementation on Relapse-Free Survival Among Patients With Digestive Tract Cancers: The AMATERASU Randomized Clinical Trial. *JAMA* **321**, 1361–1369 (2019). doi:10.1001/jama.2019.2210 Medline

22. D. D. Bikle, Vitamin D metabolism, mechanism of action, and clinical applications. *Chem. Biol.* **21**, 319–329 (2014). doi:10.1016/j.chembiol.2013.12.016 Medline

23. J. G. Haddad, Y. Z. Hu, M. A. Kowalski, C. Laramore, K. Ray, P. Robzyk, N. E. Cooke, Identification of the sterol- and actin-binding domains of plasma vitamin D binding protein (Gc-globulin). *Biochemistry* **31**, 7174–7181 (1992). doi:10.1021/bi00146a021 Medline

24. F. F. Safadi, P. Thornton, H. Magiera, B. W. Hollis, M. Gentile, J. G. Haddad, S. A. Liebhaber, N. E. Cooke, Osteopathy and resistance to vitamin D toxicity in mice null for vitamin D binding protein. *J. Clin. Invest.* **103**, 239–251 (1999). doi:10.1172/JCI5244 Medline

25. E. G. Duchow, N. E. Cooke, J. Seeman, L. A. Plum, H. F. DeLuca, Vitamin D binding protein is required to utilize skin-generated vitamin D. *Proc. Natl. Acad. Sci. U.S.A.* **116**, 24527–24532 (2019). doi:10.1073/pnas.1915442116 Medline

26. C. M. Henderson, S. L. Fink, H. Bassyouni, B. Argiropoulos, L. Brown, T. J. Laha, K. J. Jackson, R. Lewkonia, P. Ferreira, A. N. Hoofnagle, J. L. Marcadier, Vitamin D–Binding Protein Deficiency and Homozygous Deletion of the *GC* Gene. *N. Engl. J. Med.* **380**, 1150–1157 (2019). doi:10.1056/NEJMoa1807841 Medline

27. J. P. Böttcher, C. Reis e Sousa, The Role of Type 1 Conventional Dendritic Cells in Cancer Immunity. *Trends Cancer* **4**, 784–792 (2018). doi:10.1016/j.trecan.2018.09.001 Medline

28. S. K. Wculek, F. J. Cueto, A. M. Mujal, I. Melero, M. F. Krummel, D. Sancho, Dendritic cells in cancer immunology and immunotherapy. *Nat. Rev. Immunol.* **20**, 7–24 (2020). doi:10.1038/s41577-019-0210-z Medline

29. J. Canton, H. Blees, C. M. Henry, M. D. Buck, O. Schulz, N. C. Rogers, E. Childs, S. Zelenay, H. Rhys, M.-C. Domart, L. Collinson, A. Alloatti, C. J. Ellison, S. Amigorena, V. Papayannopoulos, D. C. Thomas, F. Randow, C. Reis e Sousa, The receptor DNGR-1 signals for phagosomal rupture to promote cross-presentation of dead-cell-associated antigens. *Nat. Immunol.* **22**, 140–153 (2021). doi:10.1038/s41590-020-00824-x Medline

30. C. M. Henry, C. A. Castellanos, C. Reis e Sousa, DNGR-1-mediated cross-presentation of dead cell-associated antigens. *Semin. Immunol.* **66**, 101726 (2023). doi:10.1016/j.smim.2023.101726 Medline

31. E. Giampazolias, O. Schulz, K. H. J. Lim, N. C. Rogers, P. Chakravarty, N. Srinivasan, O. Gordon, A. Cardoso, M. D. Buck, E. Z. Poirier, J. Canton, S. Zelenay, S. Sammicheli, N. Moncaut, S. Varsani-Brown, I. Rosewell, C. Reis e Sousa, Secreted gelsolin inhibits DNGR-1-dependent cross-presentation and cancer immunity. *Cell* **184**, 4016–4031.e22 (2021). doi:10.1016/j.cell.2021.05.021 Medline

32. K. H. J. Lim, E. Giampazolias, O. Schulz, N. C. Rogers, A. Wilkins, E. Sahai, J. Strid, C. Reis e Sousa, Loss of secreted gelsolin enhances response to anticancer therapies. *J. Immunother. Cancer* **10**, e005245 (2022). doi:10.1136/jitc-2022-005245 Medline

33. W. M. Lee, R. M. Galbraith, The extracellular actin-scavenger system and actin toxicity. *N. Engl. J. Med.* **326**, 1335–1341 (1992). doi:10.1056/NEJM199205143262006 Medline

34. O. Koivisto, A. Hanel, C. Carlberg, Key Vitamin D Target Genes with Functions in the Immune System. *Nutrients* **12**, 1140 (2020). doi:10.3390/nu12041140 Medline

35. M. Amling, M. Priemel, T. Holzmann, K. Chapin, J. M. Rueger, R. Baron, M. B. Demay, Rescue of the skeletal phenotype of vitamin D receptor-ablated mice in the setting of normal mineral ion homeostasis: Formal histomorphometric and biomechanical analyses. *Endocrinology* **140**, 4982–4987 (1999). doi:10.1210/endo.140.11.7110 Medline

36. A. M. Mondul, I. M. Shui, K. Yu, R. C. Travis, V. L. Stevens, D. Campa, F. R. Schumacher, R. G. Ziegler, H. B. Bueno-de-Mesquita, S. Berndt, E. D. Crawford, S. M. Gapstur, J. M. Gaziano, E. Giovannucci, C. A. Haiman, B. E. Henderson, D. J. Hunter, M. Johansson, T. J. Key, L. Le Marchand, S. Lindström, M. L. McCullough, C. Navarro, K. Overvad, D. Palli, M. Purdue, M. J. Stampfer, S. J. Weinstein, W. C. Willett, M. Yeager, S. J. Chanock, D. Trichopoulos, L. N. Kolonel, P. Kraft, D. Albanes, Genetic variation in the vitamin D pathway in relation to risk of prostate cancer—Results from the breast and prostate cancer cohort consortium. *Cancer Epidemiol. Biomarkers Prev.* **22**, 688–696 (2013). doi:10.1158/1055-9965.EPI-13-0007-T Medline

37. L. Zhou, X. Zhang, X. Chen, L. Liu, C. Lu, X. Tang, J. Shi, M. Li, M. Zhou, Z. Zhang, L. Xiao, M. Yang, GC Glu416Asp and Thr420Lys polymorphisms contribute to gastrointestinal cancer susceptibility in a Chinese population. *Int. J. Clin. Exp. Med.* **5**, 72–79 (2012). Medline

38. S. Karami, G. Andreotti, S. Koutros, K. H. Barry, L. E. Moore, S. Han, J. A. Hoppin, D. P. Sandler, J. H. Lubin, L. A. Burdette, J. Yuenger, M. Yeager, L. E. B. Freeman, A. Blair, M. C. R. Alavanja, Pesticide exposure and inherited variants in vitamin D pathway genes in relation to prostate cancer. *Cancer Epidemiol. Biomarkers Prev.* **22**, 1557–1566 (2013). doi:10.1158/1055-9965.EPI-12-1454 Medline

39. M. Peña-Chilet, M. Ibarrola-Villava, M. Martin-González, M. Feito, C. Gomez-Fernandez, D. Planelles, G. Carretero, A. Lluch, E. Nagore, G. Ribas, rs12512631 on the group specific complement (vitamin D-binding protein *GC*) implicated in melanoma susceptibility. *PLOS ONE* **8**, e59607 (2013). doi:10.1371/journal.pone.0059607 Medline

40. L. N. Anderson, M. Cotterchio, D. E. C. Cole, J. A. Knight, Vitamin D-related genetic variants, interactions with vitamin D exposure, and breast cancer risk among Caucasian women in Ontario. *Cancer Epidemiol. Biomarkers Prev.* **20**, 1708–1717 (2011). doi:10.1158/1055-9965.EPI-11-0300 Medline

41. R. F. Chun, New perspectives on the vitamin D binding protein. *Cell Biochem. Funct.* **30**, 445–456 (2012). doi:10.1002/cbf.2835 Medline

42. S. V. Ramagopalan, A. Heger, A. J. Berlanga, N. J. Maugeri, M. R. Lincoln, A. Burrell, L. Handunnetthi, A. E. Handel, G. Disanto, S. M. Orton, C. T. Watson, J. M. Morahan, G. Giovannoni, C. P. Ponting, G. C. Ebers, J. C. Knight, A ChIP-seq defined genome-wide map of vitamin D receptor binding: Associations with disease and evolution. *Genome Res.* **20**, 1352–1360 (2010). doi:10.1101/gr.107920.110 Medline

43. N. Ding, R. T. Yu, N. Subramaniam, M. H. Sherman, C. Wilson, R. Rao, M. Leblanc, S. Coulter, M. He, C. Scott, S. L. Lau, A. R. Atkins, G. D. Barish, J. E. Gunton, C. Liddle, M. Downes, R. M. Evans, A vitamin D receptor/SMAD genomic circuit gates hepatic fibrotic response. *Cell* **153**, 601–613 (2013). doi:10.1016/j.cell.2013.03.028 Medline

44. V. Nurminen, S. Seuter, C. Carlberg, Primary Vitamin D Target Genes of Human Monocytes. *Front. Physiol.* **10**, 194 (2019). doi:10.3389/fphys.2019.00194 Medline

45. M. Kawai, S. Kinoshita, M. Yamazaki, K. Yamamoto, C. J. Rosen, S. Shimba, K. Ozono, T. Michigami, Intestinal clock system regulates skeletal homeostasis. *JCI Insight* **4**, e121798 (2019). doi:10.1172/jci.insight.121798 Medline

46. A. Hanel, A. Neme, M. Malinen, E. Hämäläinen, H.-R. Malmberg, S. Etheve, T.-P. Tuomainen, J. K. Virtanen, I. Bendik, C. Carlberg, Common and personal target genes of the micronutrient vitamin D in primary immune cells from human peripheral blood. *Sci. Rep.* **10**, 21051 (2020). doi:10.1038/s41598-020-78288-0 Medline

47. K. Litchfield, J. L. Reading, C. Puttick, K. Thakkar, C. Abbosh, R. Bentham, T. B. K. Watkins, R. Rosenthal, D. Biswas, A. Rowan, E. Lim, M. Al Bakir, V. Turati, J. A. Guerra-Assunção, L. Conde, A. J. S. Furness, S. K. Saini, S. R. Hadrup, J. Herrero, S.-H. Lee, P. Van Loo, T. Enver, J. Larkin, M. D. Hellmann, S. Turajlic, S. A. Quezada, N. McGranahan, C. Swanton, Meta-analysis of tumor- and T cell-intrinsic mechanisms of sensitization to checkpoint inhibition. *Cell* **184**, 596–614.e14 (2021). doi:10.1016/j.cell.2021.01.002 Medline

48. M. L. Gjerstorff, The Danish Cancer Registry. *Scand. J. Public Health* **39**, 42–45 (2011). doi:10.1177/1403494810393562 Medline

49. J. F. H. Arendt, A. T. Hansen, S. A. Ladefoged, H. T. Sørensen, L. Pedersen, K. Adelborg, Existing Data Sources in Clinical Epidemiology: Laboratory Information System Databases in Denmark. *Clin. Epidemiol.* **12**, 469–475 (2020). doi:10.2147/CLEP.S245060 Medline

50. H. Quan, V. Sundararajan, P. Halfon, A. Fong, B. Burnand, J.-C. Luthi, L. D. Saunders, C. A. Beck, T. E. Feasby, W. A. Ghali, Coding algorithms for defining comorbidities in ICD-9-CM and ICD-10 administrative data. *Med. Care* **43**, 1130–1139 (2005). doi:10.1097/01.mlr.0000182534.19832.83 Medline

51. C.-B. Zhou, Y.-L. Zhou, J.-Y. Fang, Gut Microbiota in Cancer Immune Response and Immunotherapy. *Trends Cancer* **7**, 647–660 (2021). doi:10.1016/j.trecan.2021.01.010 Medline

52. C. Villemin, A. Six, B. A. Neville, T. D. Lawley, M. J. Robinson, G. Bakdash, The heightened importance of the microbiome in cancer immunotherapy. *Trends Immunol.* **44**, 44–59 (2023). doi:10.1016/j.it.2022.11.002 Medline

53. M. Alexander, P. J. Turnbaugh, Deconstructing Mechanisms of Diet-Microbiome-Immune Interactions. *Immunity* **53**, 264–276 (2020). doi:10.1016/j.immuni.2020.07.015 Medline

54. A. Sivan, L. Corrales, N. Hubert, J. B. Williams, K. Aquino-Michaels, Z. M. Earley, F. W. Benyamin, Y. Man Lei, B. Jabri, M.-L. Alegre, E. B. Chang, T. F. Gajewski, Commensal *Bifidobacterium* promotes antitumor immunity and facilitates anti-PD-L1 efficacy. *Science* **350**, 1084–1089 (2015). doi:10.1126/science.aac4255 Medline

55. M. Vétizou, J. M. Pitt, R. Daillère, P. Lepage, N. Waldschmitt, C. Flament, S. Rusakiewicz, B. Routy, M. P. Roberti, C. P. M. Duong, V. Poirier-Colame, A. Roux, S. Becharef, S. Formenti, E. Golden, S. Cording, G. Eberl, A. Schlitzer, F. Ginhoux, S. Mani, T. Yamazaki, N. Jacquelot, D. P. Enot, M. Bérard, J. Nigou, P. Opolon, A. Eggermont, P.-L. Woerther, E. Chachaty, N. Chaput, C. Robert, C. Mateus, G. Kroemer, D. Raoult, I. G. Boneca, F. Carbonnel, M. Chamaillard, L. Zitvogel, Anticancer immunotherapy by CTLA-4 blockade relies on the gut microbiota. *Science* **350**, 1079–1084 (2015). doi:10.1126/science.aad1329 Medline

56. B. Routy, E. Le Chatelier, L. Derosa, C. P. M. Duong, M. T. Alou, R. Daillère, A. Fluckiger, M. Messaoudene, C. Rauber, M. P. Roberti, M. Fidelle, C. Flament, V. Poirier-Colame, P. Opolon, C. Klein, K. Iribarren, L. Mondragón, N. Jacquelot, B. Qu, G. Ferrere, C. Clémenson, L. Mezquita, J. R. Masip, C. Naltet, S. Brosseau, C. Kaderbhai, C. Richard, H. Rizvi, F. Levenez, N. Galleron, B. Quinquis, N. Pons, B. Ryffel, V. Minard-Colin, P. Gonin, J. C. Soria, E. Deutsch, Y. Loriot, F. Ghiringhelli, G. Zalcman, F. Goldwasser, B. Escudier, M. D. Hellmann, A. Eggermont, D. Raoult, L. Albiges, G. Kroemer, L. Zitvogel, Gut microbiome influences efficacy of PD-1–based immunotherapy against epithelial tumors. *Science* **359**, 91–97 (2018). doi:10.1126/science.aan3706 Medline

57. T. Tanoue, S. Morita, D. R. Plichta, A. N. Skelly, W. Suda, Y. Sugiura, S. Narushima, H. Vlamakis, I. Motoo, K. Sugita, A. Shiota, K. Takeshita, K. Yasuma-Mitobe, D. Riethmacher, T. Kaisho, J. M. Norman, D. Mucida, M. Suematsu, T. Yaguchi, V. Bucci, T. Inoue, Y. Kawakami, B. Olle, B. Roberts, M. Hattori, R. J. Xavier, K. Atarashi, K. Honda, A defined commensal consortium elicits CD8 T cells and anti-cancer immunity. *Nature* **565**, 600–605 (2019). doi:10.1038/s41586-019-0878-z Medline

58. V. Gopalakrishnan, C. N. Spencer, L. Nezi, A. Reuben, M. C. Andrews, T. V. Karpinets, P. A. Prieto, D. Vicente, K. Hoffman, S. C. Wei, A. P. Cogdill, L. Zhao, C. W. Hudgens, D. S. Hutchinson, T. Manzo, M. Petaccia de Macedo, T. Cotechini, T. Kumar, W. S. Chen, S. M. Reddy, R. Szczepaniak Sloane, J. Galloway-Pena, H. Jiang, P. L. Chen, E. J. Shpall, K. Rezvani, A. M. Alousi, R. F. Chemaly, S. Shelburne, L. M. Vence, P. C. Okhuysen, V. B. Jensen, A. G. Swennes, F. McAllister, E. Marcelo Riquelme Sanchez, Y. Zhang, E. Le Chatelier, L. Zitvogel, N. Pons, J. L. Austin-Breneman, L. E. Haydu, E. M. Burton, J. M. Gardner, E. Sirmans, J. Hu, A. J. Lazar, T. Tsujikawa, A. Diab, H.

Tawbi, I. C. Glitza, W. J. Hwu, S. P. Patel, S. E. Woodman, R. N. Amaria, M. A. Davies, J. E. Gershenwald, P. Hwu, J. E. Lee, J. Zhang, L. M. Coussens, Z. A. Cooper, P. A. Futreal, C. R. Daniel, N. J. Ajami, J. F. Petrosino, M. T. Tetzlaff, P. Sharma, J. P. Allison, R. R. Jenq, J. A. Wargo, Gut microbiome modulates response to anti–PD-1 immunotherapy in melanoma patients. *Science* **359**, 97–103 (2018). doi:10.1126/science.aan4236 Medline

59. L. F. Mager, R. Burkhard, N. Pett, N. C. A. Cooke, K. Brown, H. Ramay, S. Paik, J. Stagg, R. A. Groves, M. Gallo, I. A. Lewis, M. B. Geuking, K. D. McCoy, Microbiome-derived inosine modulates response to checkpoint inhibitor immunotherapy. *Science* **369**, 1481–1489 (2020). doi:10.1126/science.abc3421 Medline

60. K. C. Lam, R. E. Araya, A. Huang, Q. Chen, M. Di Modica, R. R. Rodrigues, A. Lopès, S. B. Johnson, B. Schwarz, E. Bohrnsen, A. P. Cogdill, C. M. Bosio, J. A. Wargo, M. P. Lee, R. S. Goldszmid, Microbiota triggers STING-type I IFN-dependent monocyte reprogramming of the tumor microenvironment. *Cell* **184**, 5338–5356.e21 (2021). doi:10.1016/j.cell.2021.09.019 Medline

61. C.-H. Lo, D.-C. Wu, S.-W. Jao, C.-C. Wu, C.-Y. Lin, C.-H. Chuang, Y.-B. Lin, C.-H. Chen, Y.-T. Chen, J.-H. Chen, K.-H. Hsiao, Y.-J. Chen, Y.-T. Chen, J.-Y. Wang, L.-H. Li, Enrichment of *Prevotella intermedia* in human colorectal cancer and its additive effects with *Fusobacterium nucleatum* on the malignant transformation of colorectal adenomas. *J. Biomed. Sci.* **29**, 88 (2022). doi:10.1186/s12929-022-00869-0 Medline

62. N. Charoenngam, A. Shirvani, T. A. Kalajian, A. Song, M. F. Holick, The Effect of Various Doses of Oral Vitamin D_3 Supplementation on Gut Microbiota in Healthy Adults: A Randomized, Double-blinded, Dose-response Study. *Anticancer Res.* **40**, 551–556 (2020). doi:10.21873/anticanres.13984 Medline

63. P. Singh, A. Rawat, M. Alwakeel, E. Sharif, S. Al Khodor, The potential role of vitamin D supplementation as a gut microbiota modifier in healthy individuals. *Sci. Rep.* **10**, 21641 (2020). doi:10.1038/s41598-020-77806-4 Medline

64. C. E. Talsness, J. Penders, E. H. J. M. Jansen, J. Damoiseaux, C. Thijs, M. Mommers, Influence of vitamin D on key bacterial taxa in infant microbiota in the KOALA Birth Cohort Study. *PLOS ONE* **12**, e0188011 (2017). doi:10.1371/journal.pone.0188011 Medline

65. S. Patrick, A tale of two habitats: *Bacteroides fragilis*, a lethal pathogen and resident in the human gastrointestinal microbiome. *Microbiology* **168**, 001156 (2022). doi:10.1099/mic.0.001156 Medline

66. M. H. Sofi, Y. Wu, T. Ticer, S. Schutt, D. Bastian, H.-J. Choi, L. Tian, C. Mealer, C. Liu, C. Westwater, K. E. Armeson, A. V. Alekseyenko, X.-Z. Yu, A single strain of Bacteroides fragilis protects gut integrity and reduces GVHD. *JCI Insight* **6**, e136841 (2021). doi:10.1172/jci.insight.136841 Medline

67. Y. K. Lee, P. Mehrabian, S. Boyajian, W.-L. Wu, J. Selicha, S. Vonderfecht, S. K. Mazmanian, The Protective Role of *Bacteroides fragilis* in a Murine Model of Colitis-Associated Colorectal Cancer. *MSphere* **3**, e00587-18 (2018). doi:10.1128/mSphere.00587-18 Medline

68. J. A. Newton-Bishop, S. Beswick, J. Randerson-Moor, Y.-M. Chang, P. Affleck, F. Elliott, M. Chan, S. Leake, B. Karpavicius, S. Haynes, K. Kukalizch, L. Whitaker, S. Jackson, E. Gerry, C. Nolan, C. Bertram, J. Marsden, D. E. Elder, J. H. Barrett, D. T. Bishop, Serum 25-hydroxyvitamin D_3 levels are associated with breslow thickness at presentation and survival from melanoma. *J. Clin. Oncol.* **27**, 5439–5444 (2009). doi:10.1200/JCO.2009.22.1135 Medline

69. S. Muralidhar, A. Filia, J. Nsengimana, J. Poźniak, S. J. O'Shea, J. M. Diaz, M. Harland, J. A. Randerson-Moor, J. Reichrath, J. P. Laye, L. van der Weyden, D. J. Adams, D. T. Bishop, J. Newton-Bishop, Vitamin D–VDR Signaling Inhibits Wnt/β-Catenin–Mediated Melanoma Progression and Promotes Antitumor Immunity. *Cancer Res.* **79**, 5986–5998 (2019). doi:10.1158/0008-5472.CAN-18-3927 Medline

70. Ł. Galus, M. Michalak, M. Lorenz, R. Stoińska-Swiniarek, D. Tusień Małecka, A. Galus, T. Kolenda, E. Leporowska, J. Mackiewicz, Vitamin D supplementation increases objective response rate and prolongs progression-free time in patients with advanced melanoma undergoing anti–PD-1 therapy. *Cancer* **129**, 2047–2055 (2023). doi:10.1002/cncr.34718 Medline

71. T. Odamaki, J.-Z. Xiao, M. Sakamoto, S. Kondo, T. Yaeshima, K. Iwatsuki, H. Togashi, T. Enomoto, Y. Benno, Distribution of different species of the *Bacteroides fragilis* group in individuals with Japanese cedar pollinosis. *Appl. Environ. Microbiol.* **74**, 6814–6817 (2008). doi:10.1128/AEM.01106-08 Medline

72. M. Barman, D. Unold, K. Shifley, E. Amir, K. Hung, N. Bos, N. Salzman, Enteric salmonellosis disrupts the microbial ecology of the murine gastrointestinal tract. *Infect. Immun.* **76**, 907–915 (2008). doi:10.1128/IAI.01432-07 Medline

73. S. Zelenay, A. G. van der Veen, J. P. Böttcher, K. J. Snelgrove, N. Rogers, S. E. Acton, P. Chakravarty, M. R. Girotti, R. Marais, S. A. Quezada, E. Sahai, C. Reis e Sousa, Cyclooxygenase-Dependent Tumor Growth through Evasion of Immunity. *Cell* **162**, 1257–1270 (2015). doi:10.1016/j.cell.2015.08.015 Medline

74. A. Faustino-Rocha, P. A. Oliveira, J. Pinho-Oliveira, C. Teixeira-Guedes, R. Soares-Maia, R. G. da Costa, B. Colaço, M. J. Pires, J. Colaço, R. Ferreira, M. Ginja, Estimation of rat mammary tumor volume using caliper and ultrasonography measurements. *Lab Anim.* **42**, 217–224 (2013). doi:10.1038/laban.254 Medline

75. A. Cardoso, A. Gil Castro, A. C. Martins, G. M. Carriche, V. Murigneux, I. Castro, A. Cumano, P. Vieira, M. Saraiva, The Dynamics of Interleukin-10-Afforded Protection during Dextran Sulfate Sodium-Induced Colitis. *Front. Immunol.* **9**, 400 (2018). doi:10.3389/fimmu.2018.00400 Medline

76. J. A. McCulloch, J. H. Badger, N. Cannon, R. R. Rodrigues, M. Valencia, J. J. Barb, M. R. Fernandes, A. Balaji, L. Crowson, C. O'hUigin, A. Dzutsev, G. Trinchieri, JAMS - A framework for the taxonomic and functional exploration of microbiological genomic data. bioRxiv 2023.03.03.531026 [Preprint] (2023); https://doi.org/10.1101/2023.03.03.531026.

77. C. N. Spencer, J. L. McQuade, V. Gopalakrishnan, J. A. McCulloch, M. Vetizou, A. P. Cogdill, M. A. W. Khan, X. Zhang, M. G. White, C. B. Peterson, M. C. Wong, G. Morad, T. Rodgers, J. H. Badger, B. A. Helmink, M. C. Andrews, R. R. Rodrigues, A. Morgun,

Y. S. Kim, J. Roszik, K. L. Hoffman, J. Zheng, Y. Zhou, Y. B. Medik, L. M. Kahn, S. Johnson, C. W. Hudgens, K. Wani, P.-O. Gaudreau, A. L. Harris, M. A. Jamal, E. N. Baruch, E. Perez-Guijarro, C.-P. Day, G. Merlino, B. Pazdrak, B. S. Lochmann, R. A. Szczepaniak-Sloane, R. Arora, J. Anderson, C. M. Zobniw, E. Posada, E. Sirmans, J. Simon, L. E. Haydu, E. M. Burton, L. Wang, M. Dang, K. Clise-Dwyer, S. Schneider, T. Chapman, N. A. S. Anang, S. Duncan, J. Toker, J. C. Malke, I. C. Glitza, R. N. Amaria, H. A. Tawbi, A. Diab, M. K. Wong, S. P. Patel, S. E. Woodman, M. A. Davies, M. I. Ross, J. E. Gershenwald, J. E. Lee, P. Hwu, V. Jensen, Y. Samuels, R. Straussman, N. J. Ajami, K. C. Nelson, L. Nezi, J. F. Petrosino, P. A. Futreal, A. J. Lazar, J. Hu, R. R. Jenq, M. T. Tetzlaff, Y. Yan, W. S. Garrett, C. Huttenhower, P. Sharma, S. S. Watowich, J. P. Allison, L. Cohen, G. Trinchieri, C. R. Daniel, J. A. Wargo, Dietary fiber and probiotics influence the gut microbiome and melanoma immunotherapy response. *Science* **374**, 1632–1640 (2021). doi:10.1126/science.aaz7015 Medline

78. J. A. McCulloch, D. Davar, R. R. Rodrigues, J. H. Badger, J. R. Fang, A. M. Cole, A. K. Balaji, M. Vetizou, S. M. Prescott, M. R. Fernandes, R. G. F. Costa, W. Yuan, R. Salcedo, E. Bahadiroglu, S. Roy, R. N. DeBlasio, R. M. Morrison, J.-M. Chauvin, Q. Ding, B. Zidi, A. Lowin, S. Chakka, W. Gao, O. Pagliano, S. J. Ernst, A. Rose, N. K. Newman, A. Morgun, H. M. Zarour, G. Trinchieri, A. K. Dzutsev, Intestinal microbiota signatures of clinical response and immune-related adverse events in melanoma patients treated with anti-PD-1. *Nat. Med.* **28**, 545–556 (2022). doi:10.1038/s41591-022-01698-2 Medline

79. B. Li, C. N. Dewey, RSEM: Accurate transcript quantification from RNA-Seq data with or without a reference genome. *BMC Bioinformatics* **12**, 323 (2011). doi:10.1186/1471-2105-12-323 Medline

80. A. Subramanian, P. Tamayo, V. K. Mootha, S. Mukherjee, B. L. Ebert, M. A. Gillette, A. Paulovich, S. L. Pomeroy, T. R. Golub, E. S. Lander, J. P. Mesirov, Gene set enrichment analysis: A knowledge-based approach for interpreting genome-wide expression profiles. *Proc. Natl. Acad. Sci. U.S.A.* **102**, 15545–15550 (2005). doi:10.1073/pnas.0506580102 Medline
